# Pentamidine Alleviates Inflammation and Lipopolysaccharide-Induced Sepsis by Inhibiting TLR4 Activation *via* Targeting MD2

**DOI:** 10.3389/fphar.2022.835081

**Published:** 2022-02-23

**Authors:** Siru Wu, Cong Lin, Tianshu Zhang, Bo Zhang, Yushan Jin, Hongshuang Wang, Hongyuan Li, Yibo Wang, Xiaohui Wang

**Affiliations:** ^1^ Laboratory of Chemical Biology, Changchun Institute of Applied Chemistry, Chinese Academy of Sciences, Changchun, China; ^2^ Department of Applied Chemistry and Engineering, University of Science and Technology of China, Hefei, China; ^3^ Department of Immunology and Department of Cell & Systems Biology, University of Toronto, Toronto, ON, Canada; ^4^ Beijing National Laboratory for Molecular Sciences, Beijing, China

**Keywords:** Toll-like receptor 4, myeloid differentiation protein 2, pentamidine, inflammation, sepsis

## Abstract

Toll-like receptor 4 (TLR4) is a pattern-recognition receptor (PRR) that can recognize lipopolysaccharides (LPS) and initiate the immune response, to protect the body from infection. However, excessive activation of TLR4 induced by LPS leads to substantial release of pro-inflammatory factors, which may bring a cytokine storm in the body and cause severe sepsis. Existing molecules specialized in sepsis therapy are either in clinical trials or show mediocre effects. In this study, pentamidine, an approved drug used in the treatment of trypanosomiasis, was identified as a TLR4 antagonist. Saturation transferred difference (STD)-NMR spectra indicated that pentamidine directly interacted with TLR4’s co-receptor myeloid differentiation protein 2 (MD2) *in vitro*. Cellular thermal shift assay (CETSA) showed that pentamidine binding decreased MD2 stability, which was supported by *in silico* simulations that pentamidine binding rendered most regions of MD2 more flexible. Pentamidine was found to inhibit the formation of the TLR4/MD2/MyD88 complex and the activation of the TLR4 signaling axes of NF-κB and MAPKs, therefore blocking LPS-induced TLR4 signaling downstream of the pro-inflammatory factors NO, TNF-α, and IL-1β. The bioisosteric replacement of the methylene group at the center 13′ site of pentamidine by the ether oxygen group significantly decreased its interactions with MD2 and abolished its TLR4 antagonist activity. Furthermore, pentamidine enhanced the survival rate of septic mice and exerted an anti-inflammatory effect on organs. All these data provide strong evidence that pentamidine may be an effective drug in alleviating inflammation and sepsis.

## 1 Introduction

During response to invading pathogens, macrophages play a key role in reacting to pathogens and infection by trigging inflammation, phagocytosis, and microbial activity (López-Collazo et al., 2014). Substantial invasion of pathogens leads to sepsis, which is the overwhelming activation and dysregulation of the immune response. As a major cause of mortality, sepsis has affected over 30 million people worldwide by causing cytokine storms and further leading to organ dysfunction (Huang et al., 2019). In response to sepsis, Toll-like receptor 4 (TLR4) has been discovered to play a crucial role in activating an immune response (Raymond et al., 2017). TLR4 is a pattern recognition receptor (PRR) that can recognize pathogen-associated molecular patterns (PAMPs) and play a defensive role in the innate immune system in the process of resisting invading pathogens (Janeway and Medzhitov, 2002). Lipopolysaccharides (LPS) are a major component of the cell wall of Gram-negative bacteria and exhibit strong immunostimulatory activity by targeting the TLR4 signaling axis and triggering sepsis inside the body (Takeda and Akira, 2015). The activation of the TLR4 response to LPS relies on its accessory protein, myeloid differentiation protein 2 (MD2) (Shimazu et al., 1999), which has a β-cup fold structure composed of two antiparallel β sheets to form a large hydrophobic pocket for ligand binding (Kim et al., 2007; Park et al., 2009). In the course of sepsis, macrophages were activated by LPS through the TLR4/MD2 and releasing the inflammatory factors. Previous evidence showed that MD2^−/−^ mice survived endotoxic shock and were hyporesponsive to LPS (Nagai et al., 2002). Therefore, targeting MD2 on macrophages to block the binding of LPS against inflammation has become a valuable strategy in the treatment of sepsis (Chen et al., 2018).

There is a growing interest in drug repositioning to find novel therapeutic indications for drugs on the market (Wang et al., 2013; Peng et al., 2019; Zhang et al., 2021). Therefore, the discovery of approved drugs to modulate TLR4 activity would be a cost-effective and practical way to cure sepsis. Pentamidine is an antiprotozoal agent with significant therapeutic effects in the first stage of Human African trypanosomiasis, antimony-resistant leishmaniasis, and malaria (Wispelwey and Pearson, 1991; Porcheddu et al., 2012). Herein, pentamidine was identified as a TLR4 antagonist by targeting MD2 and inhibited the formation of the TLR4/MD2/MyD88 complex and the activation of TLR4 downstream NF-κB and MAPKs, therefore suppressing LPS-induced pro-inflammatory factor overproduction. An *in vivo* animal study proved that pentamidine alleviated cytokine storms and organ inflammation in mice. Furthermore, pentamidine improved the survival rate of septic mice. These data imply the potential of pentamidine in treating sepsis.

## 2 Manuscript Formatting

### 2.1 Materials and Methods

#### 2.1.1 Materials

Pentamidine isethionate salt, crystal violet, LPS, 2, 3-diaminonaphthalene, paraformaldehyde, and mouse IgG-horseradish peroxidase conjugate were purchased from Sigma-Aldrich. Ultrapure LPS, HEK Blue hTLR4 cells, and HEK-Blue Selection were purchased from Invivogen. The Phospha-Light™ SEAP Reporter Gene Assay System was purchased from Applied Biosystems. The Dual-Glo Luciferase Assay System was purchased from Promega. Protease inhibitor cocktails and phosphatase inhibitors were purchased from Roche. Super Signal West Pico PLUS Chemiluminescent Substrate, cell culture media, and IL-1β mouse ELISA kits were purchased from Thermo Fisher Scientific. Hifair III 1st Strand cDNA Synthesis SuperMix for qPCR and Hieff qPCR SYBR Master Mix were obtained from Yeasen Biotech Co., Ltd. PCR primers were purchased from Comate Bioscience Co., Ltd. Cell lysis buffer for Western blotting and IP, CCK-8 Kit, RIPA lysis buffer, and TNF-α ELISA kit were purchased from Beyotime Biotechnology. Mouse TNF (Mono/Mono) ELISA Set was purchased from BD Biosciences. Primary TLR4 and MD2 antibodies were purchased from Abcam. Primary antibodies targeting MyD88, p38 MAPK, NF-κB p65, ERK (1/2), IKK-β, SAPK/JNK, phospho-NF-κB p65, phospho-ERK (1/2), phospho-SAPK/JNK, phospho-IKK-α/β, and phospho-p38 MAPK were purchased from Cell Signaling Technology. Macrophage 264.7 cells were obtained from American Type Culture Collection. Microglial BV-2 cells were obtained from the China Center for Type Culture Collection.

#### 2.1.2 MD2 Expression and Purification

MD2 expression and purification were performed as described previously (Park et al., 2009; Wang et al., 2012). Briefly, MD2 baculovirus was conducted by co-transfection of SF-9 insect cells with the MD2-pAcGP67A vector and bright linearized baculovirus DNA, as described by the manufacturer’s protocol (BD Bioscience). After 2–3 rounds of amplification, the MD2 baculovirus suspension reached a titer of ∼10^8^ virus particles/ml and was used to transfect High 5 insect cells to the secretion of MD2 in the medium. Three to four days after transfection, the medium was collected, and MD2 protein was purified by IgG Sepharose affinity purification.

#### 2.1.3 Cellular Thermal Shift Assay

CETSA was performed as described previously (Molina et al., 2013). RAW264.7 cells were seeded in 100 mm culture dishes at a density of 1 × 10^6^ cells/ml. After 24 h, the cells were washed with ice-cold PBS. The cell pellets were resuspended in RIPA lysis buffer with a complete protease inhibitor cocktail. Cell lysates were prepared by centrifugation at 12,000 rpm for 15 min at 4°C, and then the supernatant was collected. 400 μM pentamidine or vehicle control (sterile water) was incubated with RAW264.7 cell lysate supernatant for 2 h at room temperature. The respective lysates were divided into smaller (60 μl) aliquots and heated simultaneously at different temperatures (41, 44, 47, 50, 53, 56, 59, and 62°C, and one aliquot was kept at room temperature as the control for each) for 5 min with a LongGene A200 thermal cycler followed by cooling for 3 min at room temperature. Lysates were centrifuged at 20,000 *g* for 20 min at 4°C to separate the soluble fractions from precipitates. The supernatant was transferred to new microtubes with ×5 loading buffer, respectively, and boiled at 100°C in a metal bath for 5 min for Western blotting.

#### 2.1.4 Saturation Transferred Difference-NMR Measurement

NMR spectra were acquired in a Bruker Avance III spectrometer operating at a proton frequency of 600 MHz with a conventional inverse 5 mm probe head with z-gradients at 25°C using standard Bruker pulse programs. Samples containing 1 mM pentamidine in the absence or presence of MD2 (4 μM) in D_2_O buffer (75 mM potassium phosphate, 150 mM sodium chloride, pH 7.5) were used for NMR spectroscopy data acquisition. The selective pulse enables the saturation of signals of proteins and results in a rapid spread of magnetization over the entire protein, which consists of protons that are closely coupled by dipole–dipole interactions. Intermolecular transfer of magnetization from protein to binding ligand leads to the chemical exchange and transfer of progressive saturation of the ligand so that the motifs of pentamidine interacting with MD2 are speculated from the STD-NMR spectrum (Mayer and Meyer, 1999). When binding to the receptor, only the signals of hydrogen that are in close contact with the protein (≤5 Å) and receive magnetization transfer will appear in the difference spectrum after subtraction. The signals will be more intense when hydrogens are closer to the protein, owing to a more efficient saturation transfer (Viegas et al., 2011).

#### 2.1.5 Cell Culture

Macrophage RAW 264.7 cells were grown in supplemented RPMI (Roswell Park Memorial Institute) 1,640 (10% FBS, 50 unit ml^−1^ penicillin, and 50 μg ml^−1^ streptomycin). RAW264.7 cells were detached from the flask by trypsin digestion when ∼80% confluence was reached. BV-2 murine microglial cells were grown in supplemented DMEM (Dulbecco’s modified Eagle medium) (10% FBS, 50 unit ml^−1^ penicillin, and 50 μg ml^−1^ streptomycin). The cells were detached from the dishes by the cell lifter when ∼80% confluence was reached. Primary microglia and astrocytes were isolated and cultured as described in previous reports Wang et al., 2013; Peng et al., 2019.

#### 2.1.6 Nitric Oxide Assay

RAW 264.7 cells were seeded at a density of 8 × 10^4^ cells/well in 96-well plates. BV-2 cells, primary microglial cells, and primary astrocytes were seeded at a density of 4 × 10^4^ cells/well in 96-well plates. After 24 h of incubation, the complete medium was replaced with a medium without FBS before adding LPS and different concentrations of the compound. After 24 h of treatment, 100 μl supernatant of the cell culture medium was transferred to flat black 96-well microfluor plates, and then 10 μl 2, 3-diaminonaphthalene (0.05 mg ml^−1^ in 0.62 M HCl) was added to each well. The plates were placed on a shaker for 15 min to incubate. After incubation, 5 µl of 3 M NaOH was added to each well to stop the reaction. The fluorescence of each well was measured with excitation at 360 nm and emission at 430 nm through a SYNERGY H1 microplate reader.

#### 2.1.7 Crystal Violet Staining

After the treatment, the cells were fixed with 4% paraformaldehyde for 5 min, the supernatant was discarded, and the cells were stained with 0.05% crystal violet for 15 min. After staining, the medium was discarded, and the cells were washed thrice with tap water. The remaining water was discarded before adding 200 μl ethanol, and then the plates were placed on a shaker for 20 min at room temperature. Absorbance at 540 nm was measured using a SYNERGY H1 microplate reader.

#### 2.1.8 Cell Counting Kit-8 Assay

HEK Blue hTLR4 cells were cultured and treated as indicated in the SEAP assay. After 24 h of treatment, 20 μl of CCK-8 solution was added to each well in 96-well plates, and the cells were incubated in 5% CO_2_, 37°C incubator for 2 h. Absorbance was detected at the wavelength of 450 nm with 650 nm as the reference wavelength.

#### 2.1.9 Synthesis of Pentamidine Analog Penta-2

Common reagents and materials for synthesis were purchased from Energy Chemical (Shanghai, China) and used as received without further purification unless otherwise stated. The melting points were measured using a Yuhua X-5 microscope melting point analyzer. High-resolution mass spectrometer (HRMS) data were acquired in the positive ion mode using a Thermo Fisher LTQ ORBITRAP XL with an electrospray ionization (ESI) source. Nuclear magnetic resonance (NMR) spectra were acquired on the Bruker AV-300 spectrometer (300 MHz 1H, 75 MHz 13°C). Chemical shifts (δ) are expressed in ppm downfield from tetramethylsilane (TMS) using non-deuterated solvent present in the bulk deuterated solvent (CDCl3: 1H 7.26 ppm, 13°C 77.16 ppm; DMSO-d6: 1H 2.50 ppm, 13°C 39.43 ppm; MeOD-d4: 1H 3.31 ppm, 13 C 49.05 ppm). Data are represented as follows: chemical shift, multiplicity (*s* = singlet, *d* = doublet, *t* = triplet, *m* = multiplet, *br* = broad), coupling constant in Hertz (Hz), and integration.

Synthesis of 4,4′-{[oxybis (ethane-2,1-diyl)] bis (oxy)} dibenzonitrile (compound 3). To a solution of 4-hydroxybenzonitrile (compound 1, 1.20 g, 10 mmol) in N-methylpyrrolidone (15 ml), 1-chloro-2-(2-chloroethoxy)ethane (compound 2, 0.72 g, 5 mmol) and K_2_CO_3_ (2.08 g, 10 mmol) were added at room temperature. The reaction mixture was heated to 130°C and stirred for 2 h. Then, the reaction mixture was poured into an ice-water mixture (200 ml), and a large amount of white solid was generated. The solid was filtered and dried under reduced pressure to achieve compound 3 (0.70 g, 45%), which was used for the next step without further purification: 1H NMR (300 MHz, CDCl3): *δ* = 7.55 (s, 4H), 6.93 (s, 4H), 4.17 (s, 4H), 3.93 (s, 4H) ppm. 13C NMR (75 MHz, CDCl3): *δ* = 161.89, 161.89, 133.91, 133.91, 133.91, 133.91, 119.06, 119.06, 115.20, 115.20, 115.20, 115.20, 104.14, 104.14, 69.59, 69.59, 67.62, 67.62 ppm.

Synthesis of 4,4′-{[oxybis (ethane-2,1-diyl)] bis (oxy)} dibenzonitrile (compound 4). To a solution of dry EtOH (20 ml), freshly prepared HCl (gas) was bubbled for 8 h on ice bath. Then, compound 3 (0.70 g, 2.3 mmol) was added to the prepared solution (20 ml), slowly warmed to ambient temperature, and stirred for 4 days. The reaction mixture was concentrated under reduced pressure to achieve crude compound 4, which was unstable and used for the next step without further purification.

Synthesis of 4,4′-{[oxybis (ethane-2,1-diyl)] bis (oxy)} dibenzimidamide (penta-2). To a solution of dry EtOH (30 ml), freshly prepared NH_3_ (gas) was bubbled for 8 h at −20°C. Then, compound 4 was added to the saturated ethanolic ammonia solution (30 ml). The reaction mixture was sealed and slowly warmed to ambient temperature. After vigorous stirring for 2 days, a large amount of white solid was generated. The reaction mixture was filtered, and the filter cake was dispersed to a solution of aqueous NaOH (60 ml, 1 M). After stirring for 1 h, the solid was filtered, and the filter cake was washed with water (10 ml) and then dried under a high vacuum to yield penta-2 (0.21 g, 27% from compound 3) as a white solid: M.p. 170.1–172.5°C [lit. (Maciejewska et al., 2006) 256–258°C as a tetrahydrochloride salt]. 1H NMR (300 MHz, MeOD-d_4_): *δ* = 7.67 (d, *J* = 8.7 Hz, 4H), 6.98 (d, *J* = 8.7 Hz, 4H), 4.19 (m, 4H), 3.03 (m, 4H), 3.92 (m, 4H) ppm; 13 C NMR (75 MHz, MeOD-d_4_): *δ* = 167.38, 167.38, 162.48, 162.48, 129.53, 129.53, 129.53, 129.53, 115.46, 115.46, 115.46, 70.93, 70.93, 68.80, 68.80 ppm; HRMS (ESI-Orbitrap) m/z: [M + H]^+^ calcd for C_18_H_23_N_4_O_3_^+^ 343.1765; found 343.1768.

#### 2.1.10 Secreted Embryonic Alkaline Phosphatase Assay

HEK Blue hTLR4 cells were co-transfected with the human TLR4 gene, MD2/CD14 co-receptor genes, and a secreted embryonic alkaline phosphatase reporter gene under the control of the NF-κB signaling pathway. The activation of TLR4/MD2 can be monitored by measuring NF-κB activity with a microplate reader. HEK Blue hTLR4 cells were grown in supplemented DMEM (10% FBS, 50 unit ml^−1^ penicillin, 50 μg ml^−1^ streptomycin, and 1× HEK-Blue selection). The cells were seeded at a density of 4 × 10^4^ cells/well in 96-well plates for drug testing. After 24 h incubation, the medium was replaced with Opti-MEM (Opti-Minimal Essential Medium) (0.5% FBS, 50 unit ml^−1^ penicillin, 50 μg ml^−1^ streptomycin, and 1× non-essential amino acid). LPS (20 ng ml^−1^) and different concentrations of pentamidine or penta-2 were added to the medium. After 24 h, NF-κB activity was measured through the Phospha-Light SEAP Reporter Gene Assay System according to the manufacturer’s instructions.

#### 2.1.11 Dual-Luciferase NF-κB Reporter Assay

BV-2 NF-κB luciferase reporter cells were constructed as described in a previous study (Wang et al., 2012). Briefly, the Firefly luciferase gene was placed under the control of the NF-κB transcriptional response element, and the constitutively expressing Renilla luciferase was placed under the CMV promotor. Upon stimulation with LPS, the TLR4/MD2 complex was activated, thus initiating the activation of NF-κB and the production of two kinds of luciferase enzymes. The ratio of Firefly luciferase activity to Renilla luciferase activity represents NF-κB activity. BV-2 NF-κB luciferase reporter cells were grown in supplemented DMEM (10% FBS, 50 unit ml^−1^ penicillin, 50 μg ml^−1^ streptomycin, and 2 μg ml^−1^ puromycin). The cells were seeded at a density of 1 × 10^4^ cells/well in 96-well plates for drug testing. After 24 h incubation, the medium was replaced with Opti-MEM (0.5% FBS, 50 unit ml^−1^ penicillin, 50 μg ml^−1^ streptomycin, and 1× non-essential amino acid). Different concentrations of pentamidine or penta-2 were added. After 24 h of treatment, NF-κB activity was measured through the Dual-Glo Luciferase Assay System according to the manufacturer’s instructions.

#### 2.1.12 *In Silico* Simulation

##### 2.1.12.1 System Preparation and Docking

Pentamidine and penta-2 were drawn using GaussView 6 (GaussView et al., 2016) and optimized by Gaussian 09 (Gaussian 09 et al., 2016) software through the B3LYP/6-31G (d, p) basis set (Becke, 1988; Lee et al., 1988; Miehlich et al., 1989). The crystal structure of MD2 was extracted from the mouse TLR4/MD2 complex (PDB ID: 2Z64) (Kim et al., 2007). The missing hydrogen atoms were added at pH 7.0 using Maestro (Schrödinger, 2021-2: Maestro). Autodock Vina 1.1.2 was used for molecular docking in a box of 46 × 58 × 53 Å^3^, which covers MD2 completely (Trott O, 2010). Pentamidine or penta-2 was also docked in monomeric TLR4/MD2 (PDB ID: 2Z64) or dimeric TLR4/MD2 (PDB ID: 3VQ2). The Iterated Local Search Globule Optimizer was applied to locate the most favorable binding site (Baxter, 1981; Blum et al., 2008). MD2 was considered rigid and pentamidine or penta-2 was regarded as semi-flexible during the docking process. Twenty docking poses were generated and ranked according to their affinity with MD2, of which the best docking pose with the best affinity to MD2 was selected for further simulations.

##### 2.1.12.2 Molecular Dynamic Simulation

MD2 alone (apo-MD2) and the best docking pose of pentamidine or penta-2 with MD2 were further investigated through molecular dynamic simulations performed by the NAMD2.12 package (Phillips et al., 2005) with the AMBER ff03 force field (Duan et al., 2003; Wang et al., 2004). The atomic charges of pentamidine and penta-2 were optimized and fitted by R.E.D. based on quantum mechanics calculations (Dupradeau et al., 2010). Other atomic parameters were treated with the general Amber force field (GAFF) (Wang et al., 2004). All solutes were solvated in a TIP3P model of a cubic water box with a distance of 10 Å between the protein and the edge of the water box. Na^+^ and Cl^−^ ions were added into the water box to neutralize the system and fill the system with a concentration of 0.15 M NaCl to mimic the physiological condition. Energy minimization was performed for 5,000 steps, and the system was subsequently heated to 310 K in 310 ps with 1 ns equilibration. The system was further run in the isothermal-isobaric (NPT) ensemble at a temperature of 310 K for 200 ns. The SHAKE algorithm was applied to restrain all bonds involving hydrogen (Blum et al., 2008). The Particle mesh Ewald (PME) summation method was applied to calculate long-range electrostatic interactions (Darden et al., 1993). The temperature was kept at 310 K using Langevin dynamics with a collision frequency of 5 ps^−1^. The pressure was set at 1 atm with the Nosé–Hoover Langevin piston method (Feller et al., 1995).

The RMSD (root-mean-square deviation) and RMSF (root-mean-square fluctuation) analyses were performed through VMD (Humphrey et al., 1996) and Bio3D packages (Grant et al., 2006), respectively. The interactions between MD2 and pentamidine were analyzed by LigPlot^+^ (Laskowski and Swindells, 2011) and PyMol (PyMol) software. The logP of pentamidine or penta-2 was predicted through RDKit software (Landrum).

#### 2.1.13 Co-Immunoprecipitation

RAW 264.7 cells were seeded at 1 × 10^6^ cells/well in 100 mm culture dishes. After 24 h incubation, the cells were stimulated with LPS (20 ng ml^−1^) and the indicated concentrations of pentamidine for 1 h and subsequently washed twice with ice-cold PBS. Later, the cells were lysed in 1 ml Co-IP lysis buffer (25 mM Tris pH 8.0, 150 mM KCl, 5 mM EDTA, 0.5% NP-40) with complete protease inhibitor cocktail, 1 mM DTT, and 1 mM PMSF by incubating on ice for 30 min. Cell debris was removed by centrifugation at 12,000 g at 4°C for 12 min. Corresponding primary antibody was added, and samples were gently rotated on a shaker at 4°C overnight incubation. Washed magnetic beads were then added, and the samples were gently rotated and incubated on a shaker at room temperature for 1 h. The magnetic beads were washed twice with PBS and boiled with 50 μl 2× SDS sample buffer at 100°C for 8 min for Western blotting.

#### 2.1.14 Signaling Pathway Sample Preparation

RAW 264.7 cells were cultured as described above in 100 mm culture dishes, and after incubation at 37°C for 24 h, the corresponding concentrations of pentamidine were added 1 h before the stimulation with LPS (20 ng ml^−1^). After being stimulated with LPS for 1 h, the cells were washed twice with ice-cold PBS, lysed in cell lysis buffer with protease inhibitor and phosphatase inhibitor on ice, and transferred to a microtube for centrifugation at 12,000 rpm, 4°C for 10 min. After centrifugation, the supernatant was diluted and the concentrations of protein were measured through the BCA kit before being boiled with 2× SDS sample buffer at 100°C for 8 min for Western blotting.

#### 2.1.15 Western Blotting

Protein samples were first separated by SDS-PAGE and then transferred to PVDF membranes. After blocking with 5% non-fat dry milk for 1.5 h, the membranes were then incubated with corresponding primary antibodies at 4°C overnight. The membranes were washed five times in Tris-buffered saline with 0.05% Tween 20 (TBST) for 5 min each time and incubated for 1 h at room temperature with secondary antibody-HRP conjugate, followed by another five washes with TBST. Proteins were detected using Tanon-5200 Multi while reacting with Super-Signal West Pico Chemiluminescent Substrate. ImageJ was used for later densitometric analysis.

#### 2.1.16 qRT-PCR

RAW 264.7 cells were grown in supplemented RPMI 1640 (10% FBS, 50 unit ml^−1^ penicillin, 50 μg ml^−1^ streptomycin). The cells were seeded at a density of 1 × 10^6^ cells/ml and 2 ml/well in 6-well plates. After overnight incubation, the medium was replaced with RPMI 1640 medium. LPS (20 ng ml^−1^), and the indicated concentrations of pentamidine were added. After 24 h, total RNA was extracted by TRIzol (TABAKA) according to the manufacturer’s instructions. Hifair Ⅲ 1st Strand cDNA Synthesis SuperMix for qPCR (Yeasen) was used to synthesize cDNA according to the manufacturer’s instructions. The primers for TNF-α, IL-1β, and Rpl27 were purchased from Comate Bioscience Co., Ltd. (Jilin, China). qPCR was performed on a TOptical Real-Time qPCR Thermal Cycler (Analytik Jena) using the SYBR Green method. Rpl27 was set as the reference gene (Thomas et al., 2014). The data were analyzed by the ΔΔC_t_ method. Primer Sequences are shown in Table 1.

**TABLE 1:** Primer sequences of IL-1β, TNF-α, and Rpl27.

Gene	Forward/reverse	Sequence (5′–3′)
IL-1β	Forward	CCA​CCT​TTT​GAC​AGT​GAT​GA
	Reverse	GAG​ATT​TGA​AGC​TGG​ATG​CT
TNF-α	Forward	CCC​TCC​AGA​AAA​GAC​ACC​ATG
	Reverse	GCC​ACA​AGC​AGG​AAT​GAG​AAG
Rpl27	Forward	AAG​CCG​TCA​TCG​TGA​AGA​ACA
	Reverse	CTT​GAT​CTT​GGA​TCG​CTT​GGC

#### 2.1.17 TNF-α and IL-1β ELISAs

TNF-α and IL-1β were measured using mouse ELISA kits according to the manufacturer’s instructions.

#### 2.1.18 *In Vivo* Study

##### 2.1.18.1 Subjects

SPF male C57BL/6 mice (18–21 g, 6–8 weeks) were purchased from Liaoning Changsheng Biotechnology Co., Ltd. All mice were housed in temperature (18–21°C) and light-controlled (12 h light: dark cycle; lights on at 08:00 a.m.) room with standard rodent food and water, and were allowed to habituate to the environment for at least 1 week before experimentation. All animal-handling procedures were approved by the Institutional Animal Care and Use Committee (IACUC) of Changchun Institute of Applied Chemistry, Chinese Academy of Sciences (CIAC 2021-69).

##### 2.1.18.2 Sepsis Model

A single dose of LPS [25 mg/kg, intraperitoneal injection (*i.p.*)] was used to induce acute sepsis (Yan et al., 2020). Pentamidine (10 mg/kg, *i.p.*) was administered once daily for 4 consecutive days after the LPS challenge. The survival rate of mice was recorded for 7 days.

##### 2.1.18.3 Pro-Inflammatory Factor Measurement and H&E Staining

Male C57BL/6 mice were intraperitoneally injected with saline, LPS (25 mg/kg), or LPS (25 mg/kg) + pentamidine (10 mg/kg). After 24 h, blood was collected and placed at room temperature for 2 h. Blood samples were then centrifuged at 1,000 g for 10 min at 4°C to separate serum. The serum was transferred to new tubes and stored at −80°C for ELISA. The concentrations of TNF-α and IL-1β in serum from septic mice were measured by ELISA kits following the manufacturer’s instructions.

Septic mice were anesthetized. The liver, kidney, and lung were collected. These organs were fixed with formalin, embedded in paraffin, sectioned by microtome, and stained with hematoxylin and eosin (H&E). The tissue sections were then observed and imaged under a microscope. Images were taken at ×400 magnification.

#### 2.1.19 Statistical Analysis

Data are expressed as the mean ± S.E.M, and analysis of variance was carried out using Student’s *t*-test unless otherwise stated. Statistical comparisons are indicated in the figure for clarity, and significance was set at #, *p* < 0.05 versus the control; ##, *p* < 0.01 versus the control; ###, *p* < 0.001 versus the control; ####, *p* < 0.0001 versus the control; *, *p* < 0.05 versus the LPS group; **, *p* < 0.01 versus the LPS group; ***, *p* < 0.001 versus the LPS group; ****, *p* < 0.0001 versus the LPS group; ns indicates no significant differences.

### 2.2 Results

#### 2.2.1 Biophysical Binding of Pentamidine With MD2

**FIGURE 1:**
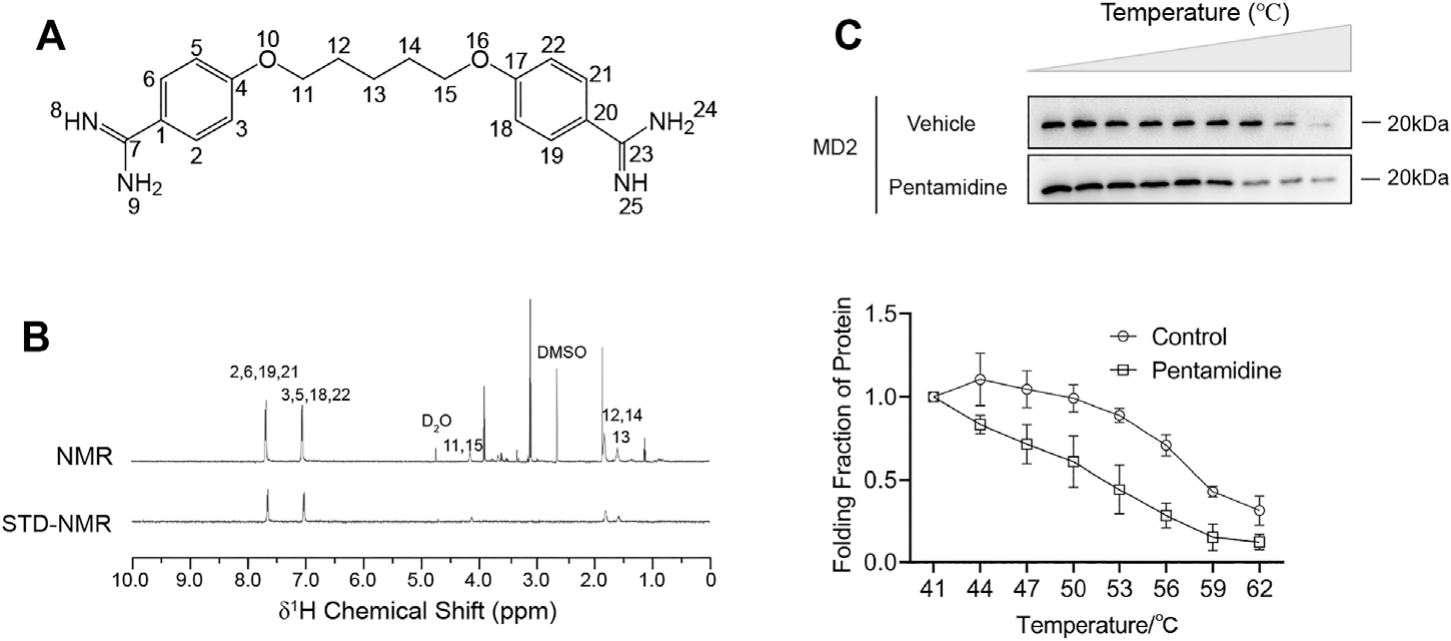
Biophysical characterizations of pentamidine binding with MD2. **(A)** Chemical structure of pentamidine. **(B)** STD-NMR of pentamidine (1 mM) with MD2 (4 μM). 1H NMR assignments of pentamidine were also given. **(C)** Cellular thermal shift assay (CETSA) of MD2 with pentamidine. Pentamidine binding decreases MD2 thermal stability (ΔTm = −5.4 ± 2.4°C). CETSA experiments were performed three times independently, and data were given as the mean ± S.E.M.

In addition to its antiprotozoal action, pentamidine (Figure 1A) has also been reported to alleviate inflammatory responses that contribute to its efficacy against infectious pathogens (Corsini et al., 1992a; Corsini et al., 1992b). However, its molecular mechanism is yet to be discovered. Since TLR4/MD2 plays an important role in inflammatory responses, we first investigated the binding of pentamidine with MD2, which is responsible for ligand binding and the initiation of TLR4 signaling. To determine the interactions between pentamidine and MD2, STD-NMR was performed. Characteristic differences of ligand in its binding mode and dissociative mode are significant in Figure 1B. The exchange between pentamidine and pentamidine with MD2 allows the intermolecular transfer of magnetization from the receptor to MD2 bound pentamidine. Other compounds that may be present but do not bind to the MD2 will not receive any saturation transfer so that their signals will be identical to the intensity on the NMR spectra. After subtraction from the NMR spectra, no signals will appear in the difference spectrum from the non-binding small molecules. Except for the active hydrogens whose signals were not shown in the spectrum, the remaining signals were from the hydrogens of the benzene ring (2′, 6′, 19′, 21′ and 3′, 5′, 18′, 22′ site) and carbon chain (11′, 15′, 12′, 14′, and 13’ site) of pentamidine, which indicated that pentamidine directly interacted with purified MD2 *in vitro* and that the binding sites of pentamidine with MD2 were nearly on the entire molecule (Figure 1B). To investigate whether MD2 is the endogenous target of pentamidine, CETSA of MD2 in cell lysates was performed. As shown in Figure 1C, pentamidine destabilized the *T*_m_ value of MD2 by 5.4 ± 2.4°C compared to the control, supporting that pentamidine directly binds to MD2 in the cellular context. Together, these biophysical characterizations consistently show that MD2 is a direct binding target of pentamidine.

#### 2.2.2 *In Silico* Simulation of Pentamidine Interacting With MD2

**FIGURE 2:**
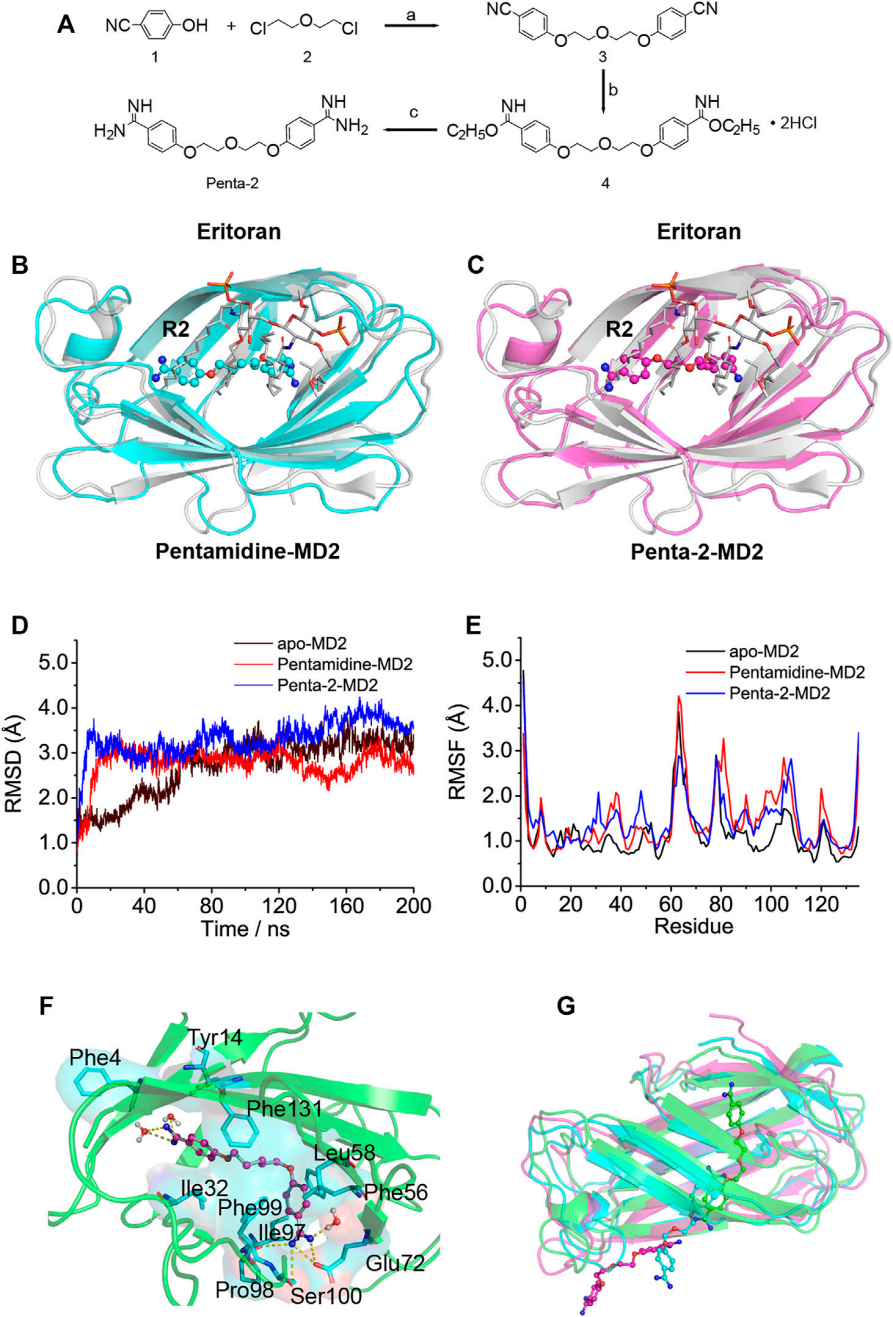
*In silico* simulation of pentamidine and penta-2 interacting with MD2. **(A)** Synthetic route for penta-2. Reagents and conditions: (a) K_2_CO_3_, N-methylpyrrolidone, 130°C; (b) EtOH, HCl (gas), ambient temperature; (c) EtOH, NH_3_ (gas), ambient temperature. **(B,C)** Overlap of the best docking pose of pentamidine **(B)** or penta-2 **(C)** with Eritoran. Eritoran binding location was occupied by pentamidine on its acyl chains R2, and penta-2 on its acyl chains R2. Eritoran–MD2 was extracted from the human TLR4 TV3 hybrid MD2-Eritoran complex (PDB ID: 2Z65) after aligning with compound-docked MD2. MD2 was shown as a cartoon, Eritoran as sticks, and pentamidine and penta-2 as balls-sticks. Eritoran–MD2 was colored as gray, pentamidine–MD2 as green, and penta-2–MD2 as magentas. **(D)** Time evolution of the RMSD of MD2 (apo-MD2) and compound bound MD2 (pentamidine–MD2 and penta-2–MD2) during the MD simulations at 310 K. **(E)** RMSF of MD2 and compound bound MD2. **(F)** The representative binding mode of pentamidine with MD2 at 310 K after molecular dynamic simulation. Pentamidine was shown as a balls-stick model. MD2 was shown as a cartoon model. Key residues of MD2 in interacting with ligands were shown as stick and surface models labeled with residue names. Hydrogen bonds were shown as dashed lines in yellow. Water molecule interacting with pentamidine was represented as a balls-stick model. **(G)** Pathway of penta-2 leaving hydrophobic cavity MD2. MD2 was shown as a cartoon model, and penta-2 was shown as a balls-stick model. The leaving process of penta-2 was shown in the color order of green–blue–magentas.

Considering that the biological activity may be significantly changed upon slight alteration of the structure of the compound, it would be interesting to explore the pentamidine analog (penta-2) which has a substituted ether oxygen group at the center 13’ site and retains the symmetry of pentamidine. The synthesis route of penta-2 is shown in Figure 2A and specified in the methods.

To investigate atomic-detailed interactions between pentamidine or penta-2 and MD2, *in silico* simulations were first conducted. The docking results indicated that both pentamidine (Supplementary Figure S3A) and penta-2 (Supplementary Figure S3B) occupied the LPS binding location of its acyl chains R3′ and R2′. Both pentamidine and penta-2 blocked LPS binding to MD2, and their positions in MD2 were nearly identical. Compared with TLR4/MD2 antagonist Eritoran, pentamidine (Figure 2B) and penta-2 (Figure 2C) occupied the Eritoran binding location of its acyl chain R2, stayed near the end of its acyl chains, and in the deep cavity of MD2. The best docking pose of pentamidine or penta-2 in monomeric TLR4/MD2 and dimeric TLR4/MD2 was also investigated and shown in Supplementary Figures S3C–F, respectively. The docking results of monomeric or dimeric TLR4/MD2 indicated that pentamidine or penta-2 stayed far from the activation interface of TLR4/MD2. As shown in Figure 2D, the root-mean-square deviation (RMSD) analysis of backbone atoms of apo-MD2 and MD2 bound with pentamidine or penta-2 indicated that both systems reached stable states during 200 ns simulations. The RMSD value of apo-MD2 stabilized at around 3.0 Å, and the RMSD value of pentamidine-MD2 stabilized at around 2.7 Å, and the RMSD value of penta-2-MD2 stabilized at around 3.5 Å. To investigate the flexibility changes caused by pentamidine or penta-2, root-mean-square fluctuation (RMSF) analysis was conducted. The binding of pentamidine rendered most regions of MD2 more flexible (Figure 2E), indicating that pentamidine destabilizes MD2. This result is consistent with the experimental CETSA data. Key residues of MD2 interacting with pentamidine were shown as sticks in Figure 2F. Pentamidine formed hydrogen bonds with Glu72, Pro98, Phe100, and the surrounding water molecules. Moreover, pentamidine formed hydrophobic interactions with Phe4, Tyr14, Ile32, Phe56, Leu58, Ile97, Phe99, and Phe131. However, during the process of molecular simulations, penta-2 was found to leave the cavity of MD2 gradually (Figure 2G), which indicated that penta-2 was unstable in the hydrophobic pocket of MD2 and was inclined to leave the hydrophobic cavity, which implies that penta-2 may have poor TLR4 signaling activity. Upon bioisosteric replacement of the methylene group at the 13-position of pentamidine (logP = 2.88) with the ether oxygen group, the lipophilicity of penta-2 (logP = 1.73) greatly decreased, which explains why penta-2 was unstable in hydrophobic cavity MD2 and inclined to stay in a water environment compared to pentamidine.

#### 2.2.3 Pentamidine Inhibits LPS-Induced NF-κB Activation in Cells

**FIGURE 3:**
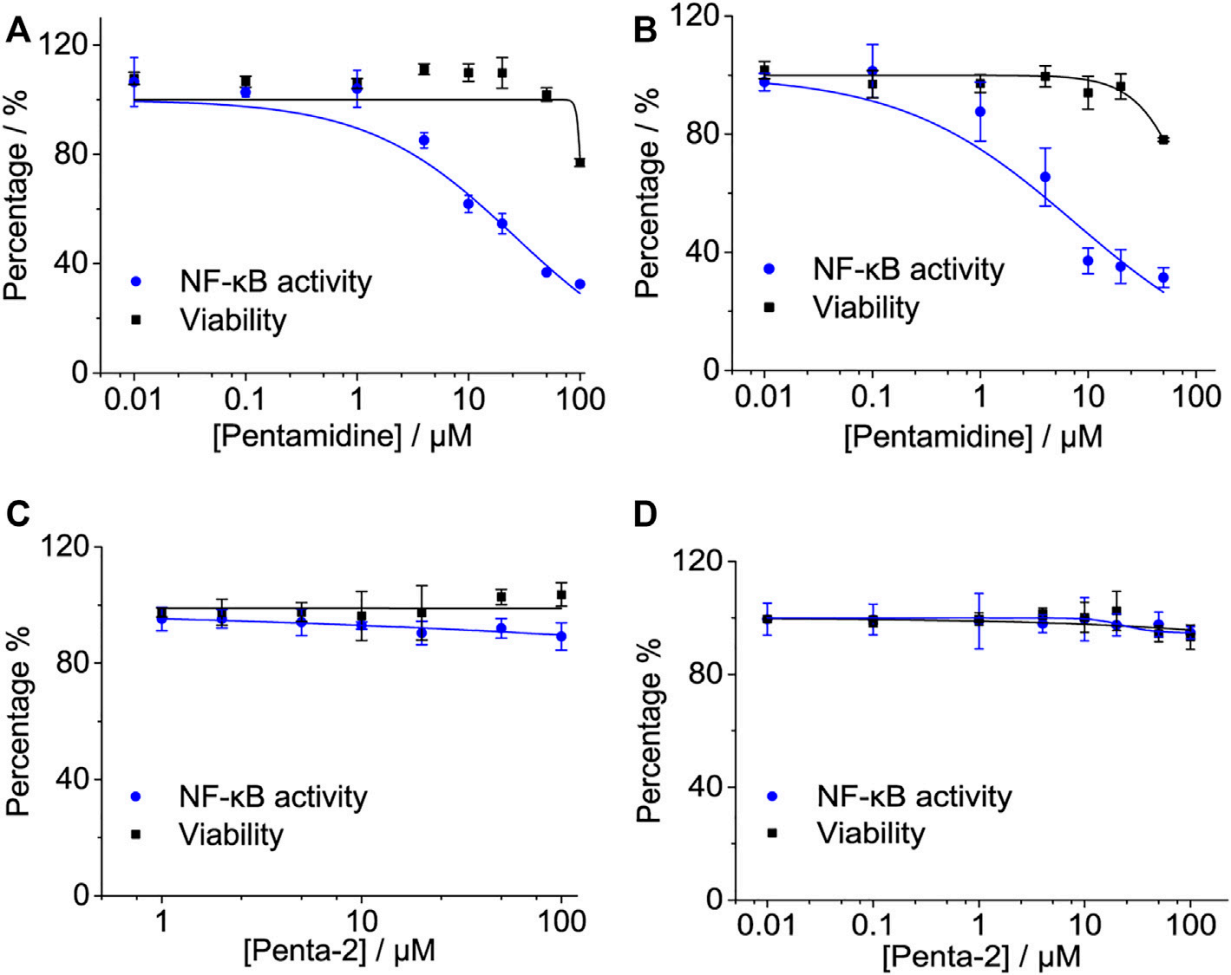
Effects of pentamidine and penta-2 on LPS-induced NF-κB activity. HEK-Blue hTLR4 cells, which over-express human CD14, TLR4, and MD2, were stimulated with 20 ng ml^−1^ LPS and indicated concentrations of pentamidine **(A)** and penta-2 **(C)**. The NF-κB activity was determined by SEAP assay, and cellular viability was measured by CCK-8 Kit. BV-2 NF-κB luciferase reporter cells were treated with 200 ng ml^−1^ LPS and the indicated concentrations of pentamidine **(B)** and penta-2 **(D)**. After 24 h of incubation, the NF-κB activity was determined by the Dual-Glo luciferase assay, and cellular viability was measured by crystal violet staining. All experiments were performed 3–4 times independently, and data were given as the mean ± S.E.M.

NF-κB is a major transcription factor of innate immune responses mediated by TLR4. To examine the effect of pentamidine and its analog on NF-κB activity, the HEK-Blue hTLR4 cell line with a SEAP reporter gene, under the control of an NF-κB responsive element, was employed as the model. Pentamidine was found to inhibit LPS-induced NF-κB activation in a dose-dependent manner, with an IC_50_ of 25.9 ± 4.2 μM (Figure 3A). As no apparent cellular toxicity of pentamidine was observed within 100 μM (Figure 3A), the possibility that cellular toxicity caused the reduction in NF-κB activity was eliminated. In addition to HEK-Blue hTLR4 cells, the effect of pentamidine on NF-κB activity in BV-2 NF-κB luciferase reporter cells was also investigated. Pentamidine was found to inhibit LPS-induced NF-κB activation in a dose-dependent manner, with an IC_50_ of 7.5 ± 2.4 μM (Figure 3B). Similar to that in HEK-Blue hTLR4 cells, pentamidine did not affect cellular viability at concentrations < 50 μM. However, no apparent NF-κB inhibition or toxicity was found in HEK-Blue hTLR4 cells (Figure 3C) or BV-2 NF-κB luciferase reporter cells (Figure 3D) when treated with penta-2.

#### 2.2.4 Pentamidine Inhibits LPS-Induced Pro-Inflammatory Factor NO in Cell Lines

**FIGURE 4:**
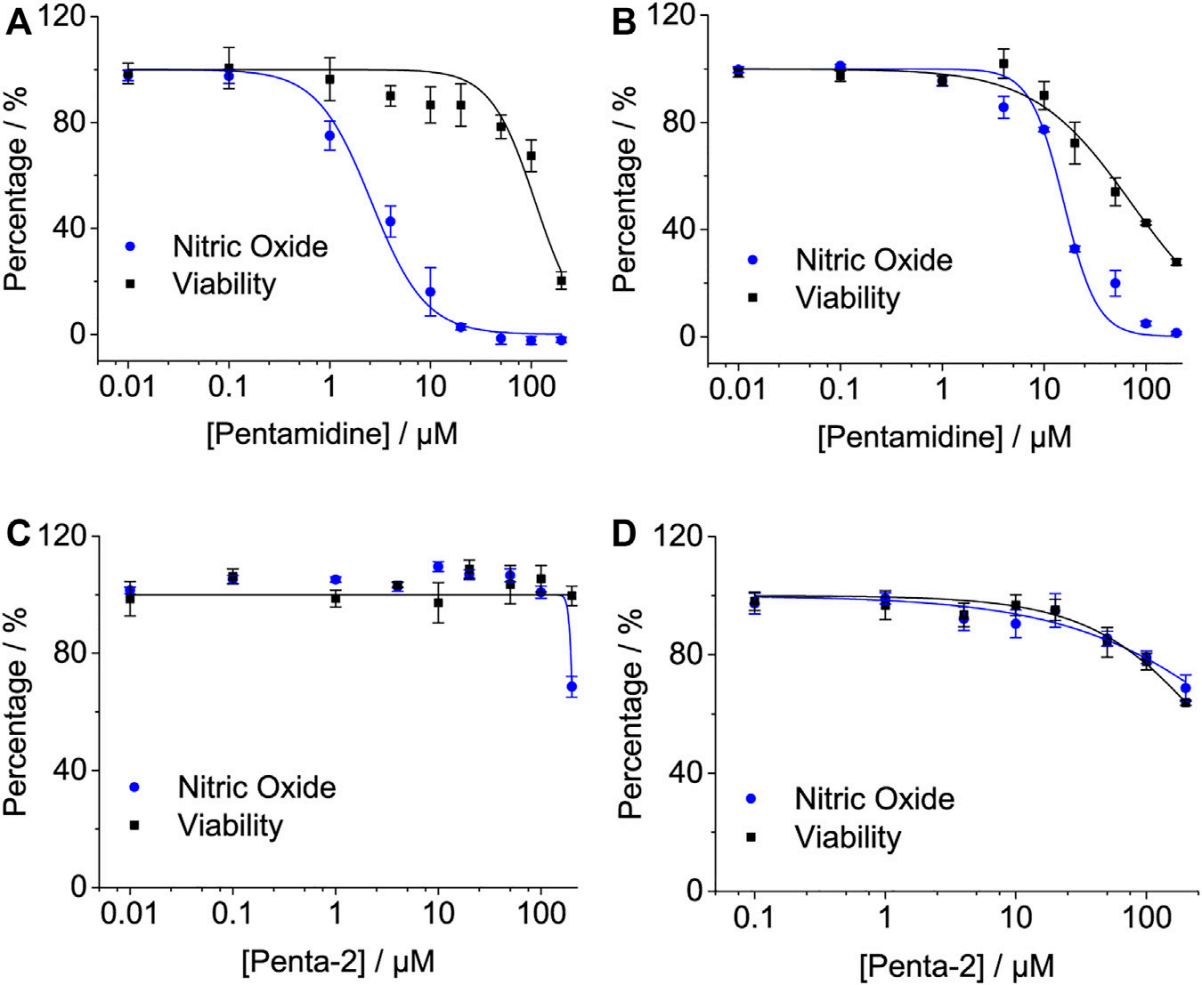
Effects of pentamidine **(A,B)** and penta-2 **(C,D)** on LPS-induced NO overproduction in macrophage RAW 264.7 cells **(A,C)** and BV-2 microglial cells **(B,D)**. Macrophage RAW264.7 and BV-2 cells were treated with LPS and indicated concentrations of pentamidine and penta-2. After 24 h of incubation, NO in the culture supernatant was measured. The NO production of the LPS treated group without pentamidine or penta-2 was set as 100%, respectively. The cellular viability of pentamidine and penta-2 was also measured by crystal violet staining. All experiments were performed 3–4 times independently, and data are given as the mean ± S.E.M.

The activation of TLR4/MD2 by LPS initiates downstream signaling pathways, which brings the release of NO. Pentamidine inhibited LPS-induced NO overproduction in RAW 264.7 macrophages (Figure 4A) and BV-2 microglia (Figure 4B) in a concentration-dependent manner with IC_50_ values of 2.6 ± 0.5 μM and 15.6 ± 0.6 μM, respectively. BV-2 cells are less sensitive to pentamidine than RAW 264.7 cells in inhibiting TLR4 signaling. The IC_50_ values of pentamidine on the viability of RAW 264.7 and BV-2 cells were 109.6 ± 12.2 μM and 71.3 ± 3.4 μM, respectively.

Penta-2 inhibited LPS-induced NO overproduction in RAW 264.7 macrophages (Figure 4C) and BV-2 microglia (Figure 4D) in a concentration-dependent manner with both IC_50_ values of > 200 μM. Penta-2 showed much weaker TLR4 antagonistic activity than pentamidine.

#### 2.2.5 Pentamidine Inhibits LPS-Induced Pro-Inflammatory Factor NO in Primary Cells

**FIGURE 5:**
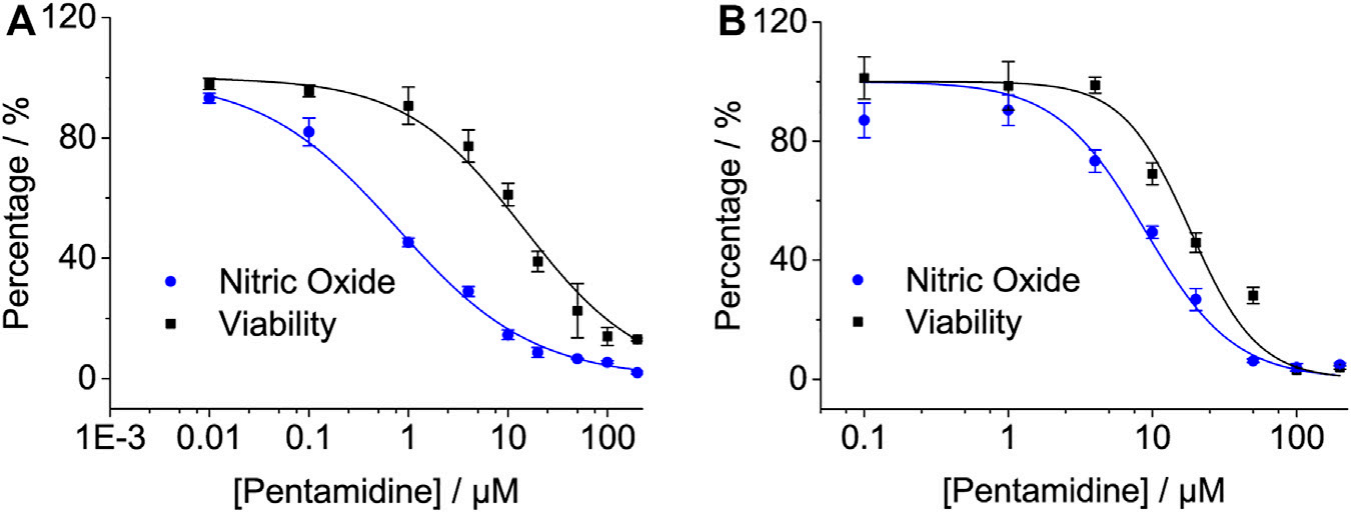
Pentamidine inhibits LPS-induced pro-inflammatory factor NO overproduction in macrophage primary microglia **(A)** and primary astrocyte **(B)**. Cells were treated with LPS and indicated concentrations of pentamidine. After 24 h of incubation, NO in the culture supernatant was measured. The NO production of the LPS treated group without pentamidine was set as 100%. The cellular viability of pentamidine was also measured by crystal violet staining. All experiments were performed 3–4 times independently, and data are given as the mean ± S.E.M.

Primary cells are usually isolated directly from the body, which is much closer to their *in vivo* state and more accurate in elaborating drug function. To investigate the effect of pentamidine on the inhibition of LPS-induced NO overproduction in primary cells, the primary microglia and primary astrocytes were isolated as previously described (Peng et al., 2019) and used. Pentamidine inhibited LPS-induced NO production in a dose-dependent manner in primary microglia, with an IC_50_ of 0.77 ± 0.1 μM (Figure 5A). Meanwhile, the IC_50_ for pentamidine affecting cellular viability in primary microglia was 14.3 ± 1.9 μM (Figure 5A). In primary astrocytes, the IC_50_ values of pentamidine inhibiting LPS-induced NO production and affecting cellular viability were 8.9 ± 1.6 μM and 18.3 ± 2.9 μM (Figure 5B), respectively.

#### 2.2.6 Pentamidine Inhibits LPS-Induced TNF-α and IL-1β Overproduction

**FIGURE 6:**
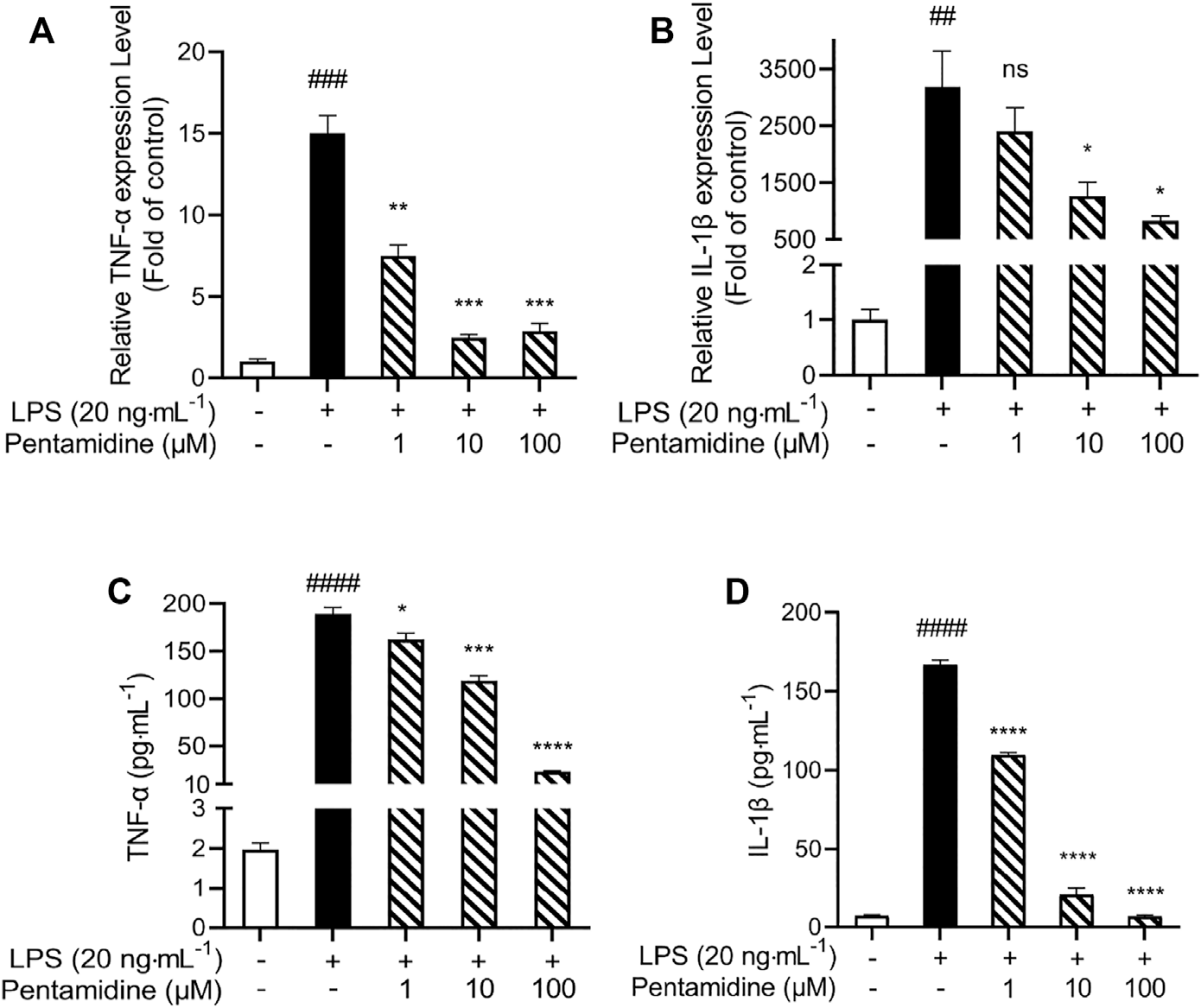
Pentamidine inhibits LPS-induced pro-inflammatory factors TNF-α **(A,C)** and IL-1β **(B,D)** at mRNAs **(A,B)** and protein **(C,D)** level overproduction. RAW264.7 cells were treated with 20 ng ml^−1^ LPS and indicated concentrations of pentamidine for 24 h. Total RNA was extracted and qRT-PCR was performed to measure the expression of TNF-α and IL-1β mRNAs. The TNF-α and IL-1β proteins in the supernatant were measured by ELISA according to the manufacturer’s instructions. All experiments were performed three times independently, and data were given as the mean ± S.E.M. ##, *p* < 0.01 versus the control; ###, *p* < 0.001 versus the control; ####, *p* < 0.0001 versus the control; *, *p* < 0.05 versus the LPS group; **, *p* < 0.01 versus the LPS group; ***, *p* < 0.001 versus the LPS group; ****, *p* < 0.0001 versus the LPS group; ns, not significant.

The pro-inflammatory cytokines TNF-α and IL-1β are crucial downstream products of the innate immune responses, mediated by TLR4. qRT-PCR was performed to investigate whether pentamidine inhibited LPS-induced mRNA expression of TNF-α and IL-1β. Pentamidine inhibited TNF-α (Figure 6A) and IL-1β (Figure 6B) mRNA overexpression in a concentration-dependent manner. Moreover, TNF-α and IL-1β ELISA showed that pentamidine inhibited LPS-induced TNF-α protein (Figure 6C) and IL-1β protein (Figure 6D) overproduction, which is consistent with qRT-PCR results as indicated above. All these data indicated that pentamidine inhibits LPS-induced TLR4 signaling and overproduction of the downstream pro-inflammatory cytokines TNF-α and IL-1β.

#### 2.2.7 Pentamidine Inhibits LPS-Induced TLR4/MD2/MyD88 Complex and the Activation of NF-κB and MAPKs

**FIGURE 7:**
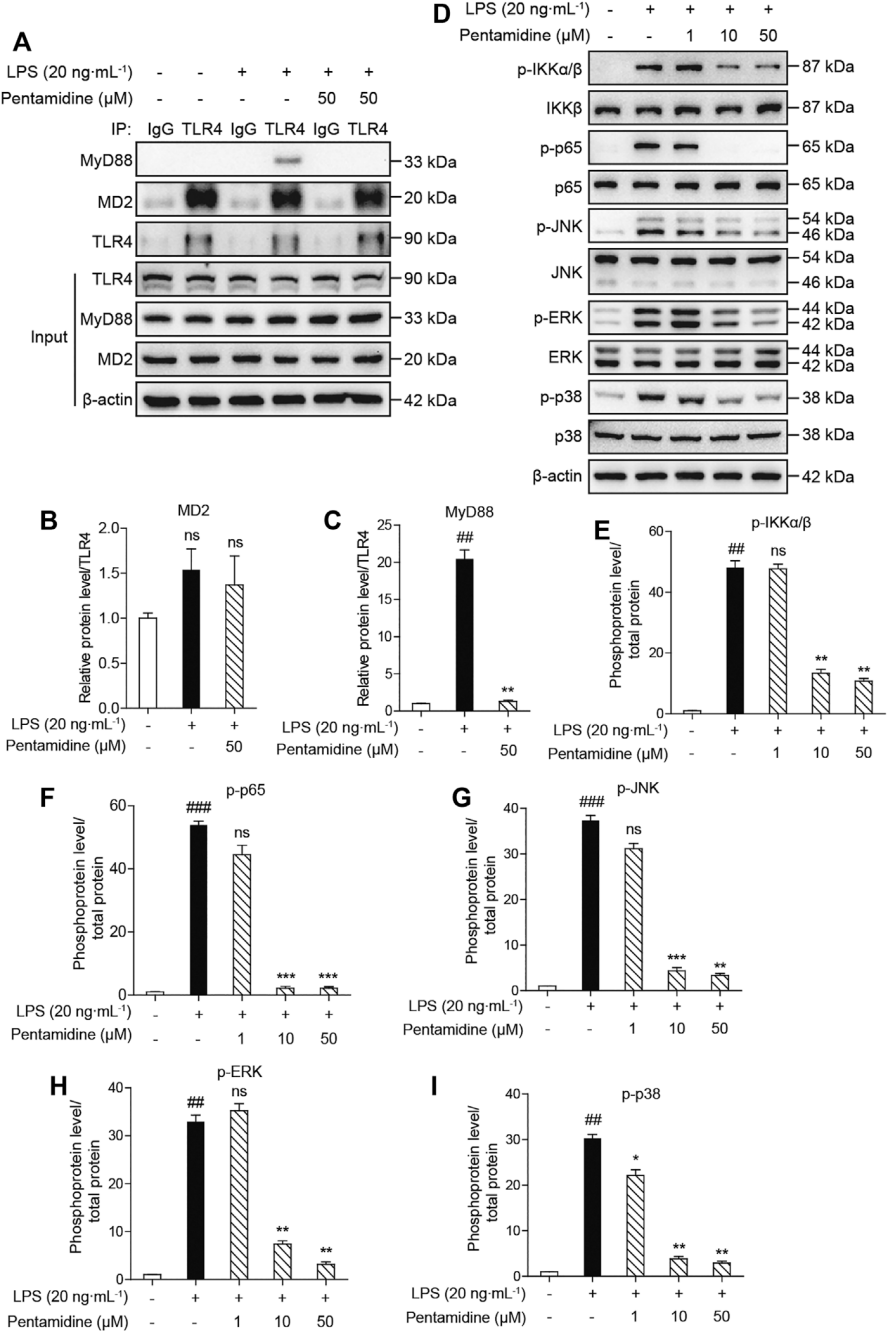
Cellular characterizations of pentamidine on TLR4 signaling. RAW 264.7 cells were administered with 20 ng ml^−1^ LPS and the indicated concentration of pentamidine for 1 h **(A–C)** Co-immunoprecipitation of anti-TLR4 antibody. MD2, TLR4, and MyD88 were detected by Western blotting. **(D–I)** The effect of pentamidine on LPS-induced phosphorylation of IKKβ, p65, and MAPKs. The protein level of β-actin was set as a reference. All experiments were performed thrice independently, and data were given as the mean ± S.E.M. ##, *p* < 0.01 versus the control; ###, *p* < 0.001 versus the control; **, *p* < 0.01 versus the LPS group; ***, *p* < 0.001 versus the LPS group; ns, not significant.

The activation of TLR4/MD2 can activate different signaling pathways, including the NF-κB (phosphorylation of IKKβ and p65) and MAPK (phosphorylation of p38, JNK, and ERK) signaling pathways. From the previous results obtained in cells, to investigate the effect of pentamidine on TLR4 signaling, immunoprecipitation and Western blotting were performed. As shown in Figures 7A–C, pentamidine inhibited the LPS-induced MyD88 recruitment of TLR4 and significantly suppressed the formation of the TLR4/MD2/MyD88 complex. LPS stimulation significantly increased the phosphorylation of IKKβ, p65, p38, JNK, and ERK, and pentamidine notably inhibited LPS-induced phosphorylation of these TLR4 signaling factors in a concentration-dependent manner (Figures 7D–I). All the results indicate that pentamidine inhibits the formation of the TLR4/MD2/MyD88 complex and the activation of NF-κB and MAPKs downstream of TLR4.

#### 2.2.8 Pentamidine Decreases the Mortality of Septic Mice and Alleviates Inflammation and Organ Damage

**FIGURE 8:**
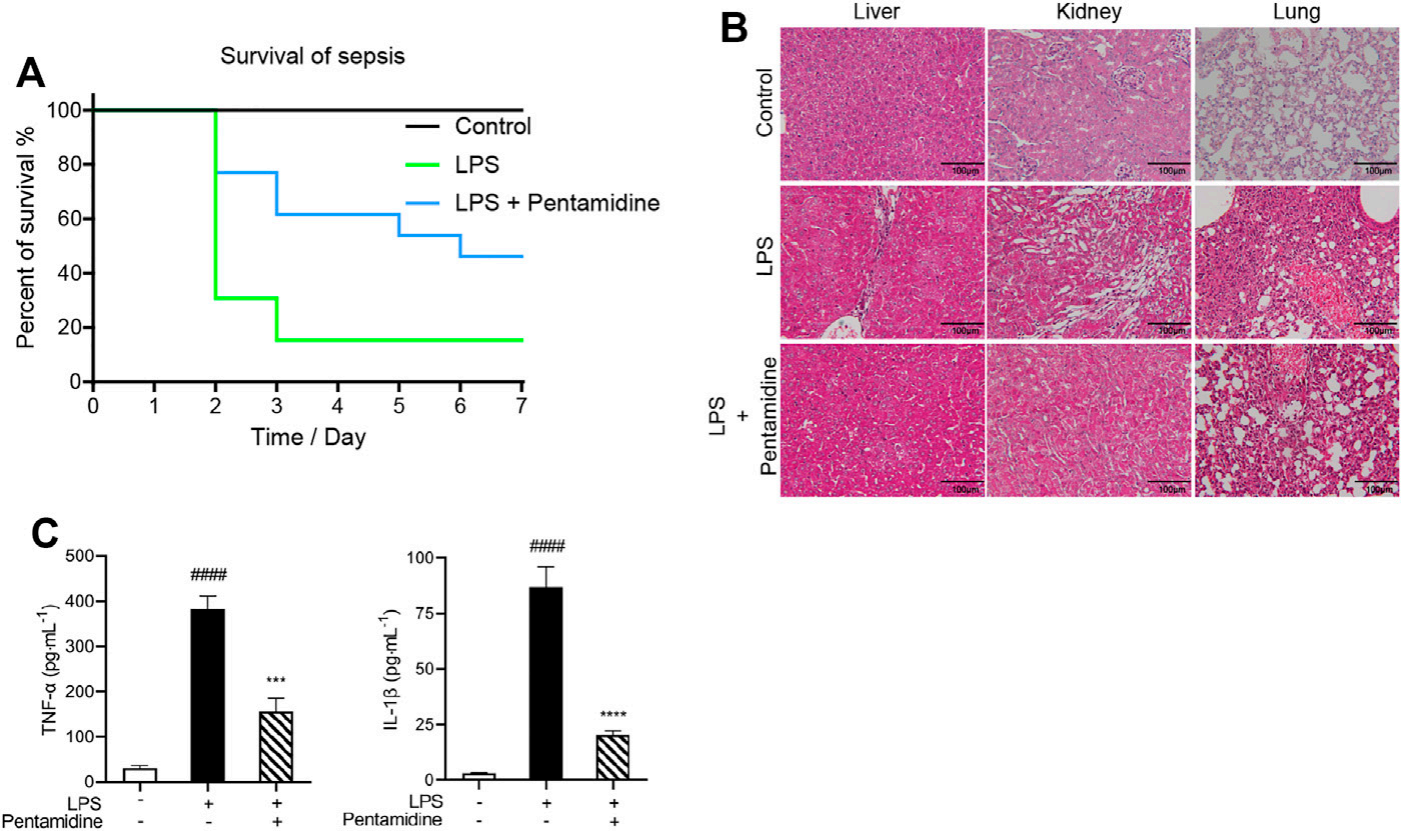
Pentamidine decreases the mortality of septic mice **(A)**, alleviates LPS-induced damages in liver, kidney, and lung **(B)**, and reduces LPS-induced systemic inflammation **(C)**. **(A)** Survival curves of mice under the indicated treatments with LPS and pentamidine. A single dose of LPS (25 mg/kg, *i.p.*; *n* = 12 per group) was administered to induce sepsis. Pentamidine (10 mg/kg., *i.p.*) was administered for 4 days. The mortality of mice was recorded for consecutive 7 days. **(B)** A single dose of LPS (25 mg/kg, *i.p.*; *n* = 6 per group) was administered to induce sepsis. A single dose of pentamidine (10 mg/kg, *i.p.*) was administered 30 min before LPS injection. After 24 h, mice were euthanized. The LPS induced damages in liver, kidney, and lung were checked by (H&E) staining. Three representative microscopic images were taken. Images were taken at ×400 magnification. **(C)** The pro-inflammatory factors TNF-α and IL-1β in sera from the mice treated as shown in **(B)** were measured by ELISA according to the manufacturer’s instructions. ####, *p* < 0.0001 versus the control; ***, *p* < 0.001 versus the LPS group; ****, *p* < 0.0001 versus the LPS group.

Pathogen infection leads to an acute cytokine storm, which is lethal to the body and causes enhanced death rates. To investigate whether pentamidine has protective effects *in vivo* from LPS stimulation, a sepsis animal model was performed. The survival rates of septic mice were recorded as shown in Figure 8A. Most of the mice in the LPS-treated group died in 7 days, while those injected with 10 mg/kg pentamidine survived notably longer. To investigate the protective ability of pentamidine in organ damage, the liver, kidney, and lung were detected by hematoxylin and eosin (H&E) staining (Figure 8B). Treatment with 10 mg/kg pentamidine alleviated cellular degeneration, interstitial edema, and disappearance of the alveolar septum, which indicates that pentamidine could effectively reduce organ damage in septic mice. To confirm the effective inhibition of LPS-induced *in vivo* inflammation by pentamidine, we also examined the levels of pro-inflammatory factors in sera at the same time before mice were anatomized. Compared to the LPS-treated group, TNF-α and IL-1β levels in sera were decreased in the group treated with pentamidine and LPS, respectively (Figure 8C). All of the data in the sepsis model reveal that pentamidine alleviates LPS-induced *in vivo* inflammation and decreases the death rate of sepsis, which implies that pentamidine has the potential to be repositioned as a therapeutic for treating sepsis.

### 2.3 Discussion

Among the innate immune systems, TLR4 plays a significant role in host defense (Janeway and Medzhitov, 2002). Herein, the FDA-approved drug pentamidine inhibited TLR4 signaling by directly targeting the adaptor protein MD2 of TLR4. Pentamidine inhibits the formation of the TLR4/MD2/MyD88 complex and suppresses the activation of NF-κB and MAPK downstream of TLR4. It is known that the activation of TLR4/MD2 induced by LPS leads to the overproduction of pro-inflammatory factors such as NO, TNF-α, and IL-1β (Liu et al., 2014). Pentamidine inhibits the TLR4 downstream pro-inflammatory factors NO, TNF-α, and IL-1β with activities of ∼μM, which is in the range of plasma concentrations (0.42–13.42 μM) of pentamidine during treatment for Trypanosoma gambiense infections (Bronner et al., 1991). In addition to treating protozoal-caused infections (Sands et al., 1985), pentamidine has been found to exert an anti-inflammatory effect (Corsini et al., 1992a). Studies have also reported that pentamidine could bind to lipid A of LPS (David et al., 1994). However, the precise molecular mechanisms underlying the anti-inflammatory effect of pentamidine are not fully understood. This work demonstrates MD2 as a direct binding target of pentamidine and reports for the first time that pentamidine is a TLR4 antagonist, which at least in part accounts for its anti-inflammatory activity. Taken together, these findings suggest that the TLR4 antagonism of pentamidine may be related to both binding to lipid A and targeting MD2. The bioisosteric replacement of the methylene group at the center 13′ site of pentamidine by the ether oxygen group significantly decreased its interactions with MD2 and abolished its TLR4 antagonist activity. Penta-2 is less hydrophobic than pentamidine, thus making it unstable in the hydrophobic cavity MD2 and attenuating its TLR4 antagonistic activity.

Sepsis is a dysregulated host response to an infection and is frequently related to acute organ dysfunction, and even worse, to death. This syndrome needs urgent treatment to save patients from severe cytokine storms. Therefore, the rapid decrease in pro-inflammatory factors is of great importance (Cecconi et al., 2018). However, the most promising small-molecule inhibitor of TLR4, TAK242, failed in a Phase Ⅲ study in a clinical trial in patients with sepsis or respiratory failure (Rice et al., 2010). Repositioning of existing clinical drugs as TLR4 modulators could provide a feasible solution (Wang et al., 2013; Peng et al., 2019; Zhang et al., 2021). As an FDA-approved drug, the toxicity of pentamidine has been extensively studied. The LD_50_ of pentamidine on mice is 63 mg/kg (McEvoy, 2007), which is much higher than the dosage (10 mg/kg) used in this study. What is more, consistent treatment with 25 mg/kg/day pentamidine isethionate on C57BL/6 mice for 22 days showed no obvious sign of toxicity, and the mortality was lower than 1% (National Toxicology Program, 1989). In this study, pentamidine alleviated LPS-induced *in vivo* inflammation and decreased the death rate of sepsis, which provides strong initial support for pentamidine repositioning as a therapeutic for sepsis.

In summary, this study provides direct evidence that pentamidine targets MD2, which inhibits the formation of the TLR4/MD2/MyD88 complex and suppresses the activation of TLR4 downstream of NF-κB and MAPKs, therefore blocking LPS-induced TLR4 signaling downstream of pro-inflammatory factors. Furthermore, pentamidine alleviates LPS-induced *in vivo* inflammation and decreases the death rate of sepsis. The results indicate that pentamidine could be repurposed as a potential therapeutic intervention for sepsis. Further understanding of the structure-activity relationship of pentamidine will facilitate the development of a new agent with better pharmacological activity. Meanwhile, the interactions among LPS, pentamidine, and MD2 are complicated pairwise interactions, which need to be further studied and elucidated.

## Data Availability

The original contributions presented in the study are included in the article/Supplementary Material, further inquiries can be directed to the corresponding authors.
