# Supplementary figures and images for: Pentamidine Alleviates Inflammation and Lipopolysaccharide-Induced Sepsis by Inhibiting TLR4 Activation via Targeting MD2
Source: Front Pharmacol. 2022 Feb 23;13:835081. doi: 10.3389/fphar.2022.835081 (PMC8905599; doi:10.3389/fphar.2022.835081)

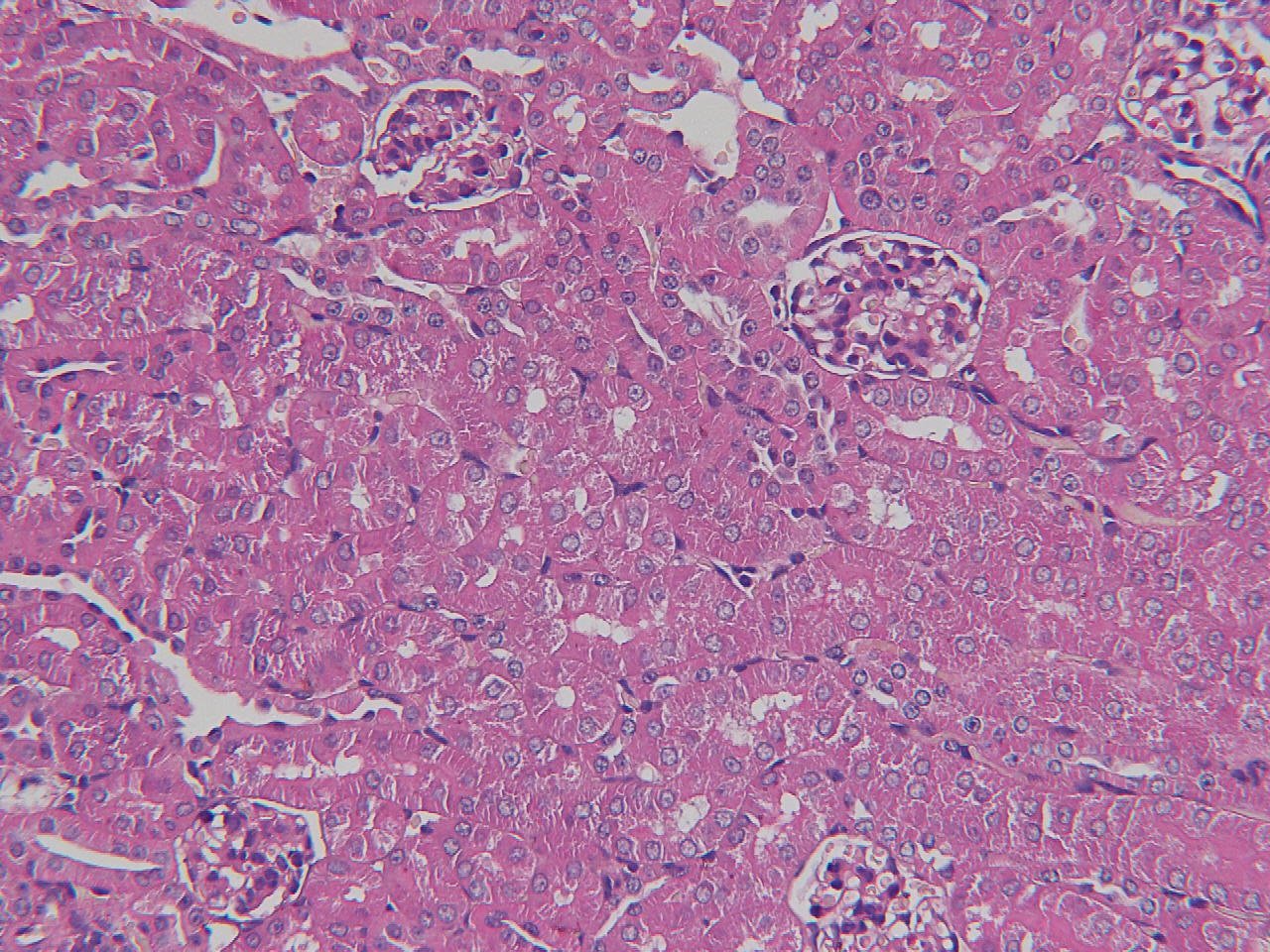

Supplement: Supplementary file 2 [file DataSheet9.ZIP › Figure 8/H&E/Control/kidney.jpg]

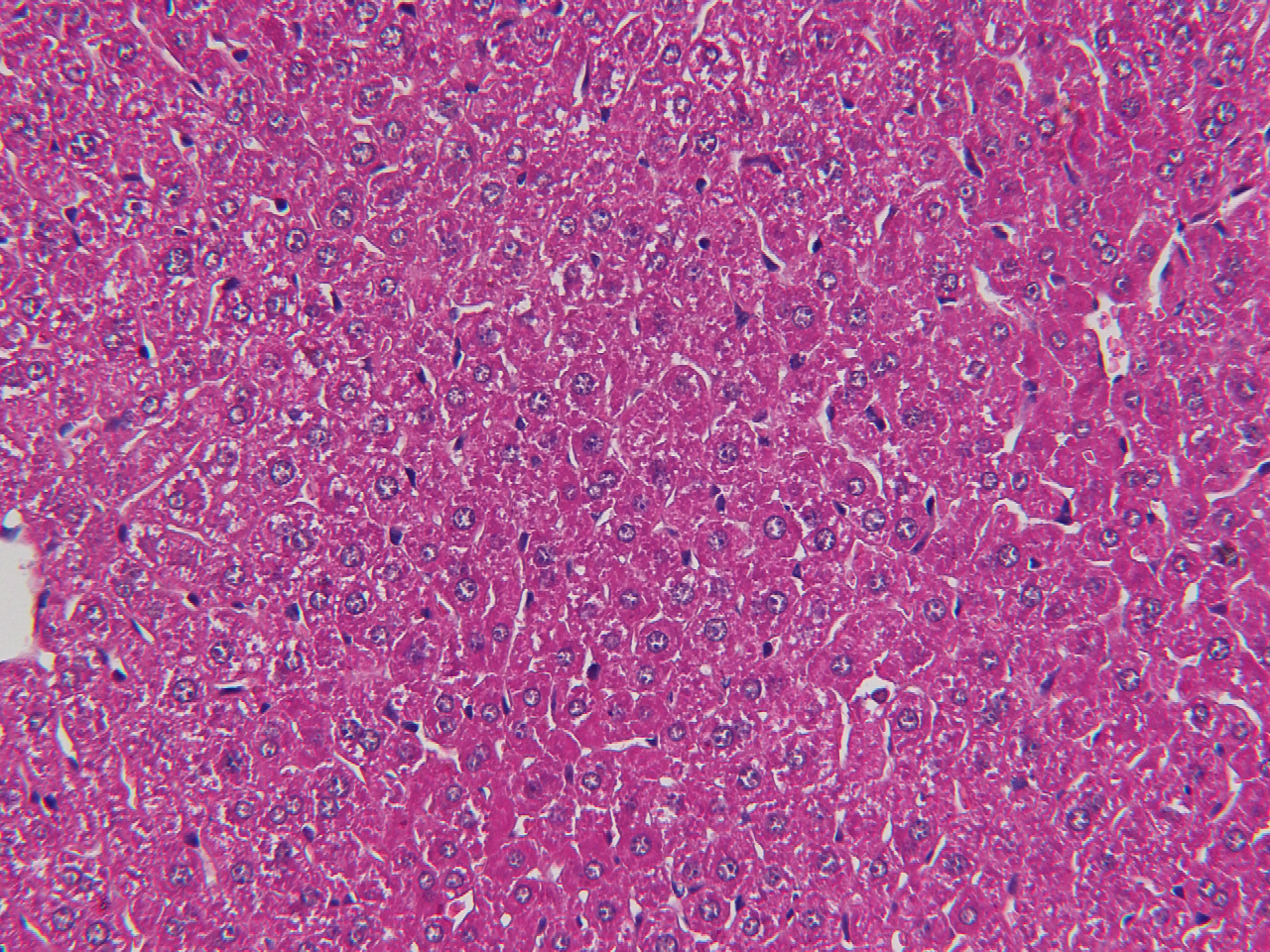

Supplement: Supplementary file 2 [file DataSheet9.ZIP › Figure 8/H&E/Control/liver.jpg]

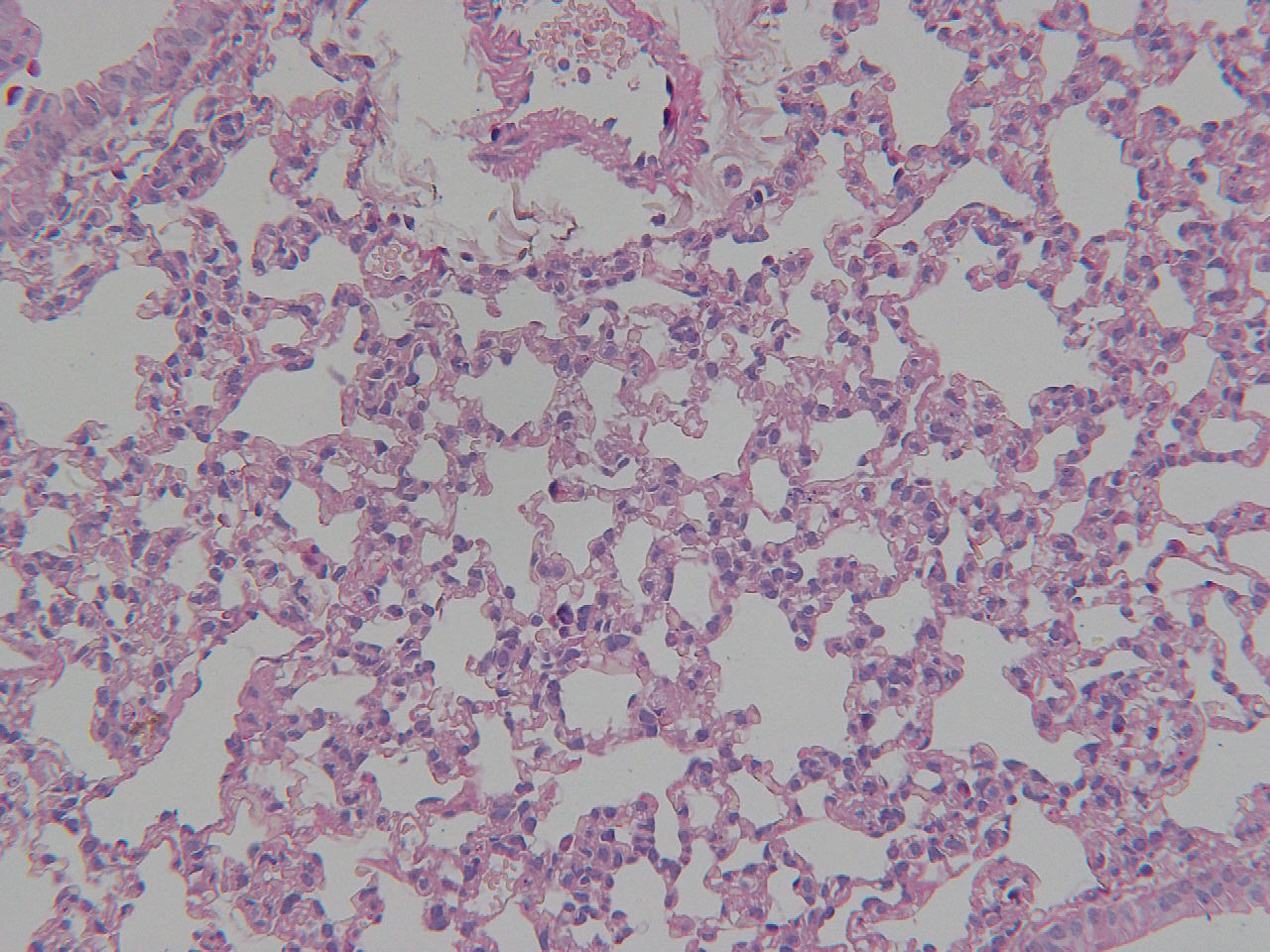

Supplement: Supplementary file 2 [file DataSheet9.ZIP › Figure 8/H&E/Control/lung.jpg]

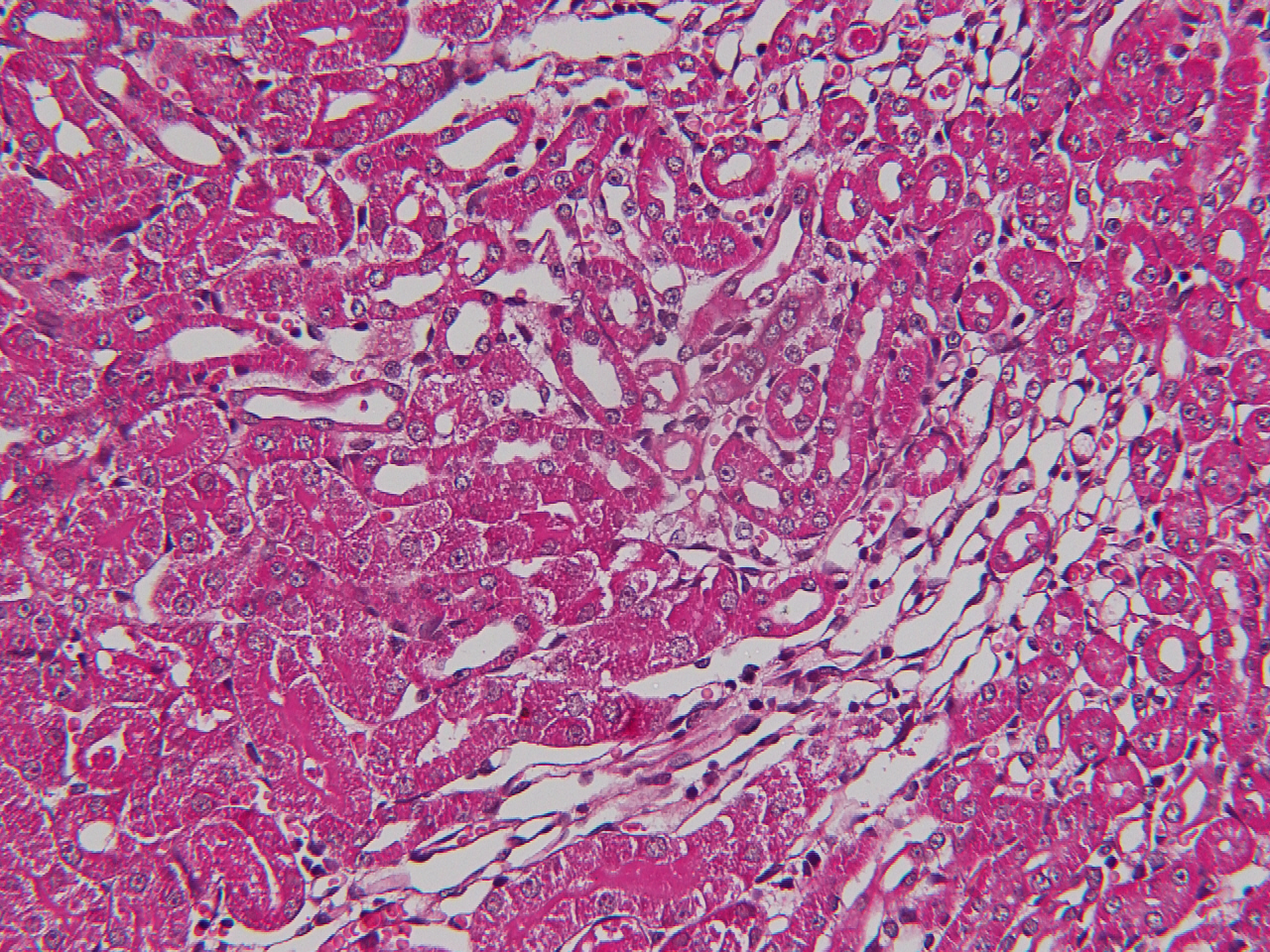

Supplement: Supplementary file 2 [file DataSheet9.ZIP › Figure 8/H&E/LPS/kidney.jpg]

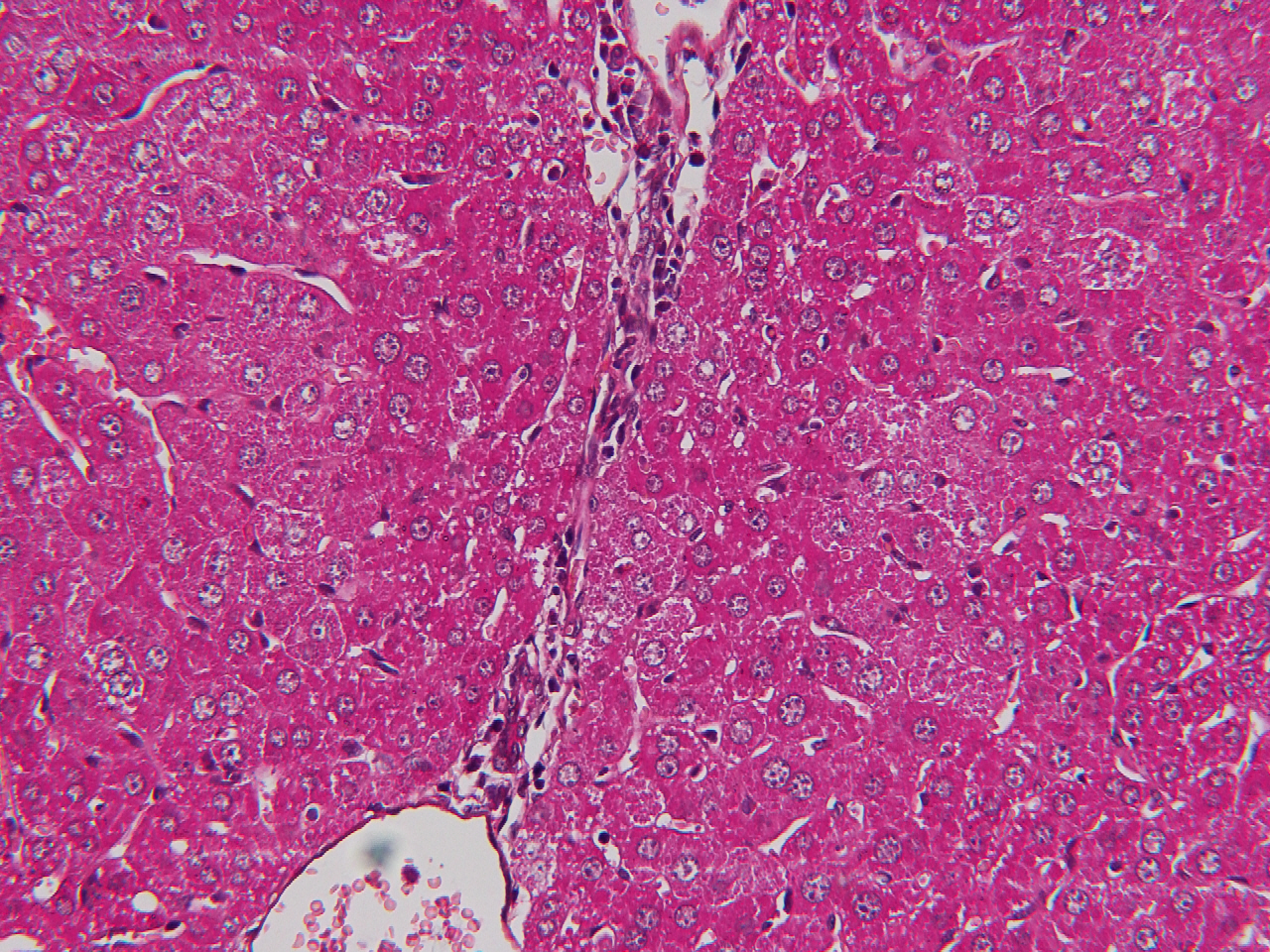

Supplement: Supplementary file 2 [file DataSheet9.ZIP › Figure 8/H&E/LPS/liver.jpg]

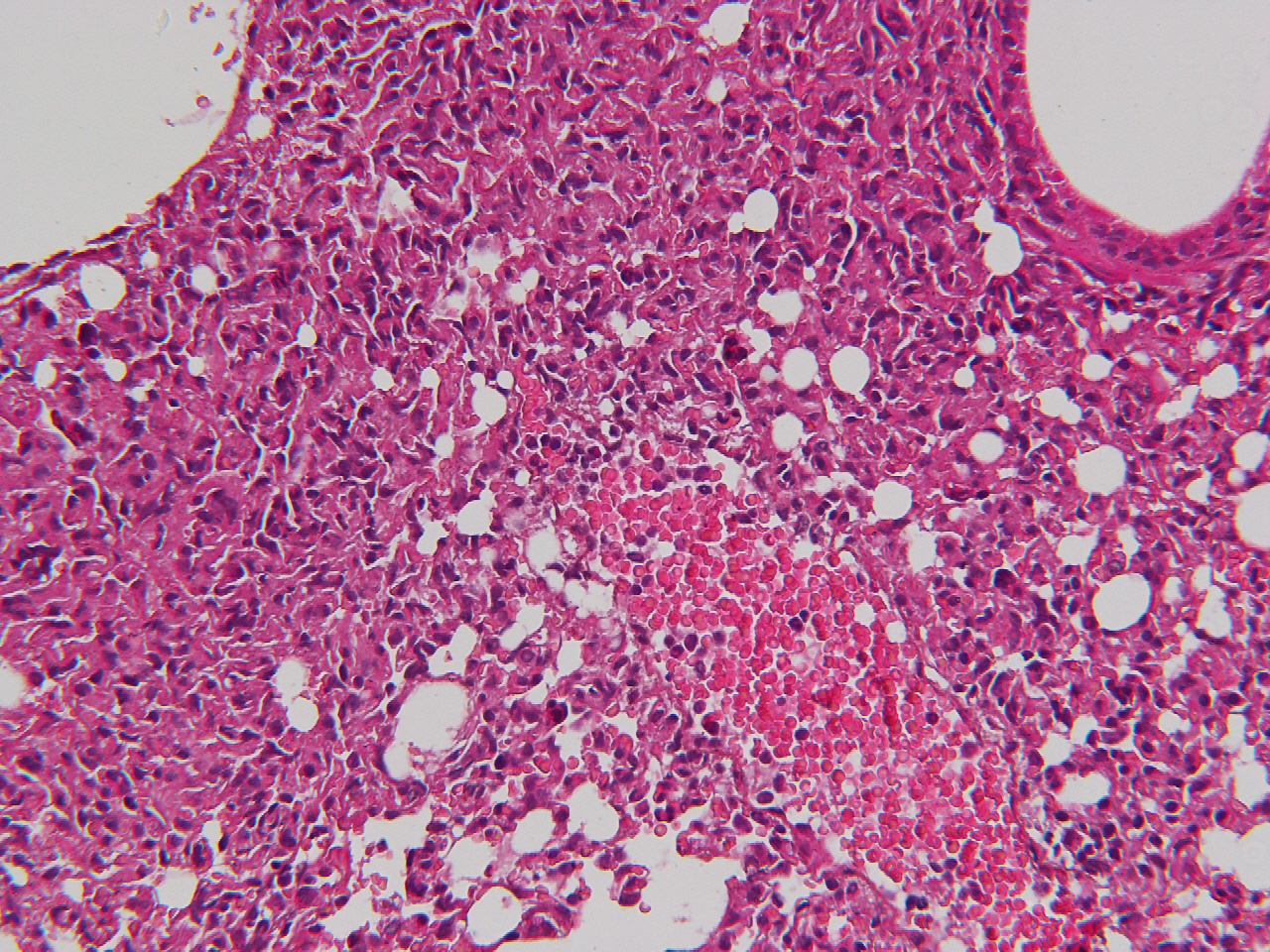

Supplement: Supplementary file 2 [file DataSheet9.ZIP › Figure 8/H&E/LPS/lung.jpg]

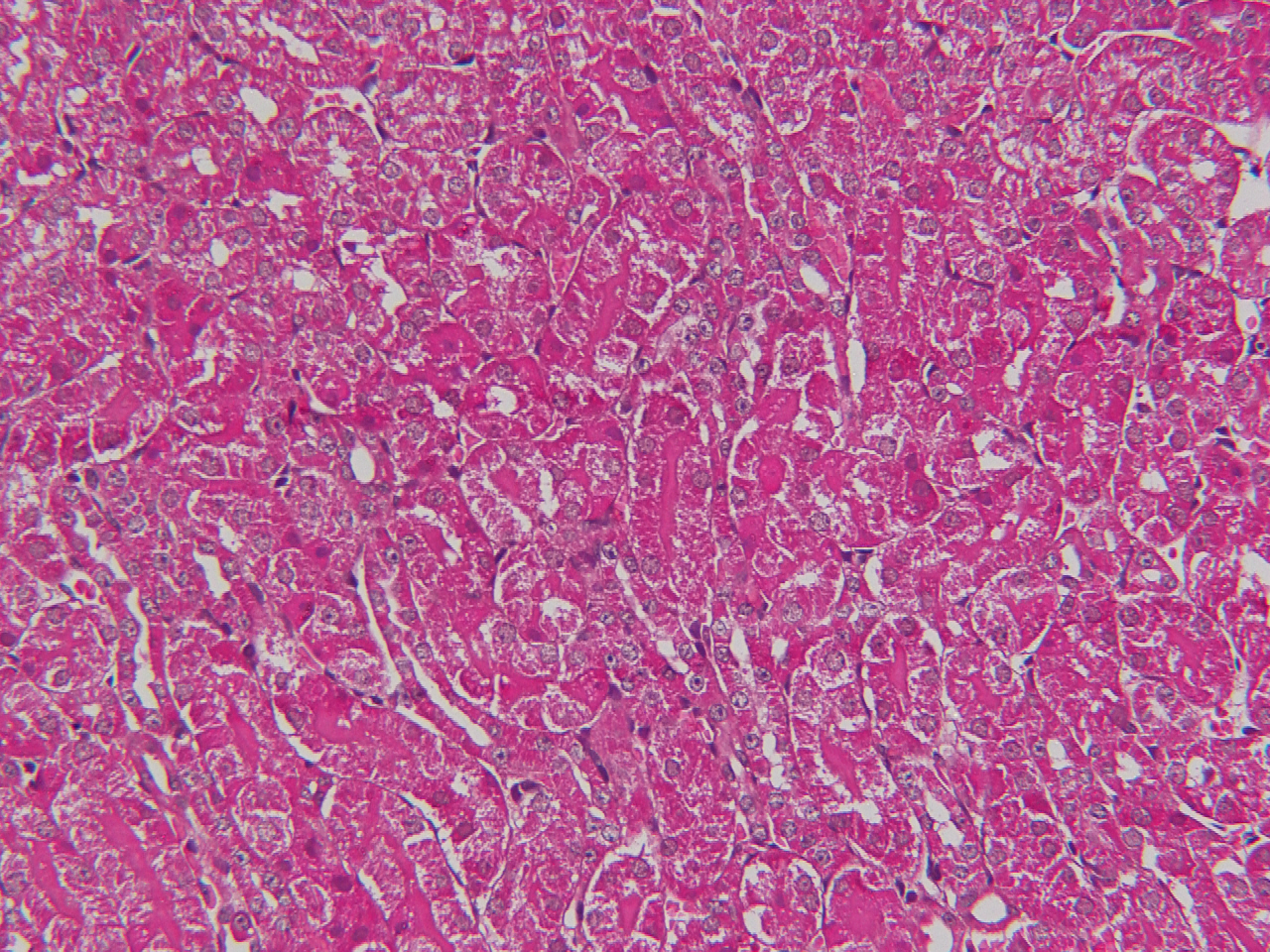

Supplement: Supplementary file 2 [file DataSheet9.ZIP › Figure 8/H&E/LPS+Pentamidine/kidney.jpg]

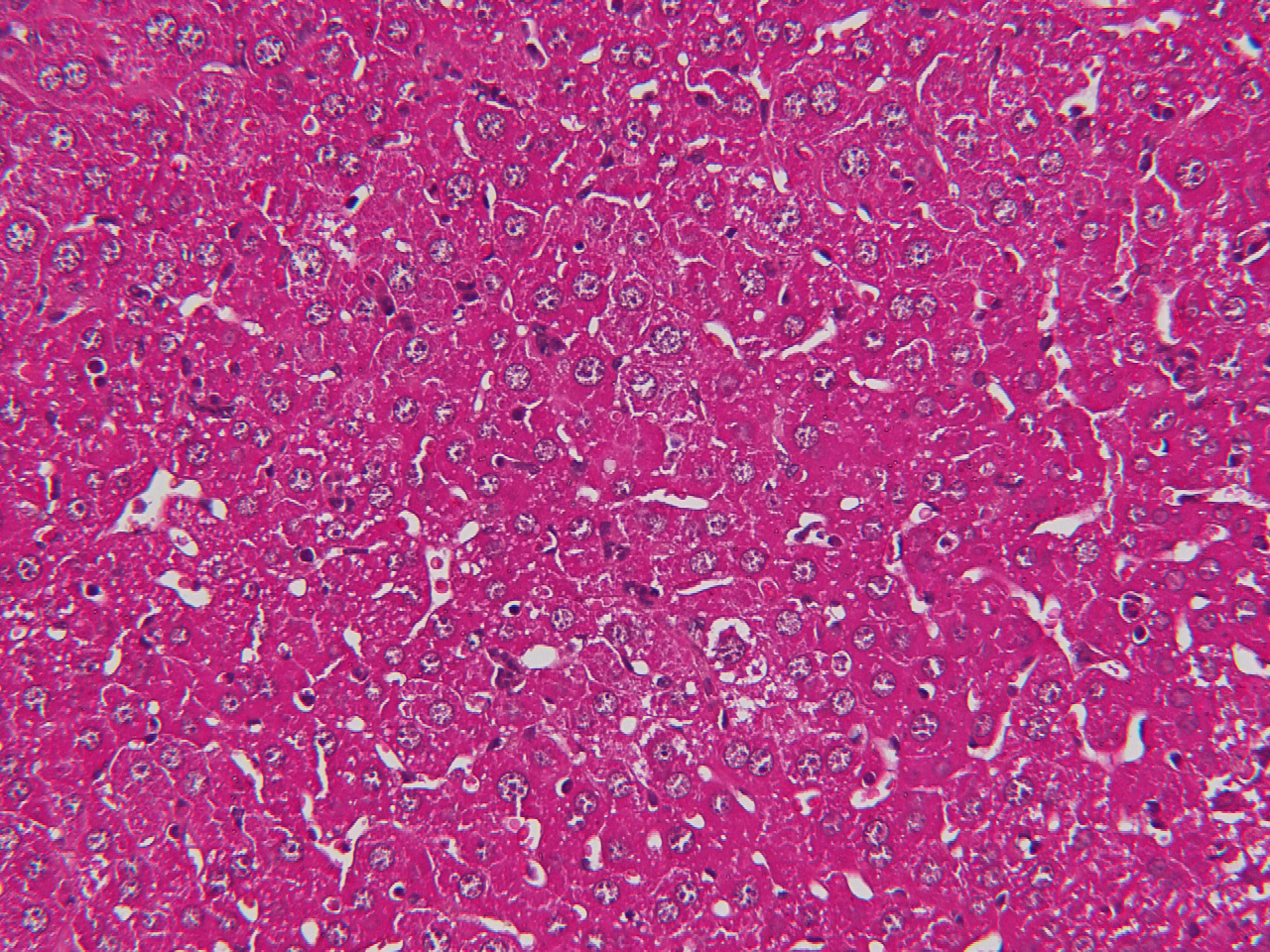

Supplement: Supplementary file 2 [file DataSheet9.ZIP › Figure 8/H&E/LPS+Pentamidine/liver.jpg]

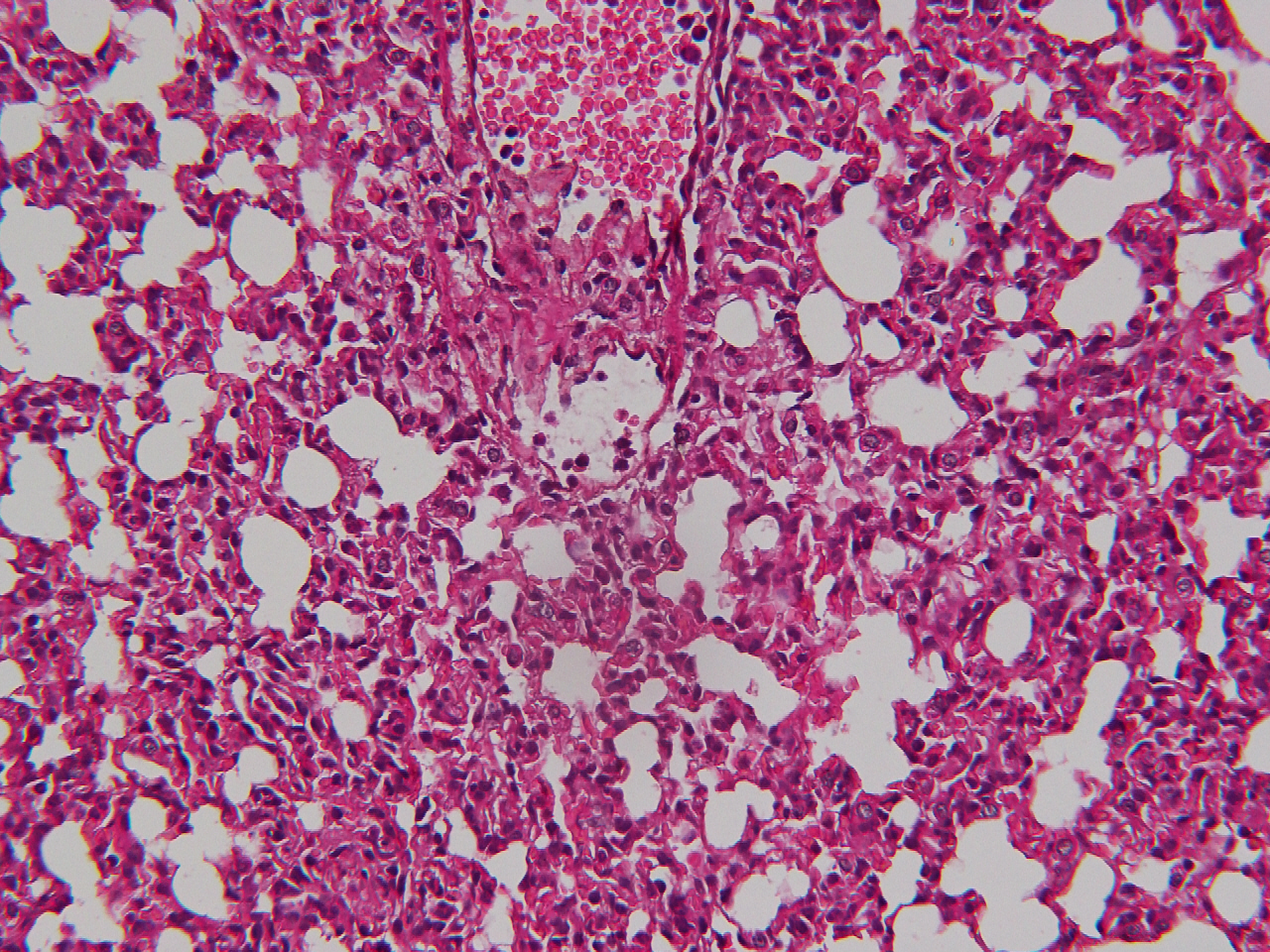

Supplement: Supplementary file 2 [file DataSheet9.ZIP › Figure 8/H&E/LPS+Pentamidine/lung.jpg]

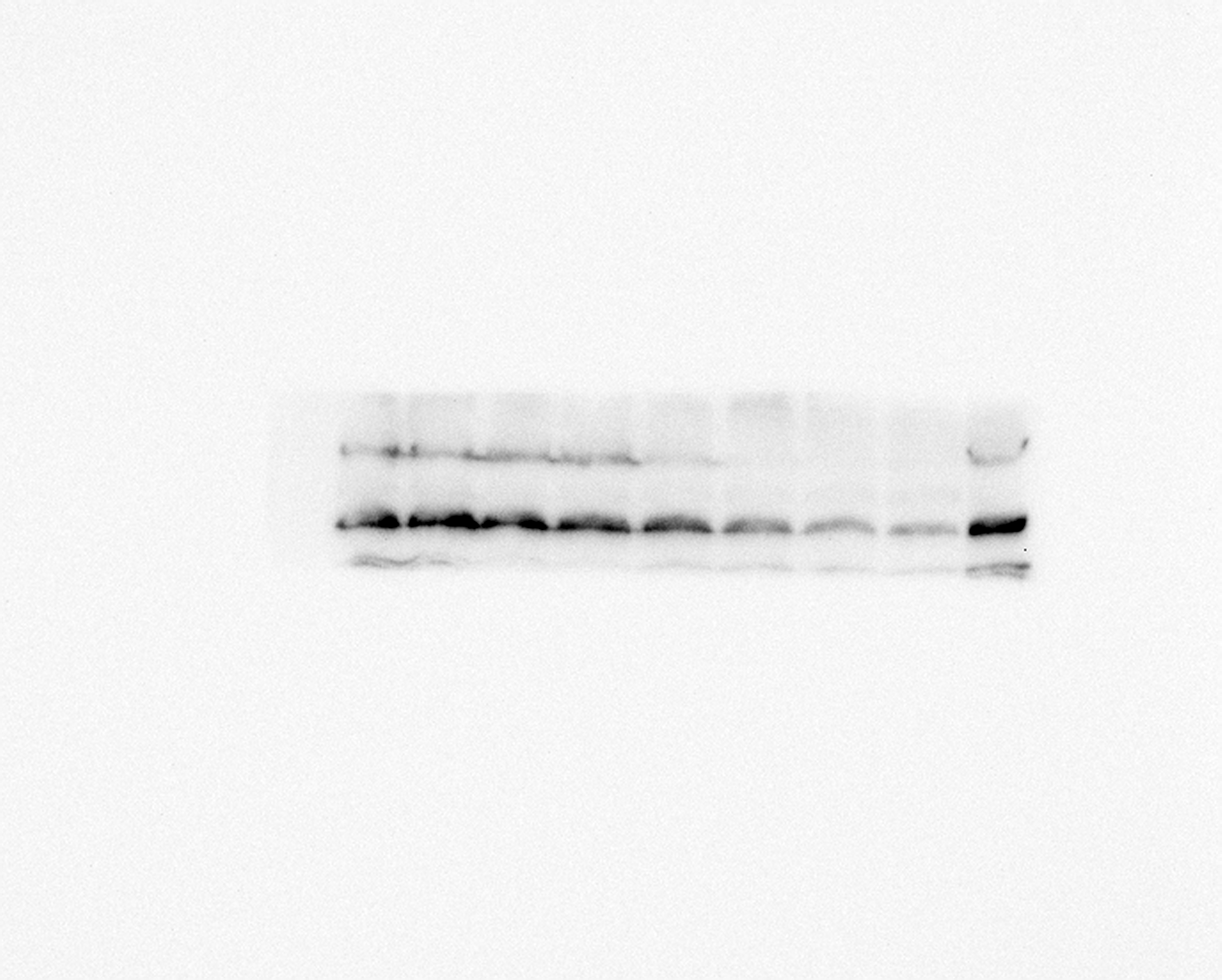

Supplement: Supplementary file 3 [file DataSheet2.zip › figure 1/Thermal Shift/1st/control.tif]

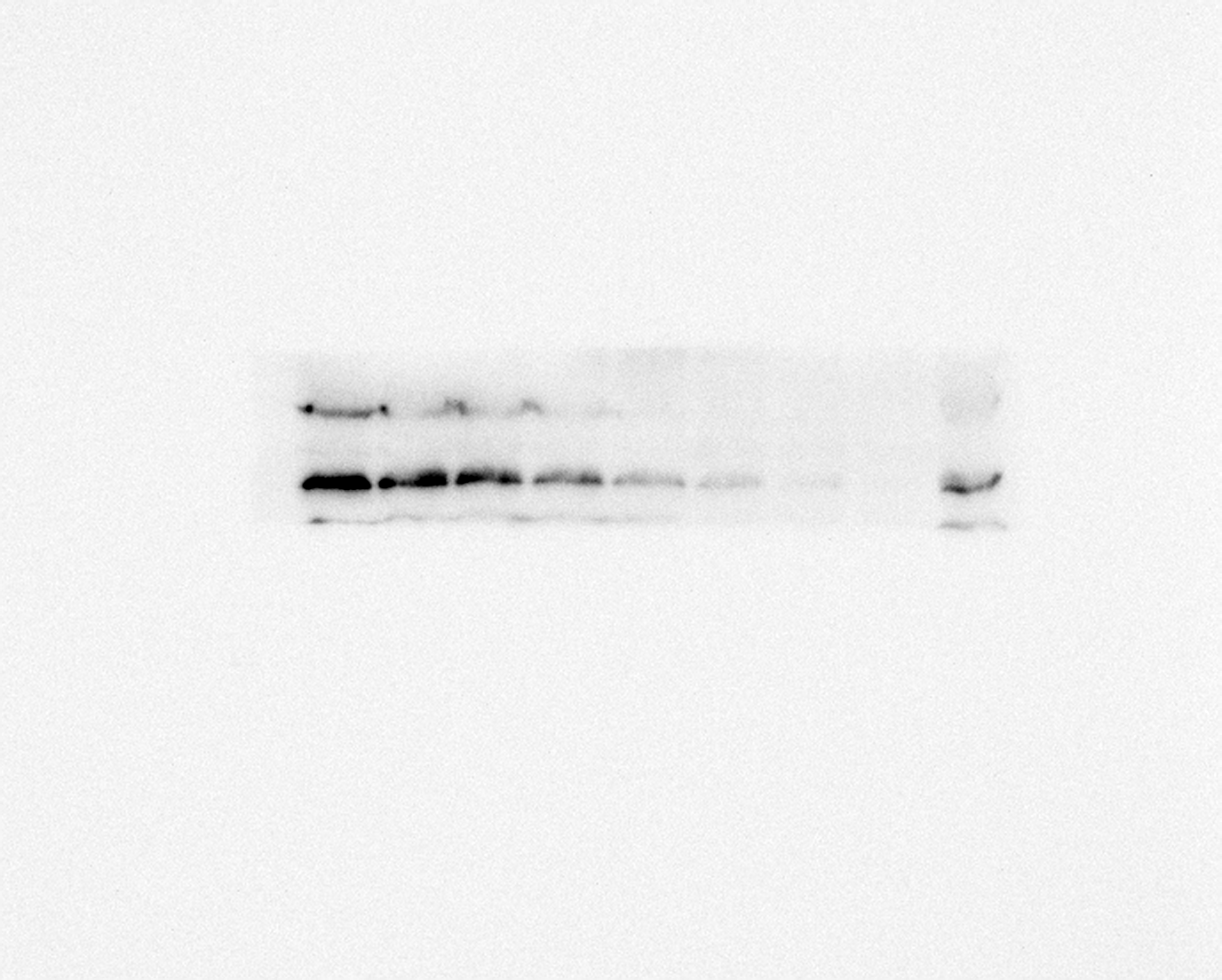

Supplement: Supplementary file 3 [file DataSheet2.zip › figure 1/Thermal Shift/1st/pentamidine.tif]

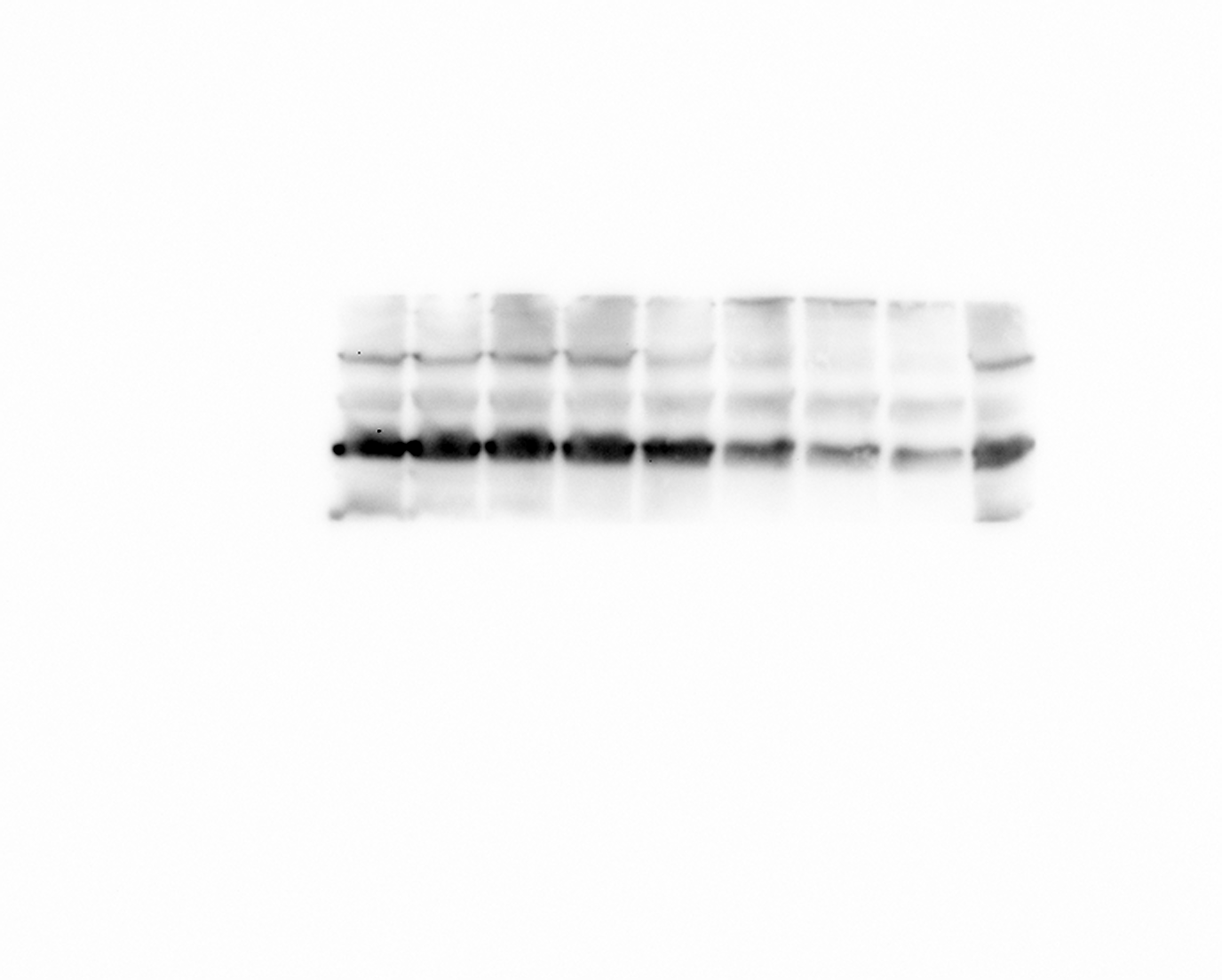

Supplement: Supplementary file 3 [file DataSheet2.zip › figure 1/Thermal Shift/2nd/120s-control.tif]

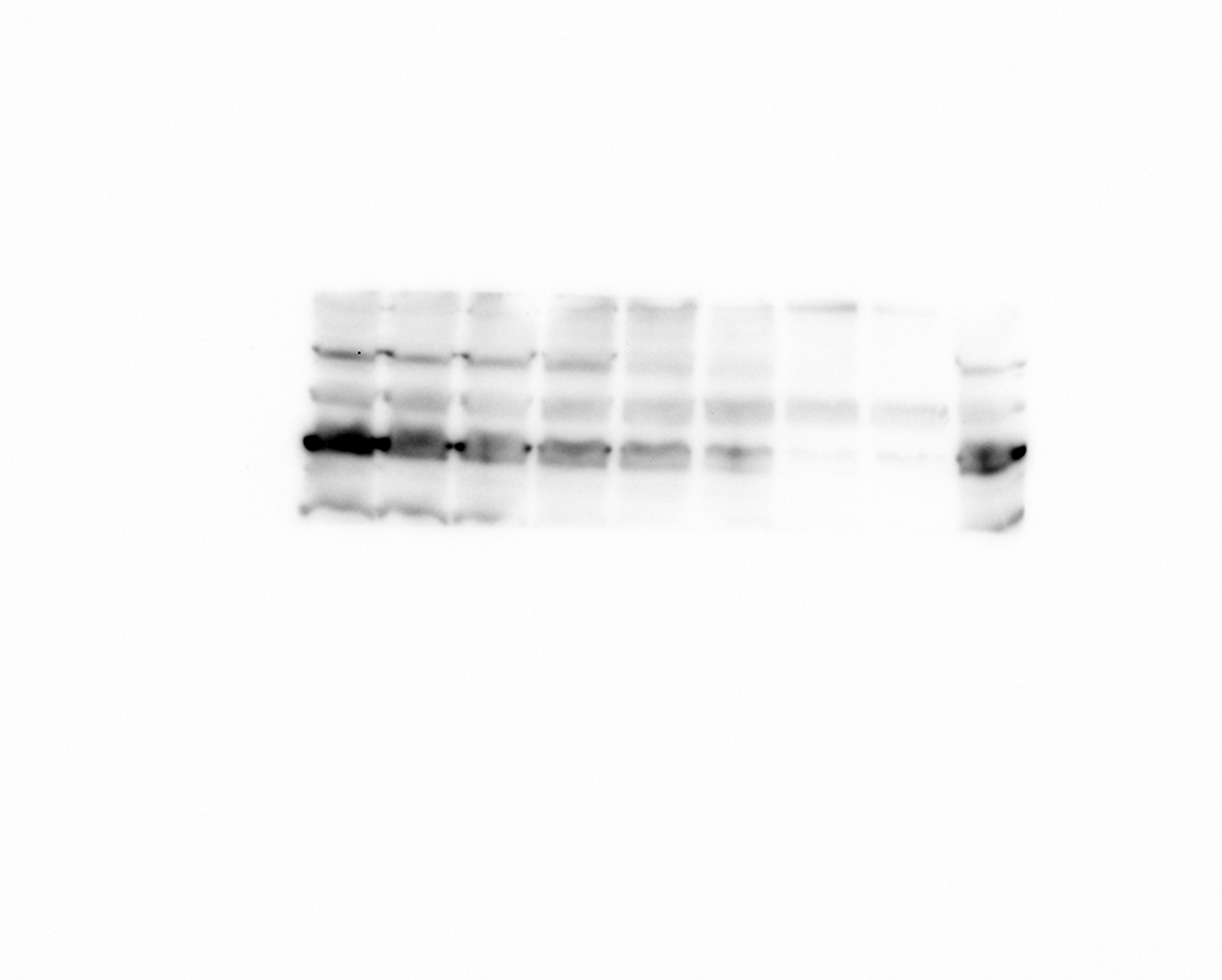

Supplement: Supplementary file 3 [file DataSheet2.zip › figure 1/Thermal Shift/2nd/120s-pentamidine.tif]

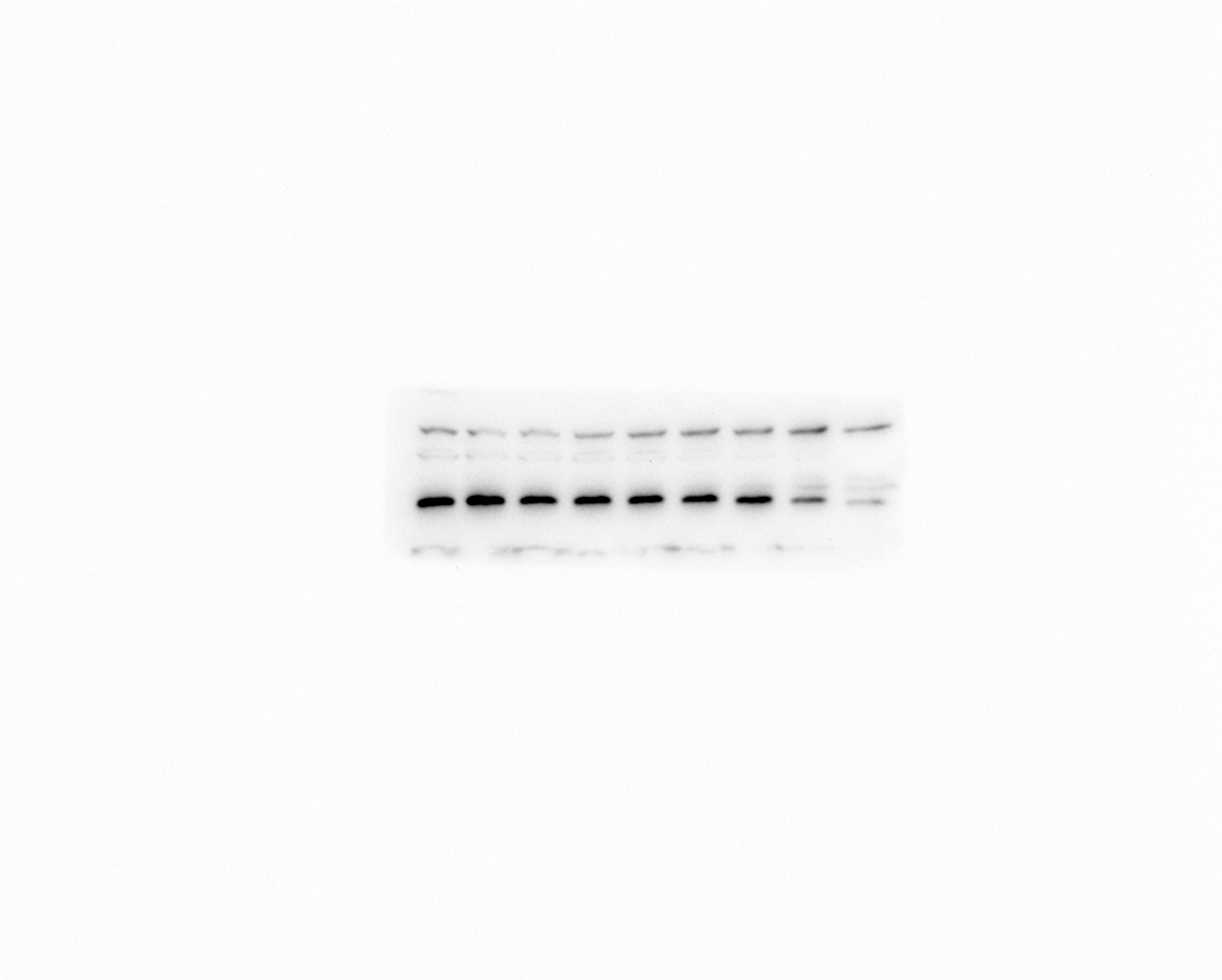

Supplement: Supplementary file 3 [file DataSheet2.zip › figure 1/Thermal Shift/3rd/control.tif]

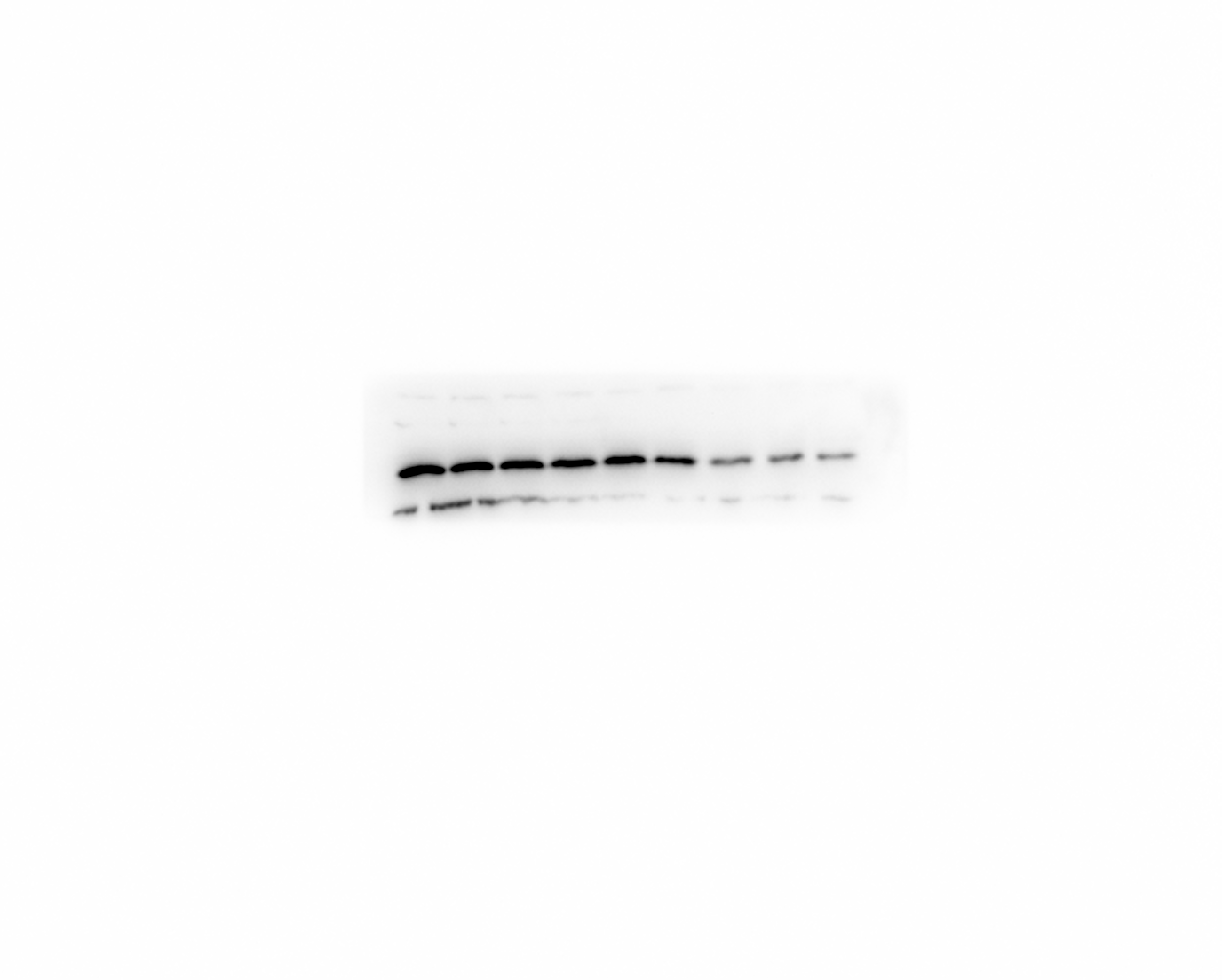

Supplement: Supplementary file 3 [file DataSheet2.zip › figure 1/Thermal Shift/3rd/pentamidine.tif]

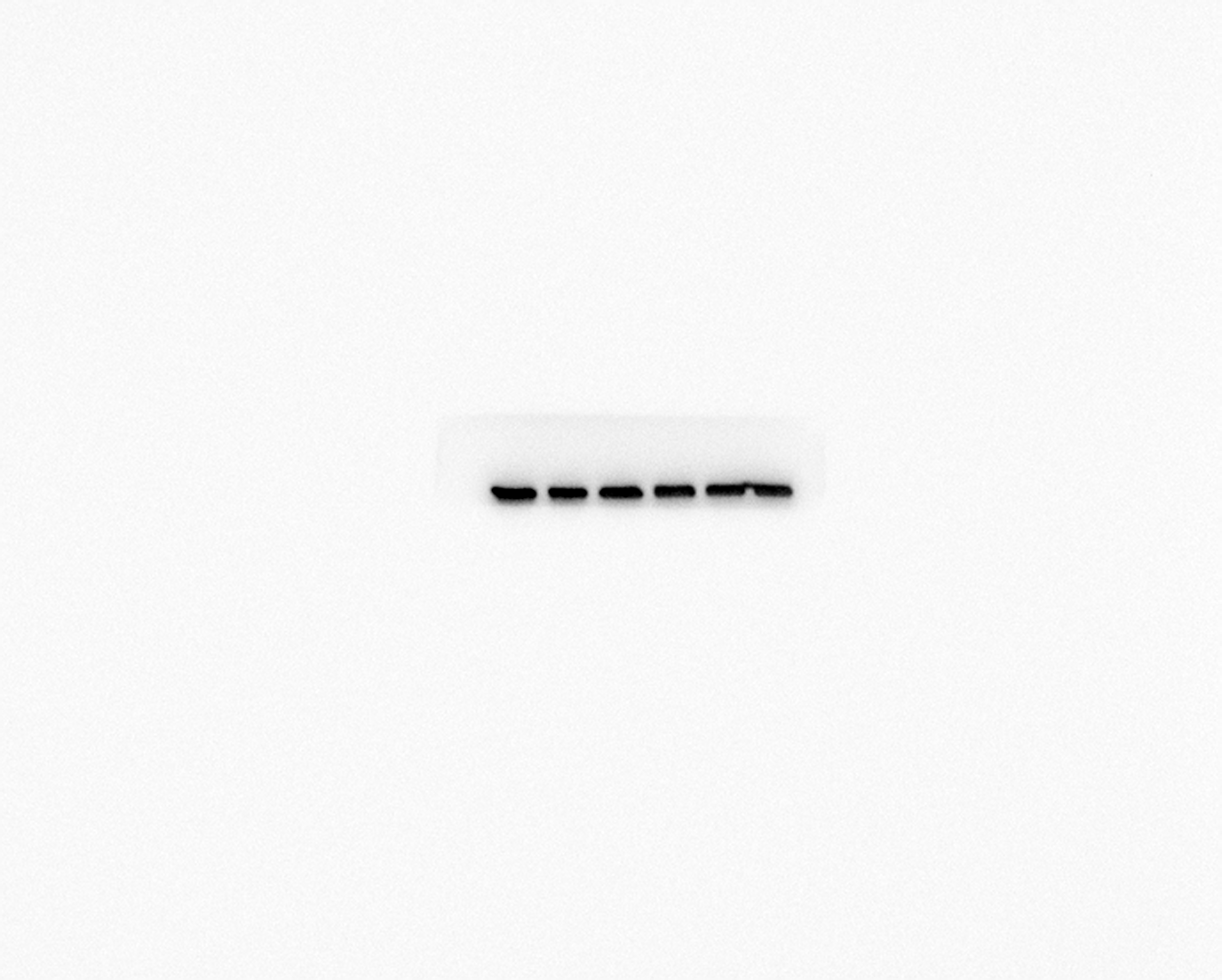

Supplement: Supplementary file 5 [file DataSheet7.ZIP › Co-IP/Input/1/actin/2.tif]

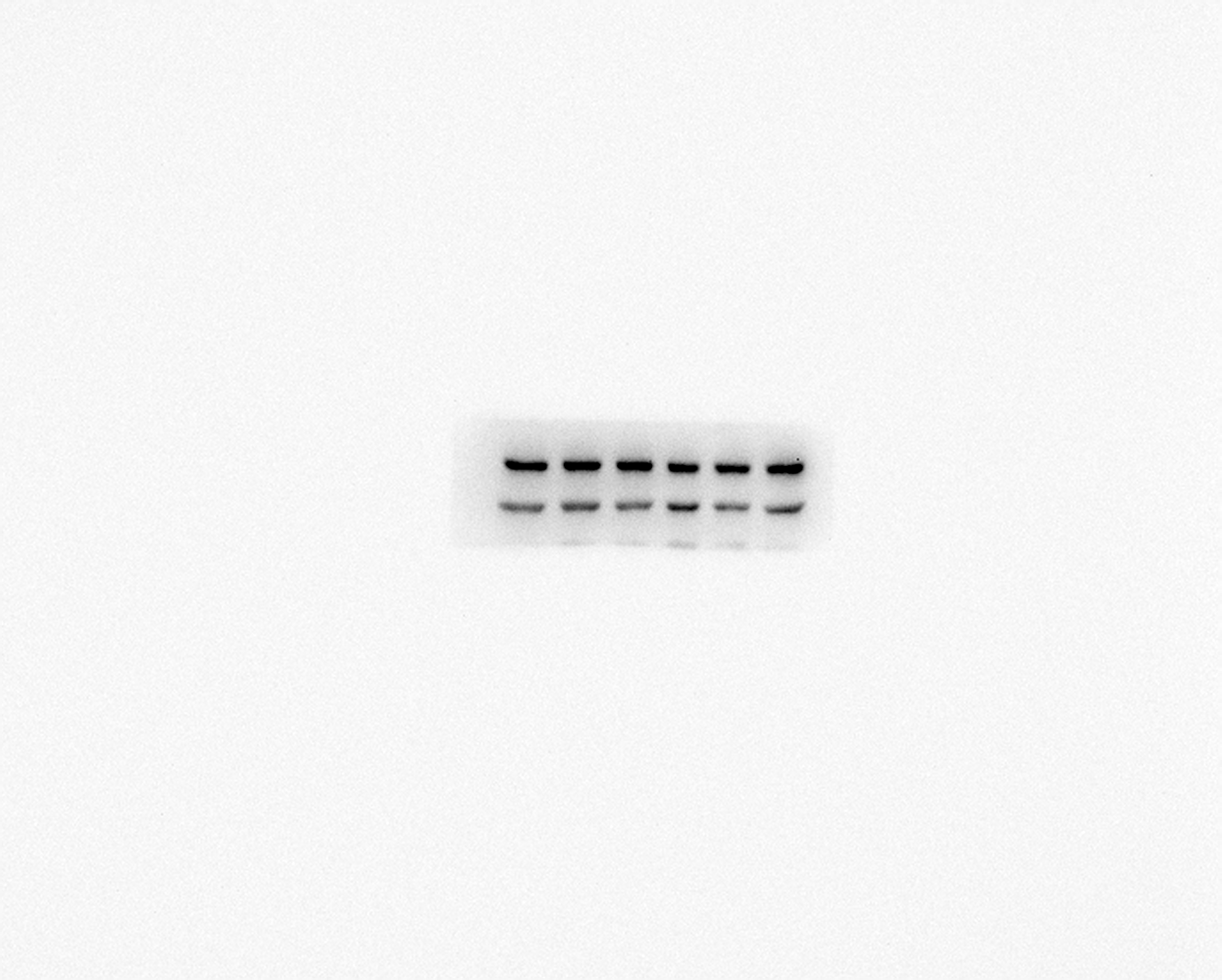

Supplement: Supplementary file 5 [file DataSheet7.ZIP › Co-IP/Input/1/MD2/1.tif]

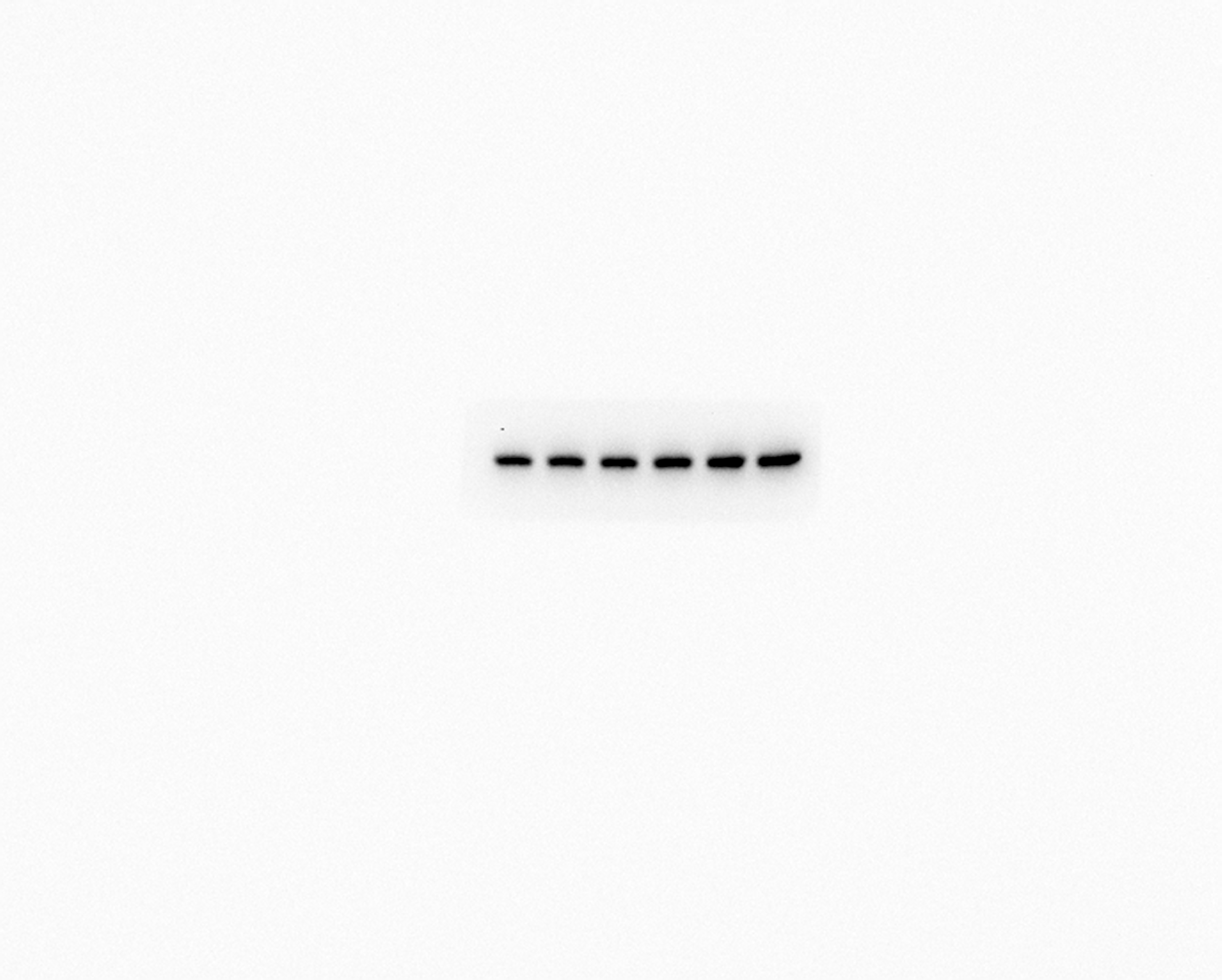

Supplement: Supplementary file 5 [file DataSheet7.ZIP › Co-IP/Input/1/MyD88/1.tif]

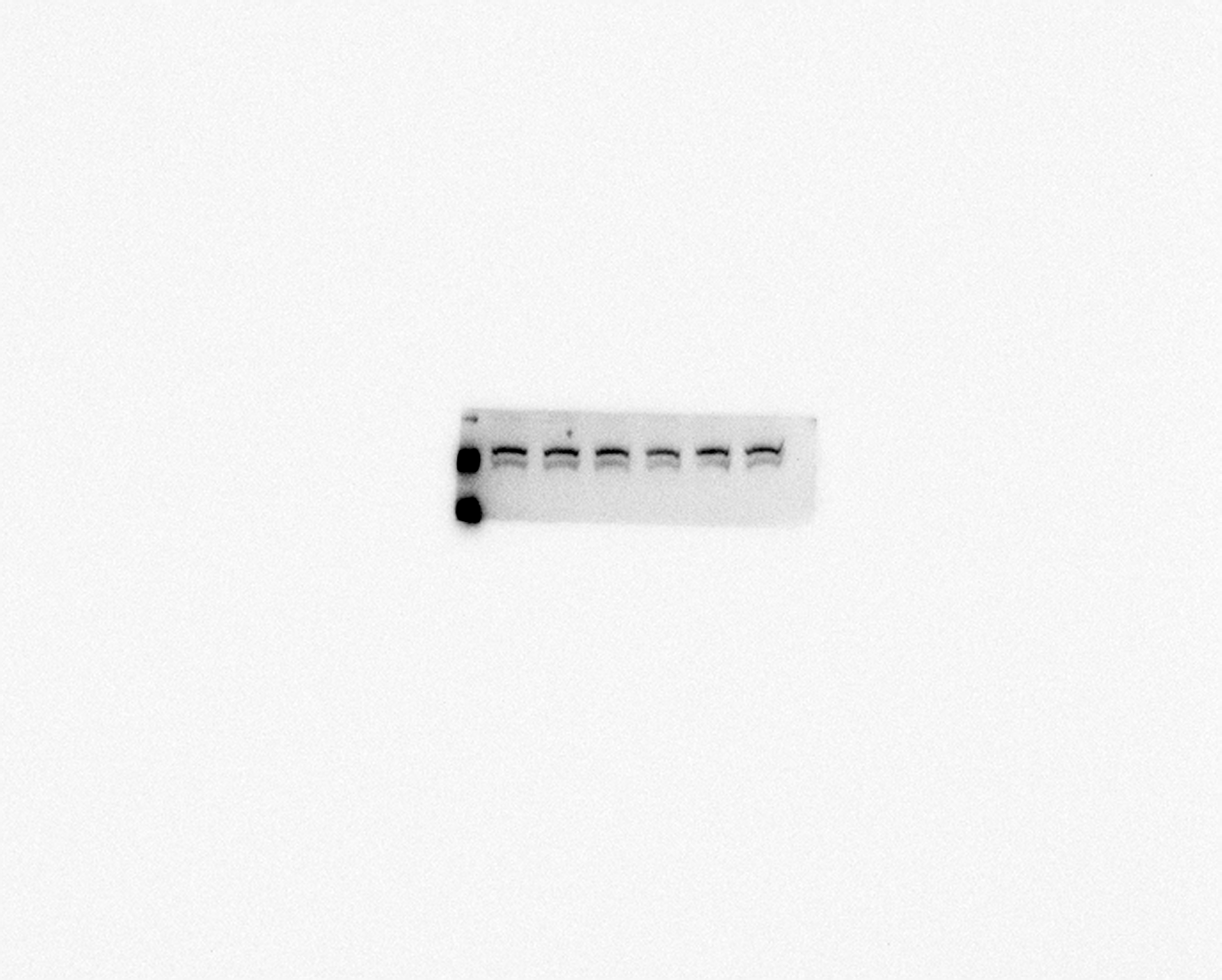

Supplement: Supplementary file 5 [file DataSheet7.ZIP › Co-IP/Input/1/TLR4/2.tif]

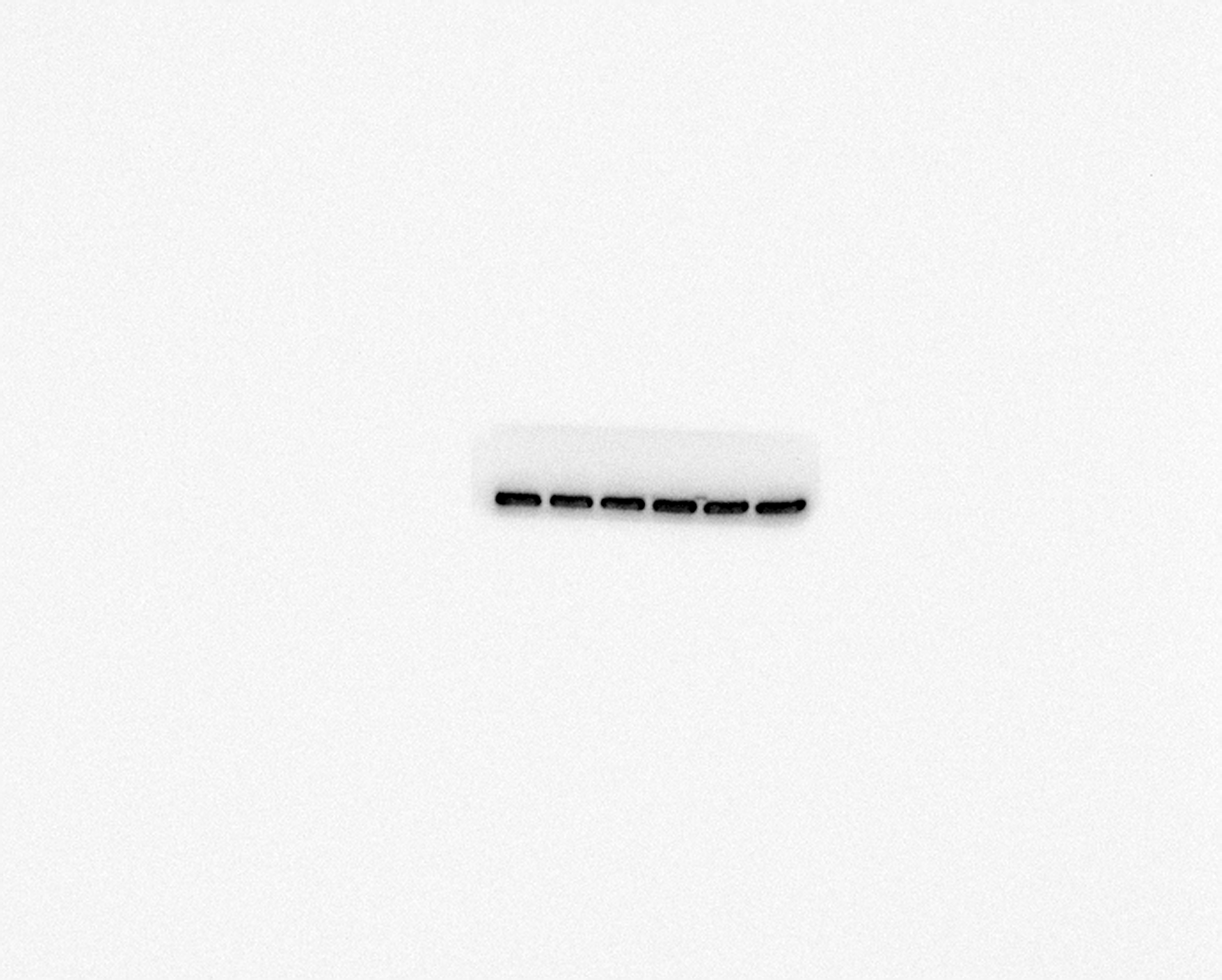

Supplement: Supplementary file 5 [file DataSheet7.ZIP › Co-IP/Input/2/actin/2.tif]

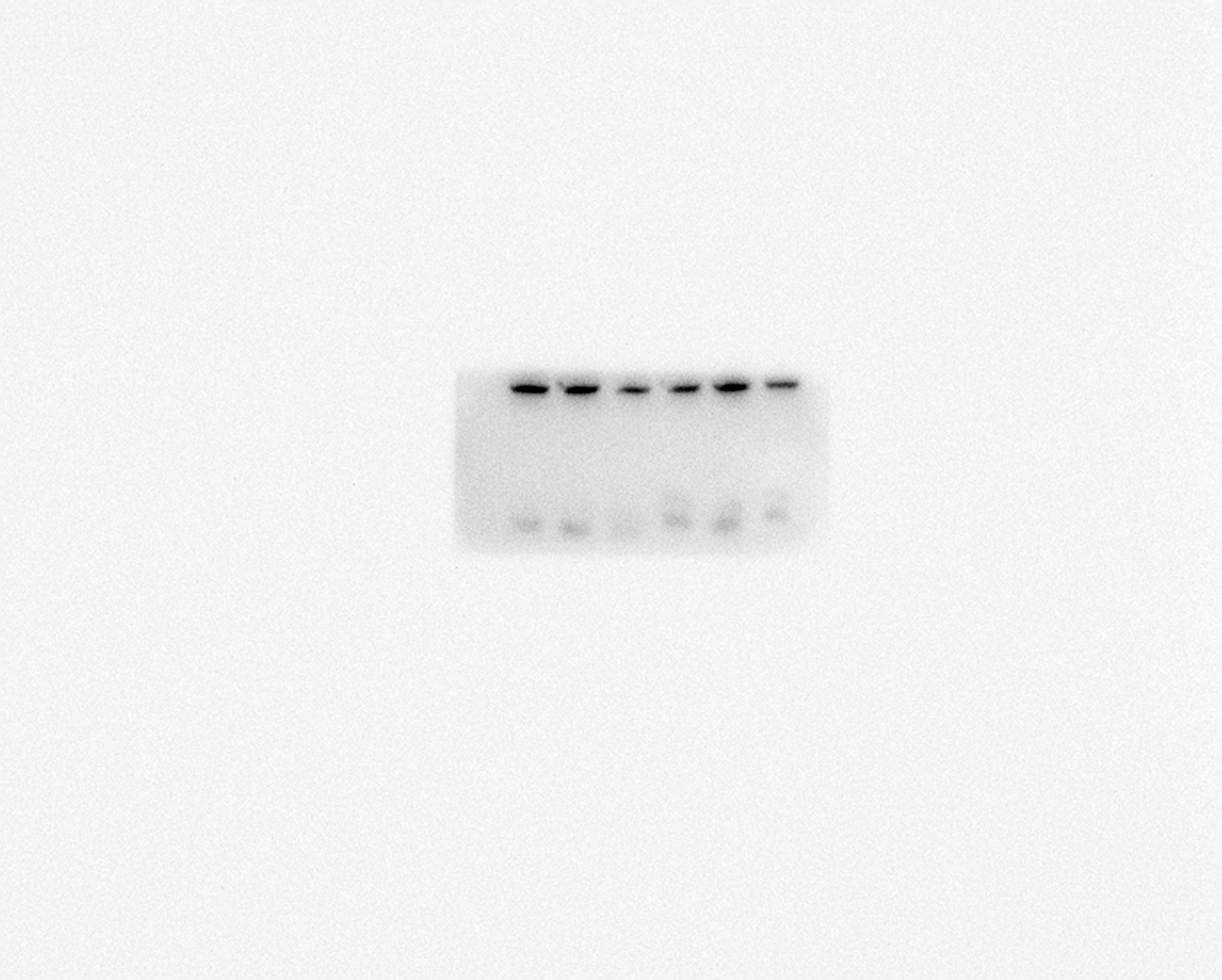

Supplement: Supplementary file 5 [file DataSheet7.ZIP › Co-IP/Input/2/MD2/2.tif]

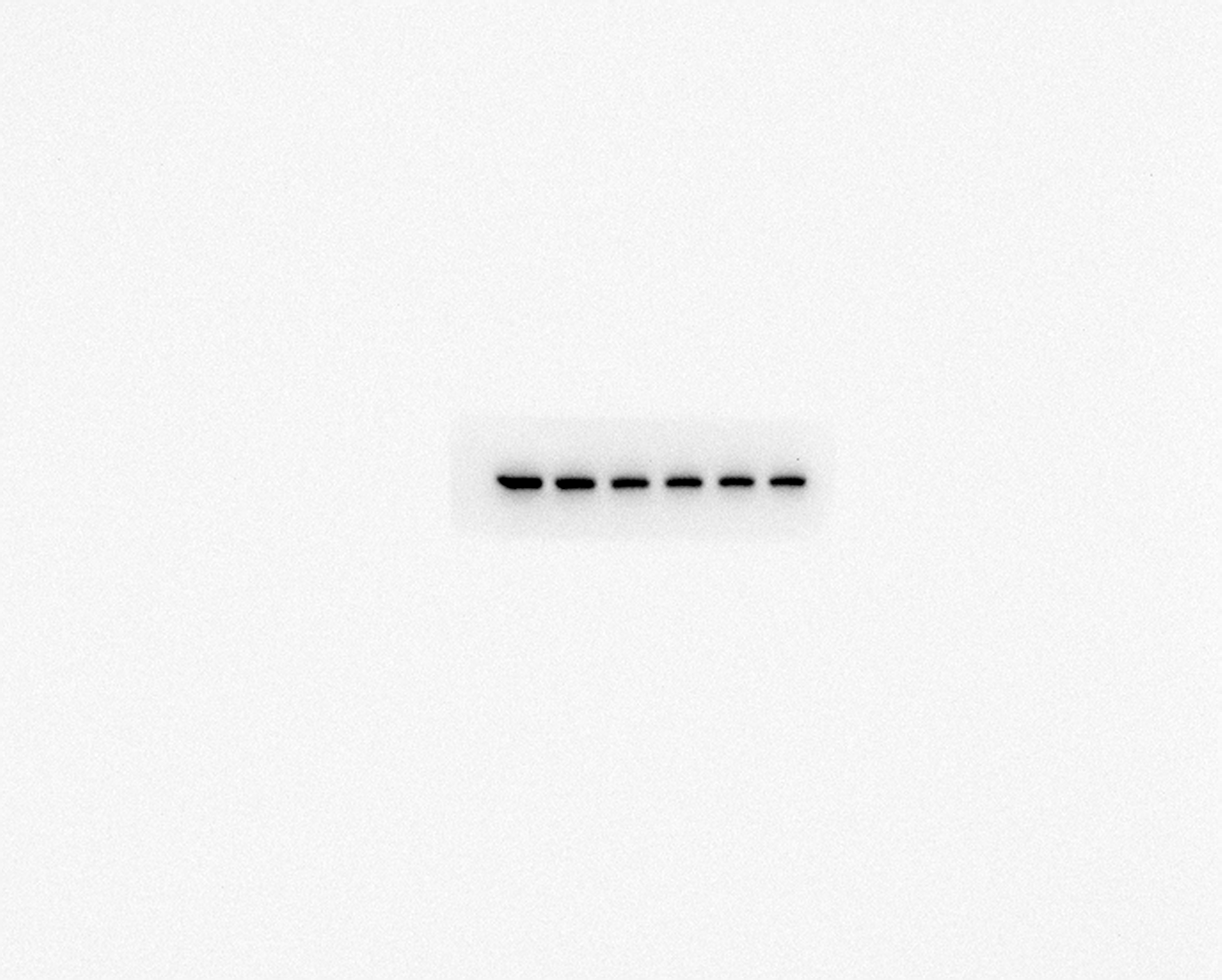

Supplement: Supplementary file 5 [file DataSheet7.ZIP › Co-IP/Input/2/MyD88/1.tif]

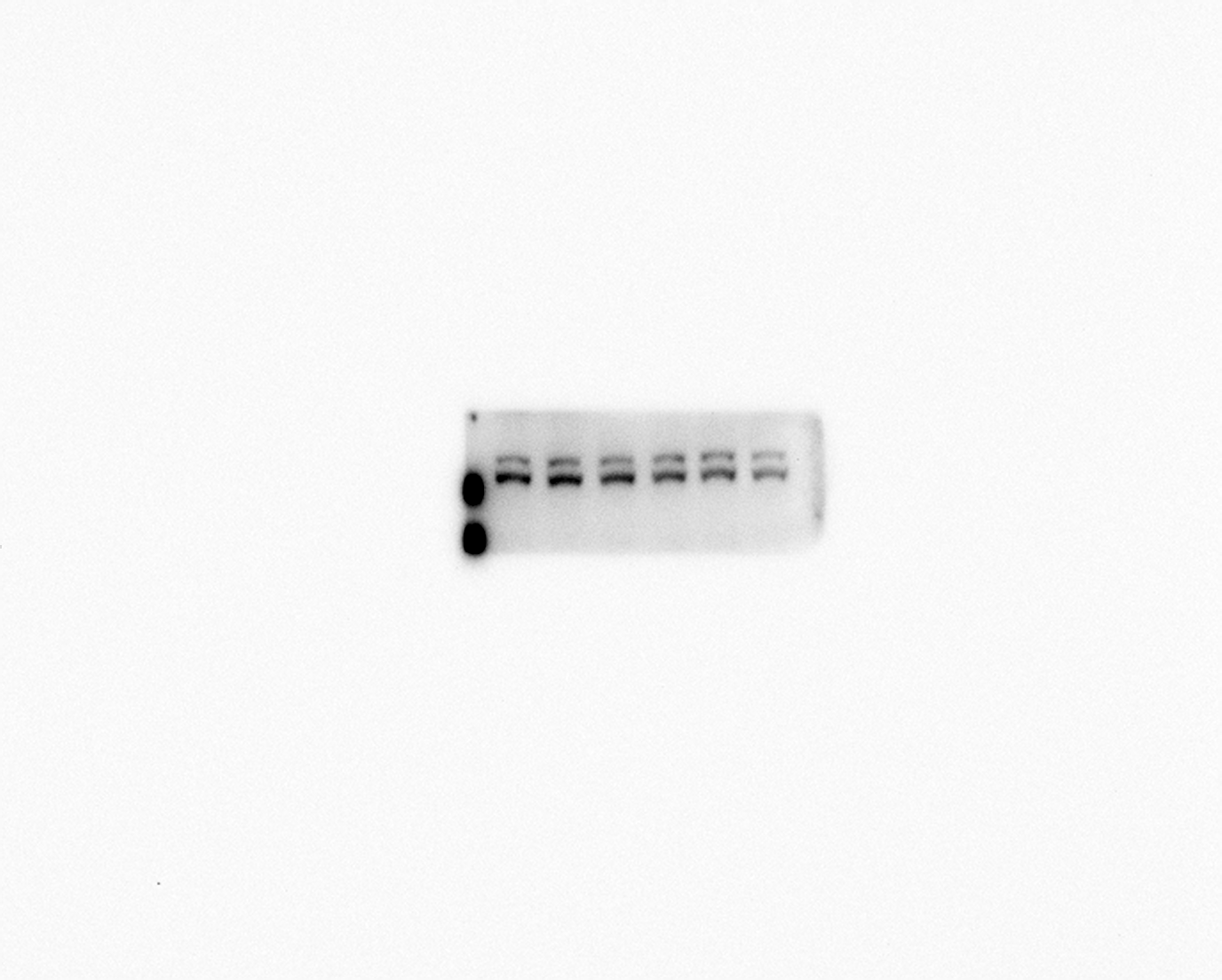

Supplement: Supplementary file 5 [file DataSheet7.ZIP › Co-IP/Input/2/TLR4/2.tif]

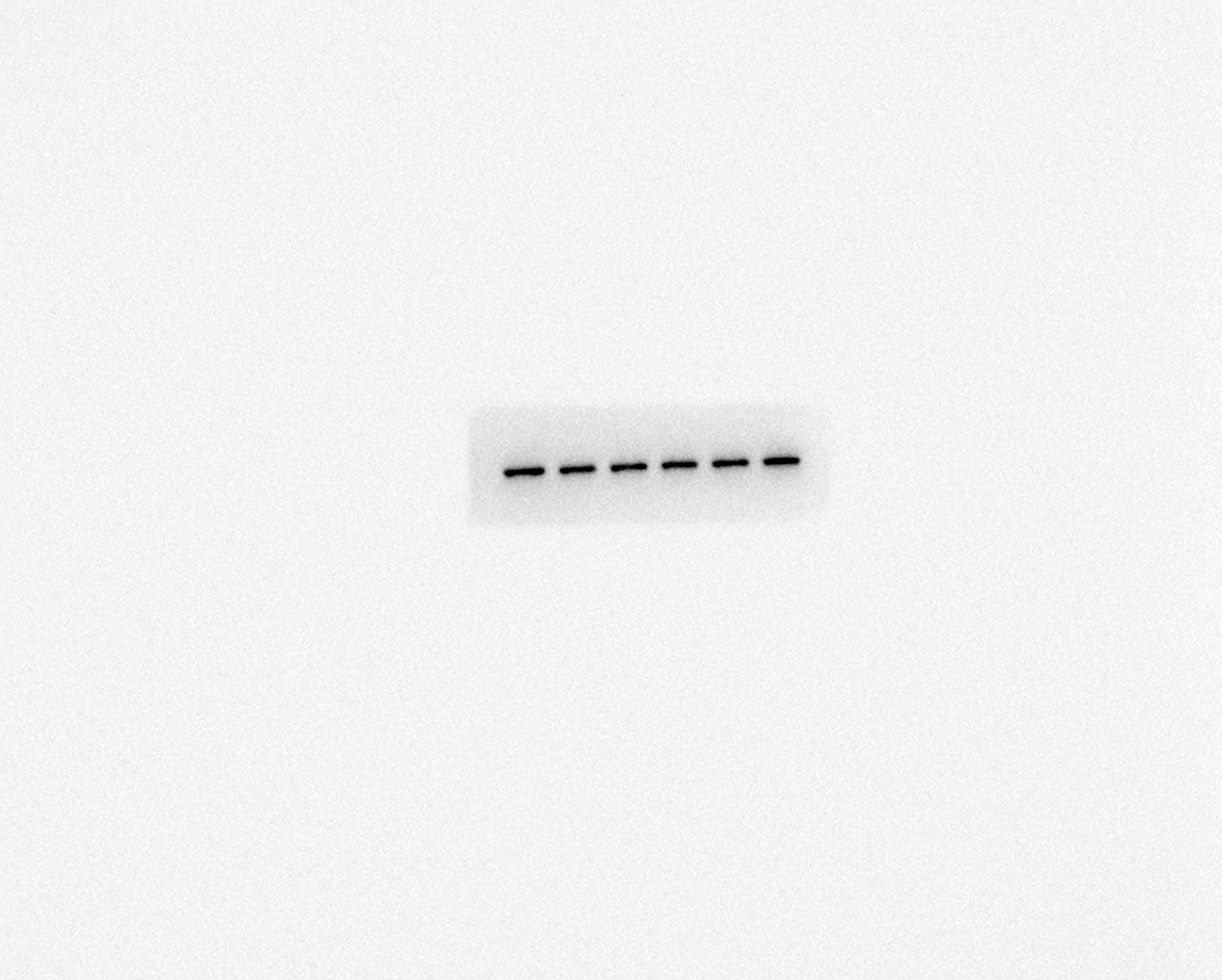

Supplement: Supplementary file 5 [file DataSheet7.ZIP › Co-IP/Input/3/actin/2.tif]

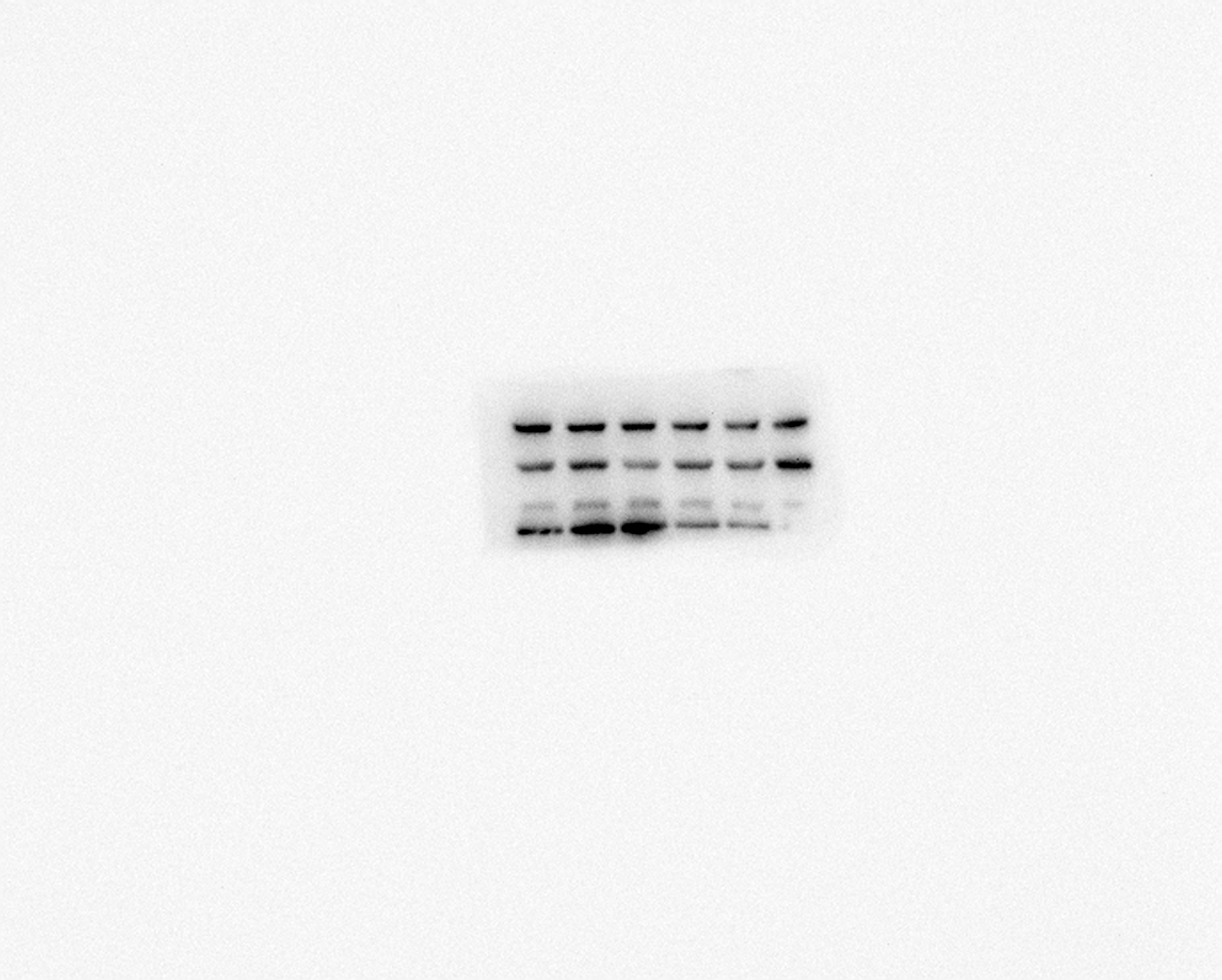

Supplement: Supplementary file 5 [file DataSheet7.ZIP › Co-IP/Input/3/MD2/2.tif]

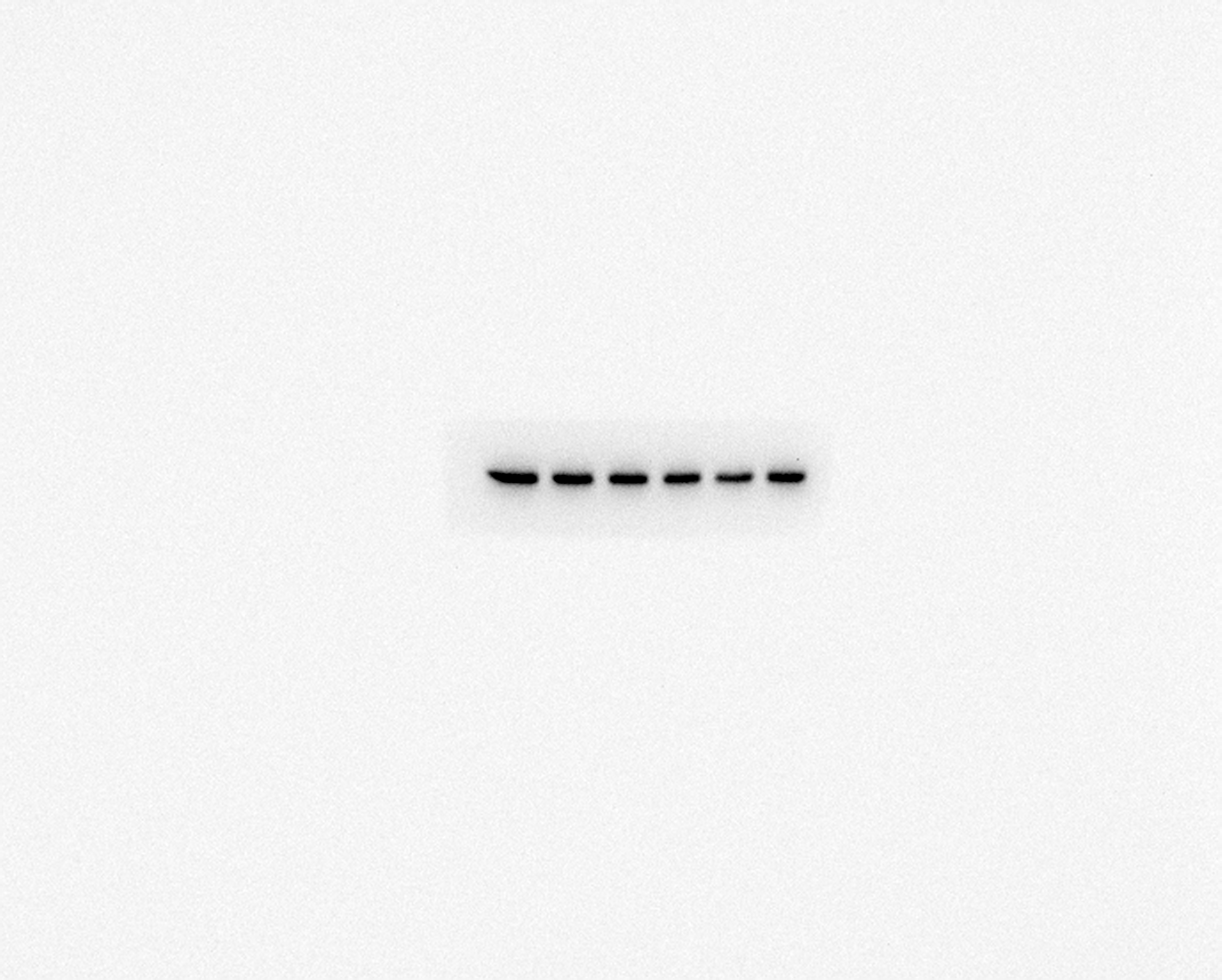

Supplement: Supplementary file 5 [file DataSheet7.ZIP › Co-IP/Input/3/MyD88/1.tif]

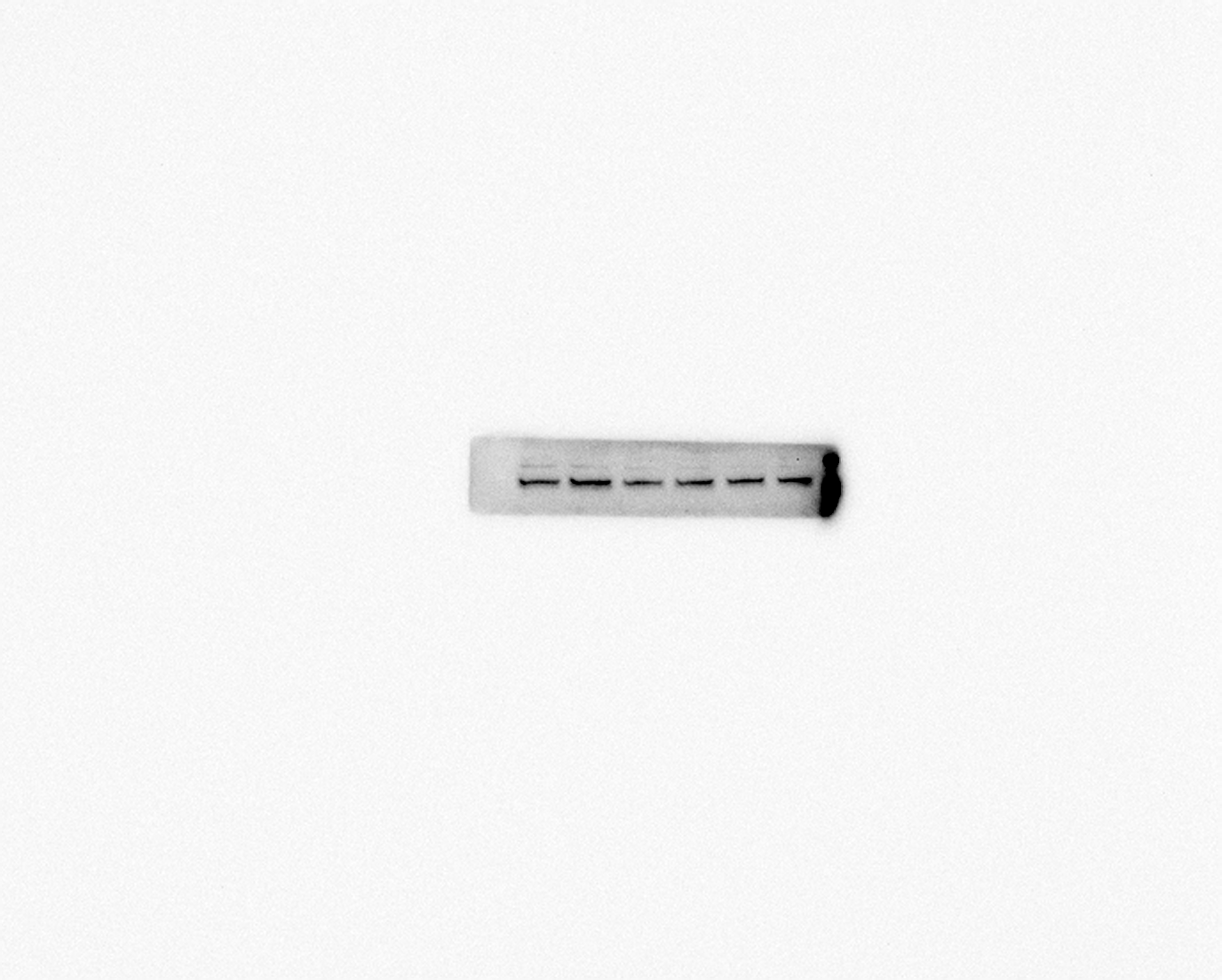

Supplement: Supplementary file 5 [file DataSheet7.ZIP › Co-IP/Input/3/TLR4/2.tif]

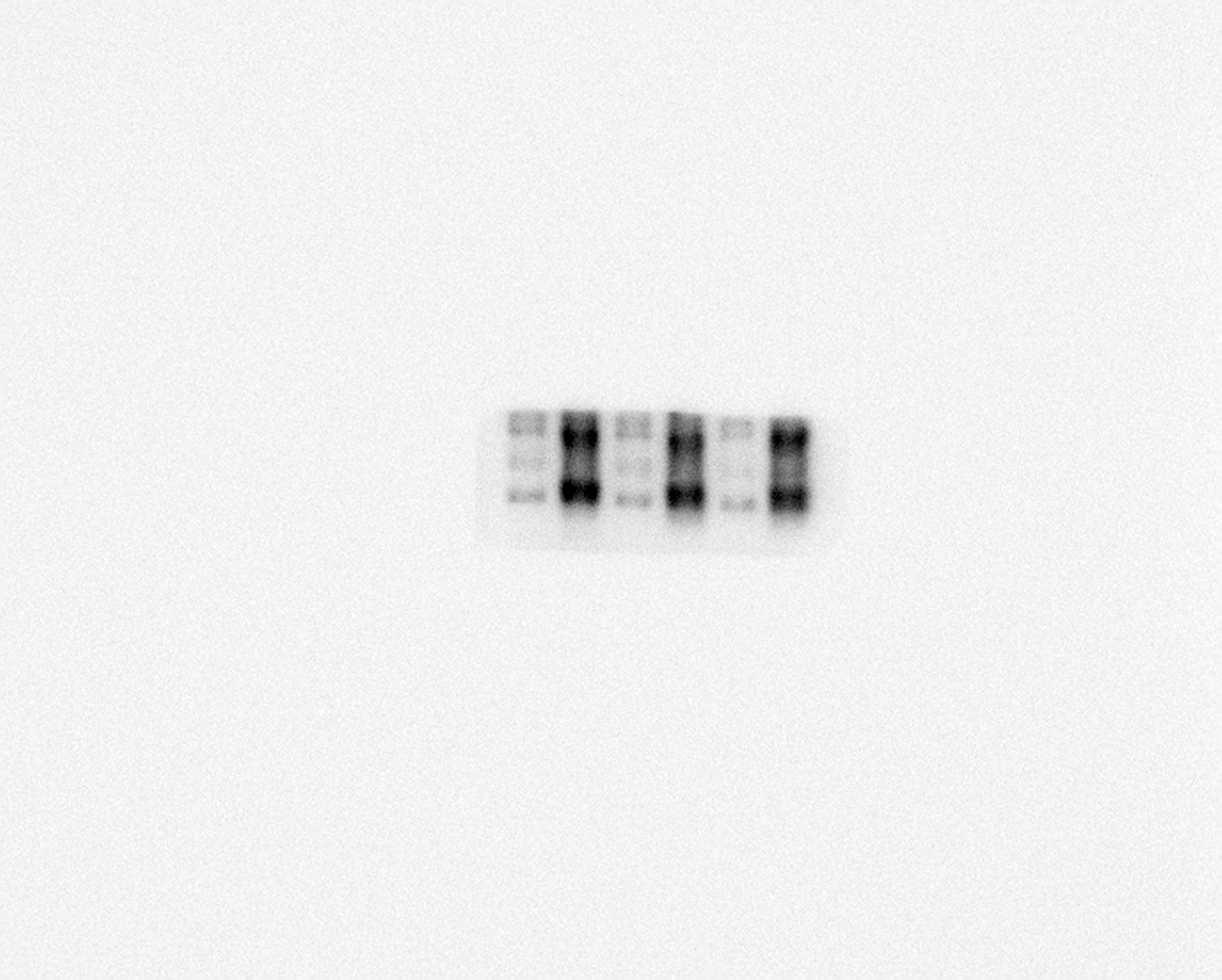

Supplement: Supplementary file 5 [file DataSheet7.ZIP › Co-IP/IP/1/MD2/2.tif]

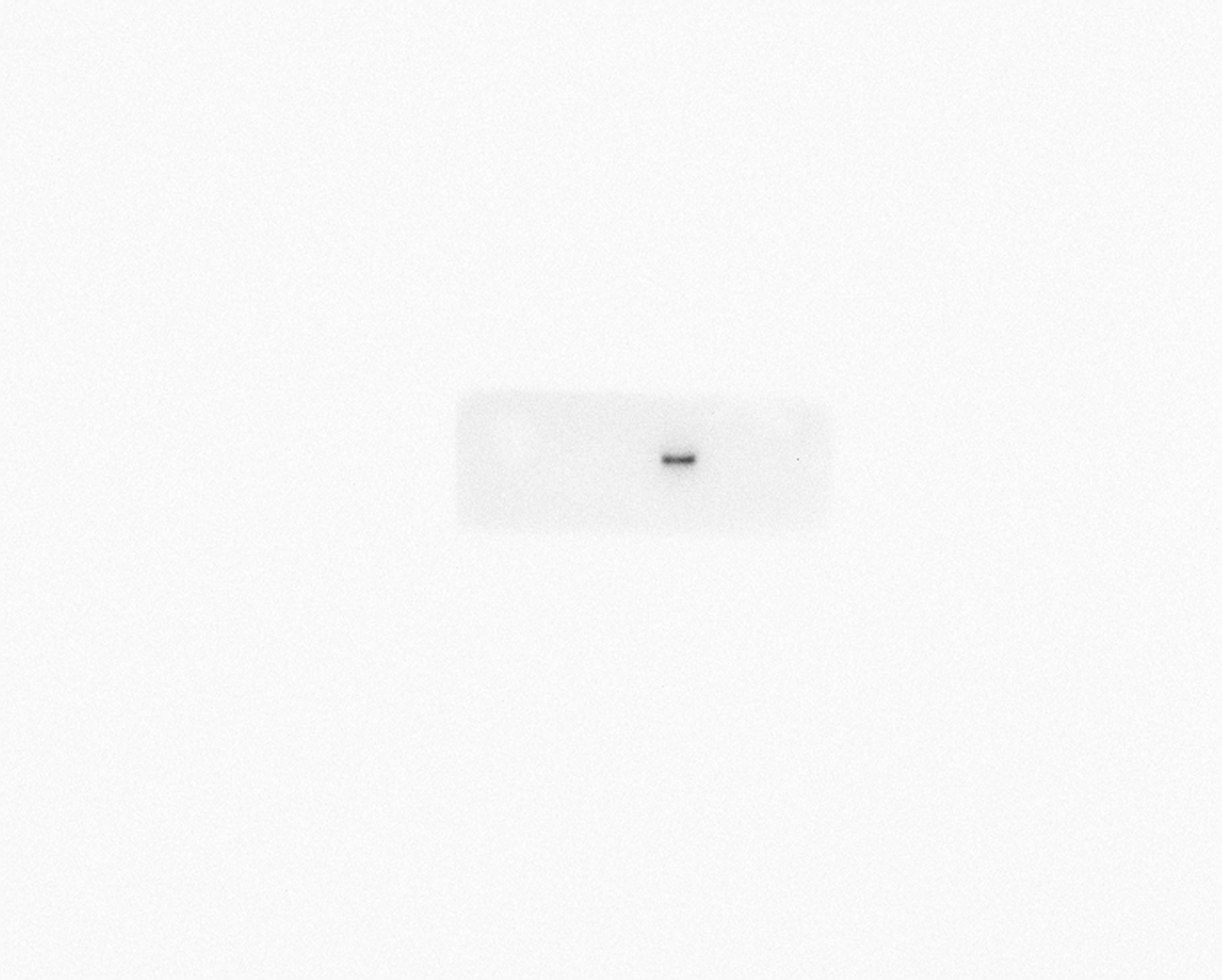

Supplement: Supplementary file 5 [file DataSheet7.ZIP › Co-IP/IP/1/MyD88/2.tif]

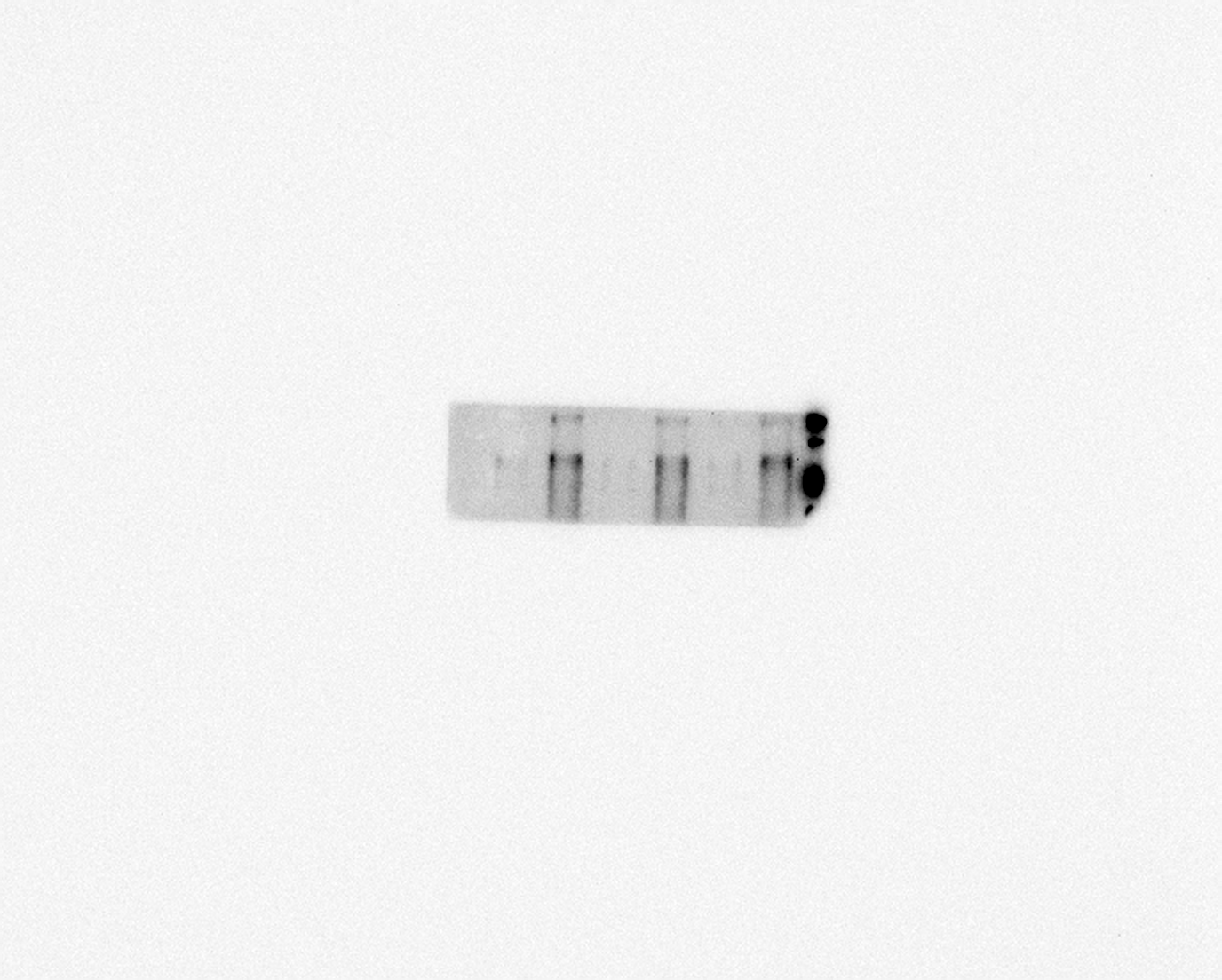

Supplement: Supplementary file 5 [file DataSheet7.ZIP › Co-IP/IP/1/TLR4/2.tif]

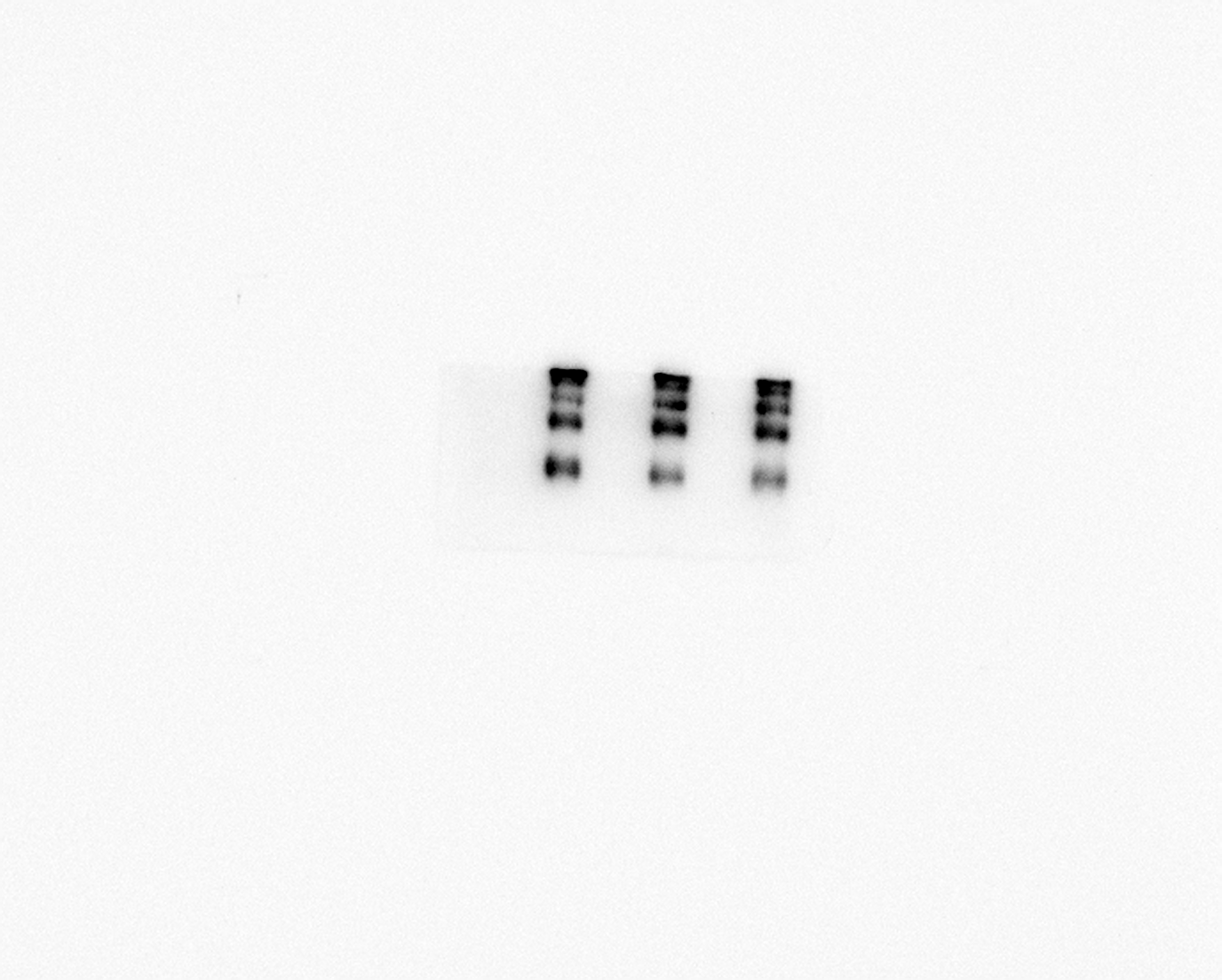

Supplement: Supplementary file 5 [file DataSheet7.ZIP › Co-IP/IP/2/MD2/1.tif]

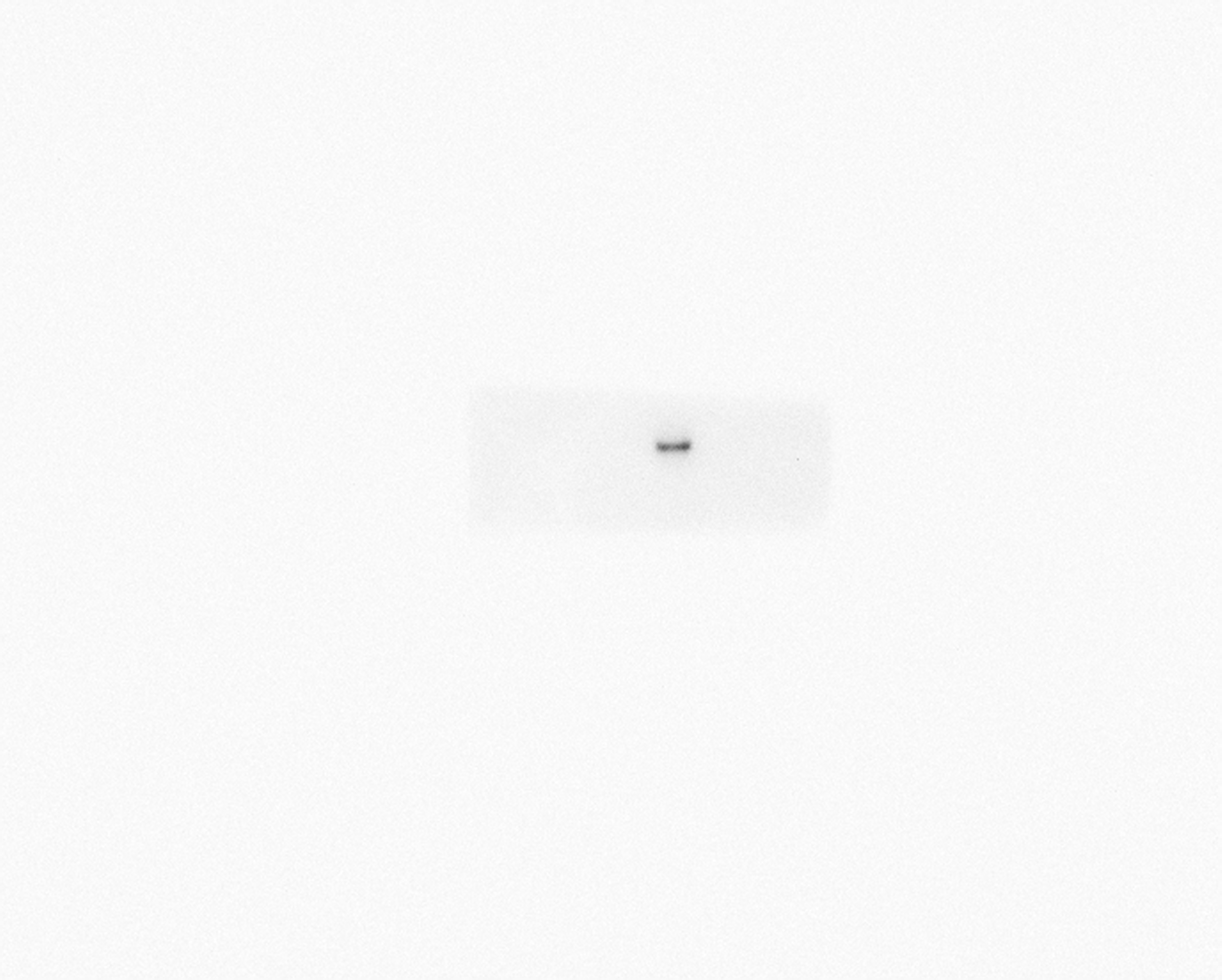

Supplement: Supplementary file 5 [file DataSheet7.ZIP › Co-IP/IP/2/MyD88/2.tif]

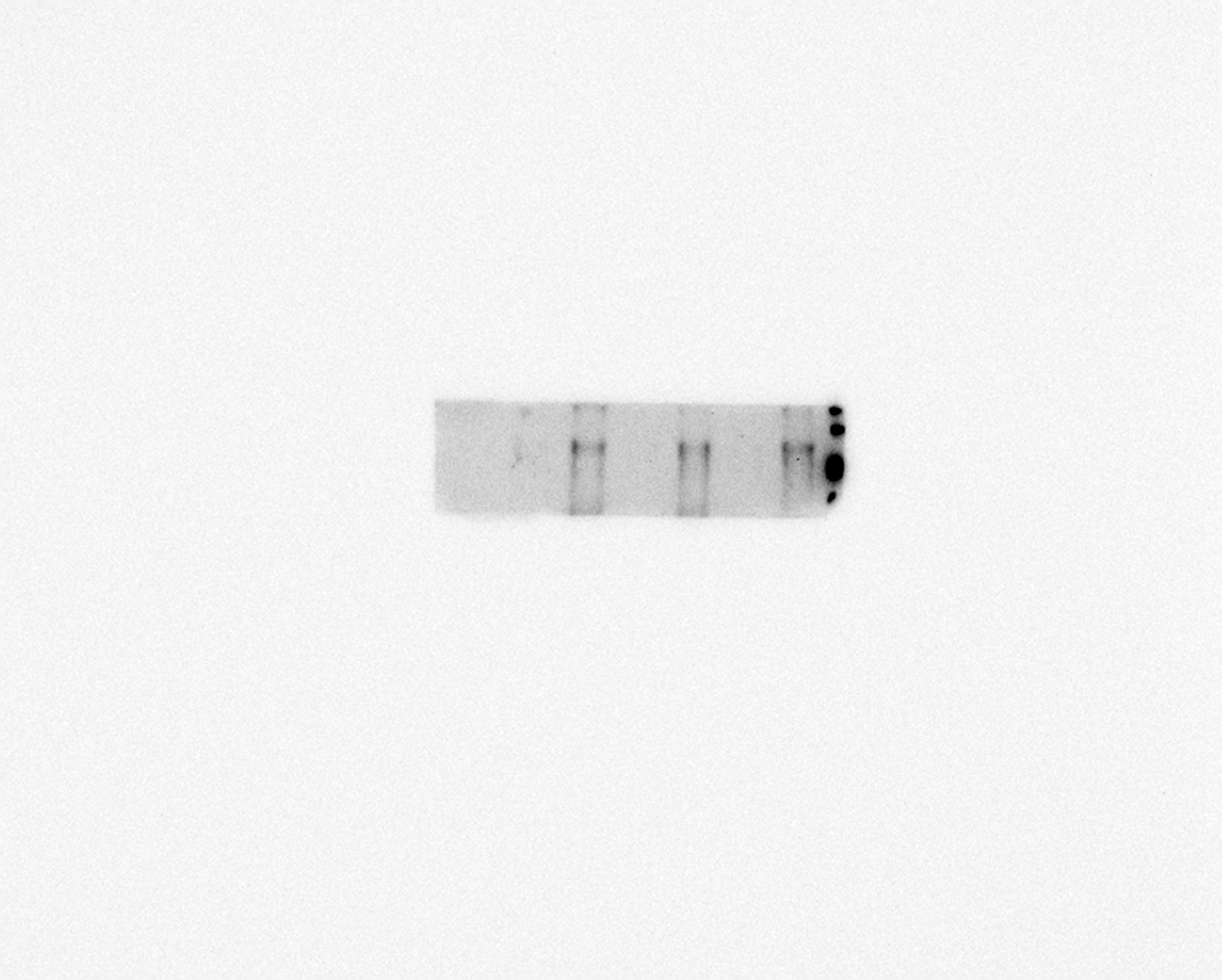

Supplement: Supplementary file 5 [file DataSheet7.ZIP › Co-IP/IP/2/TLR4/2.tif]

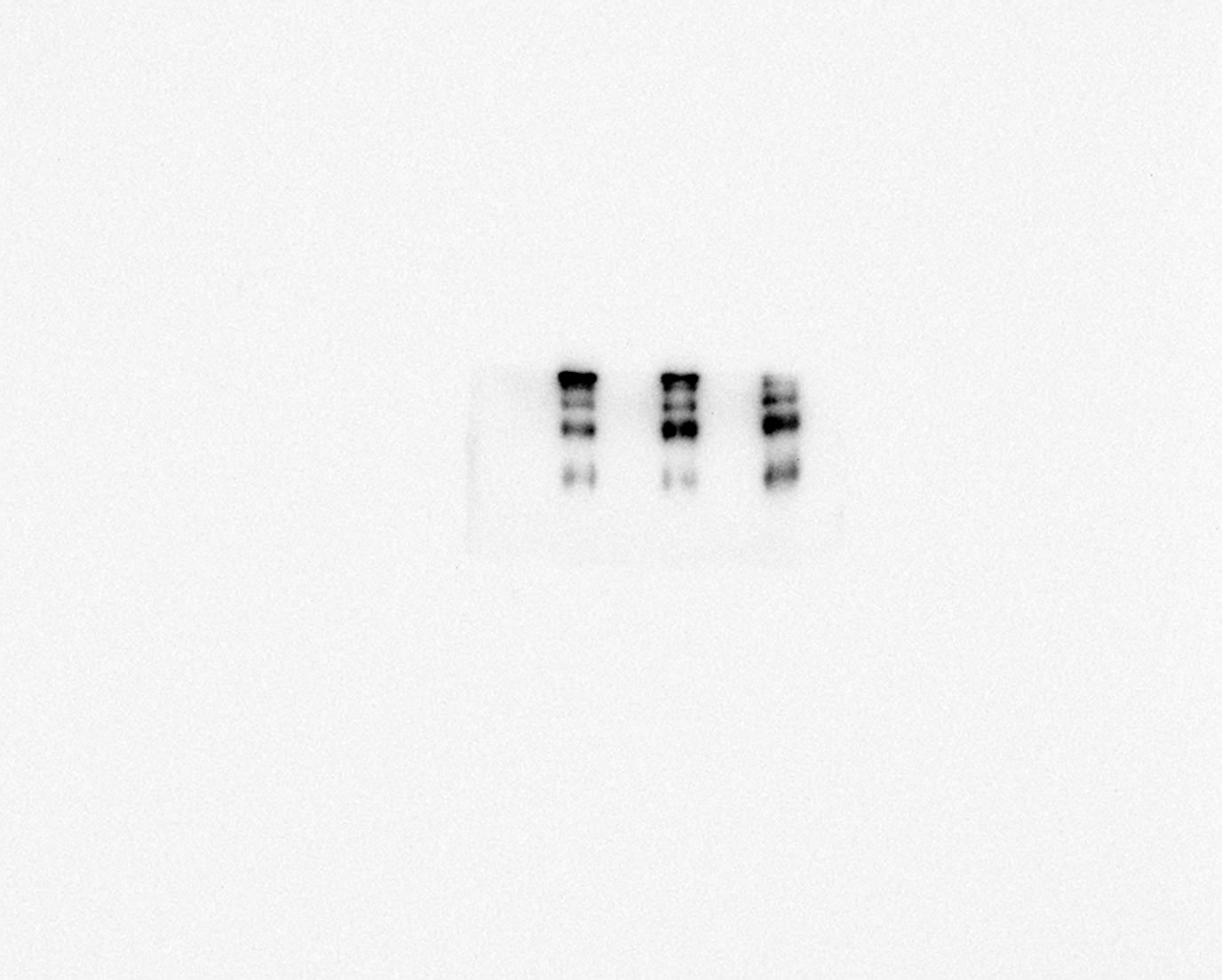

Supplement: Supplementary file 5 [file DataSheet7.ZIP › Co-IP/IP/3/MD2/2.tif]

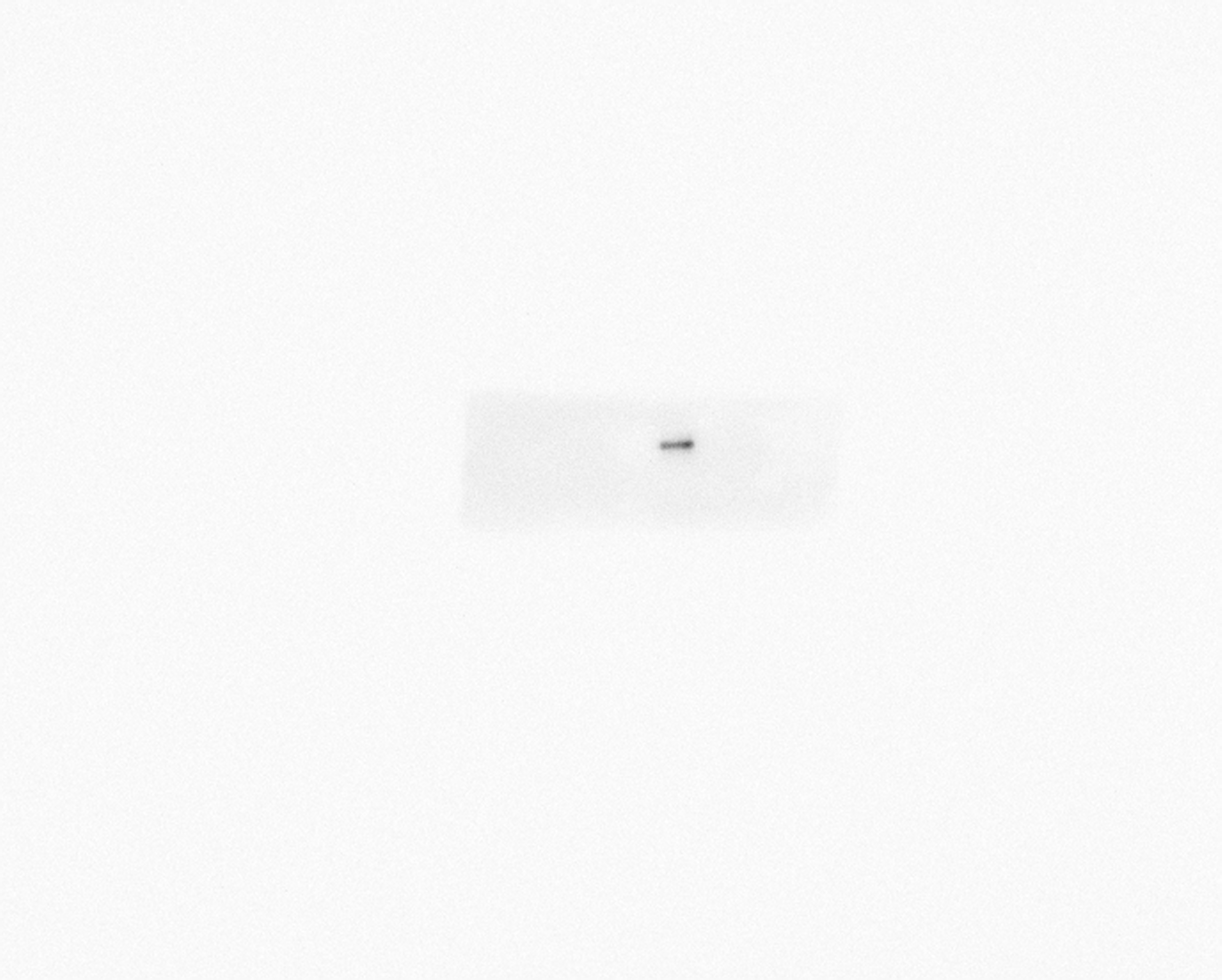

Supplement: Supplementary file 5 [file DataSheet7.ZIP › Co-IP/IP/3/MyD88/2.tif]

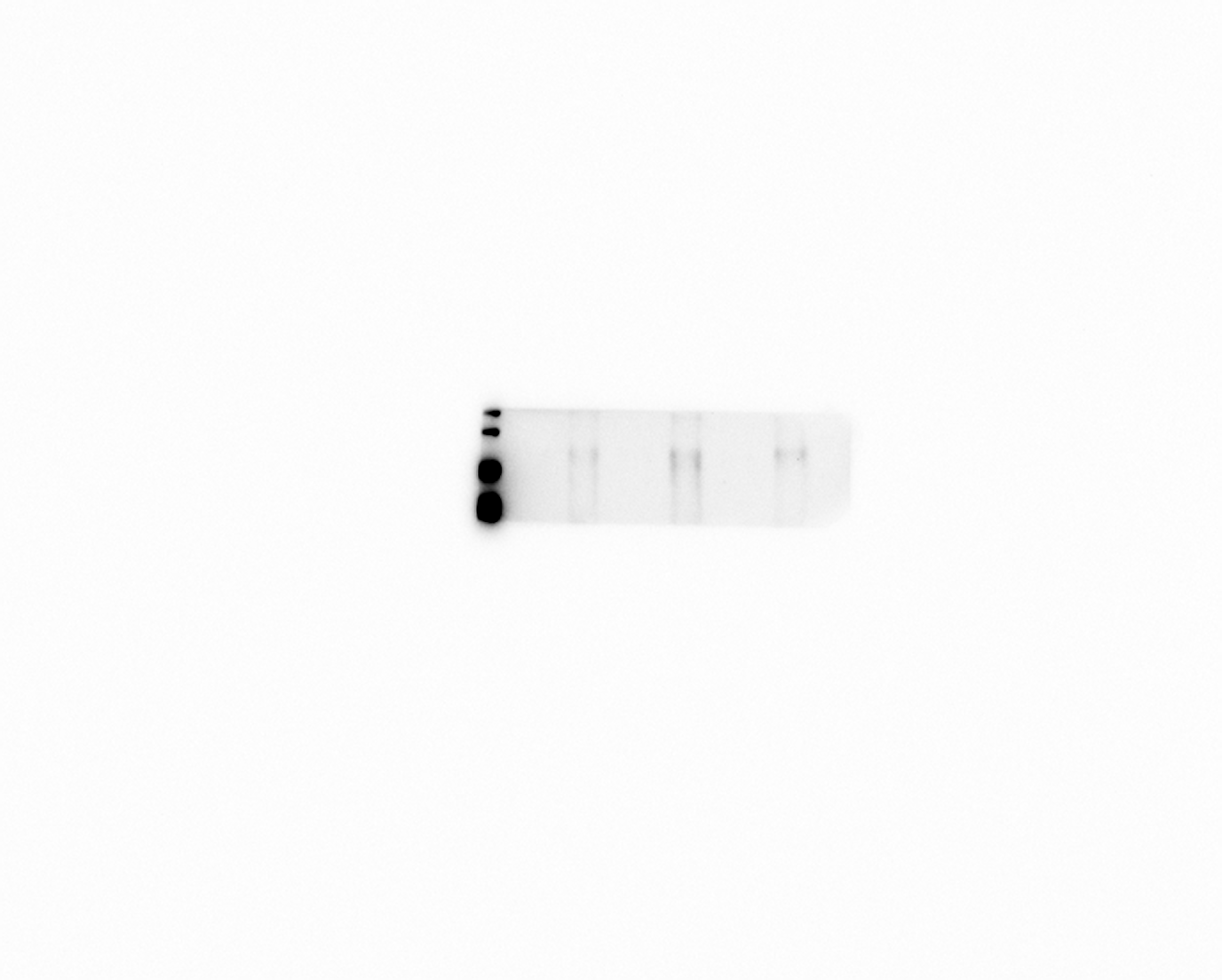

Supplement: Supplementary file 5 [file DataSheet7.ZIP › Co-IP/IP/3/TLR4/2.tif]
