# Supplementary material for: Pentamidine Alleviates Inflammation and Lipopolysaccharide-Induced Sepsis by Inhibiting TLR4 Activation via Targeting MD2
Source: Front Pharmacol. 2022 Feb 23;13:835081. doi: 10.3389/fphar.2022.835081 (PMC8905599; doi:10.3389/fphar.2022.835081)
Supplement: Supplementary file 6 [file DataSheet1.docx]

Supplementary Material

# Supplementary Figures and Tables

## Supplementary Figures


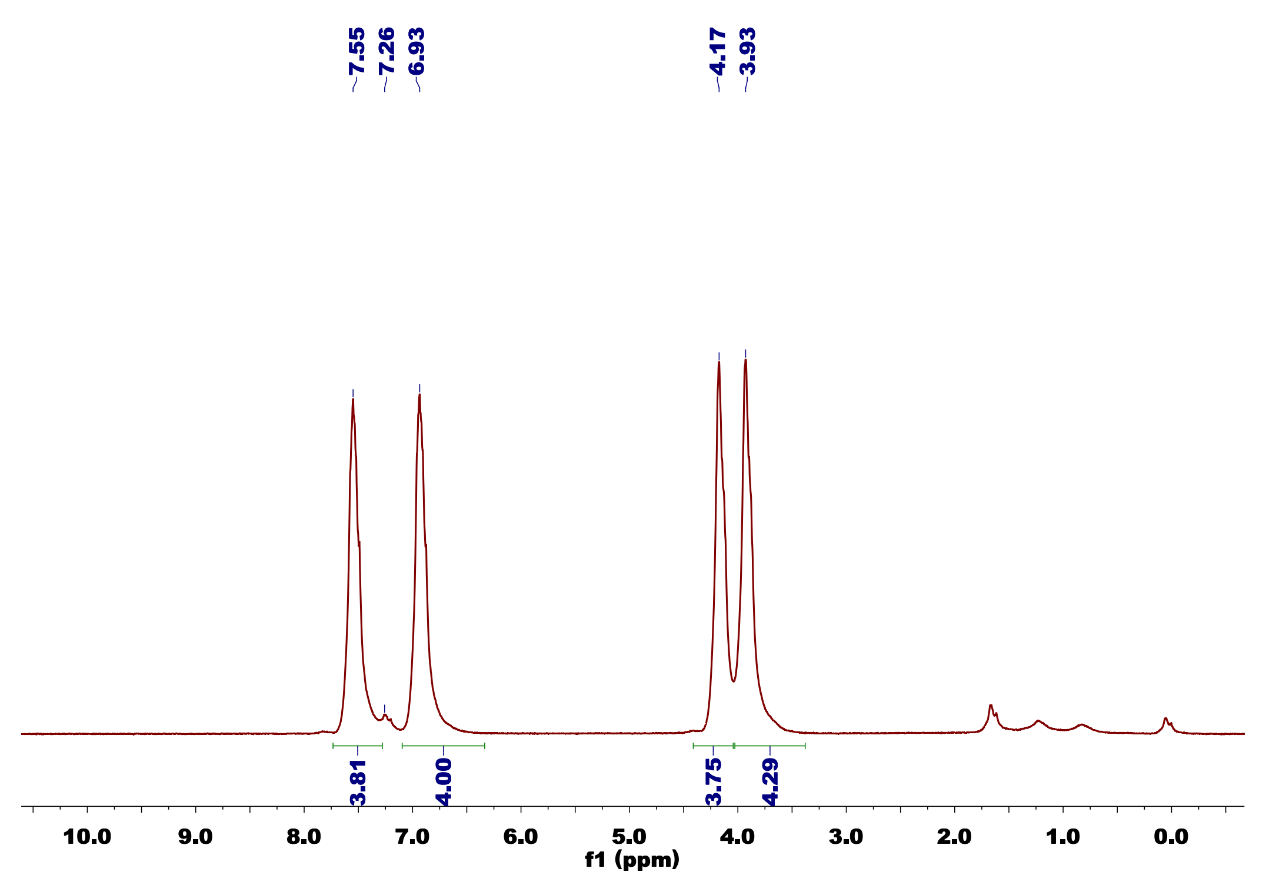

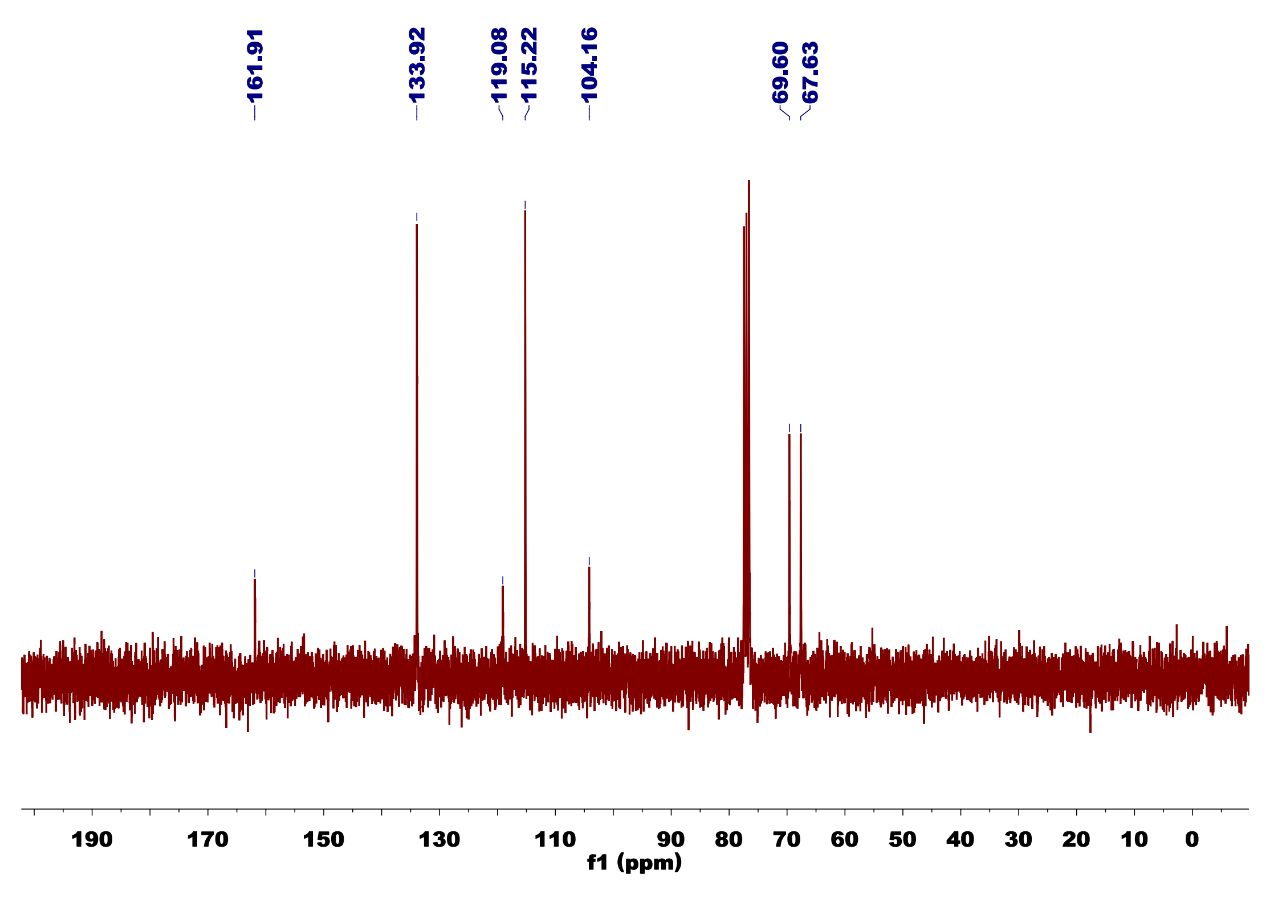


## Supplementary Figure 1. NMR spectra of compound 3.


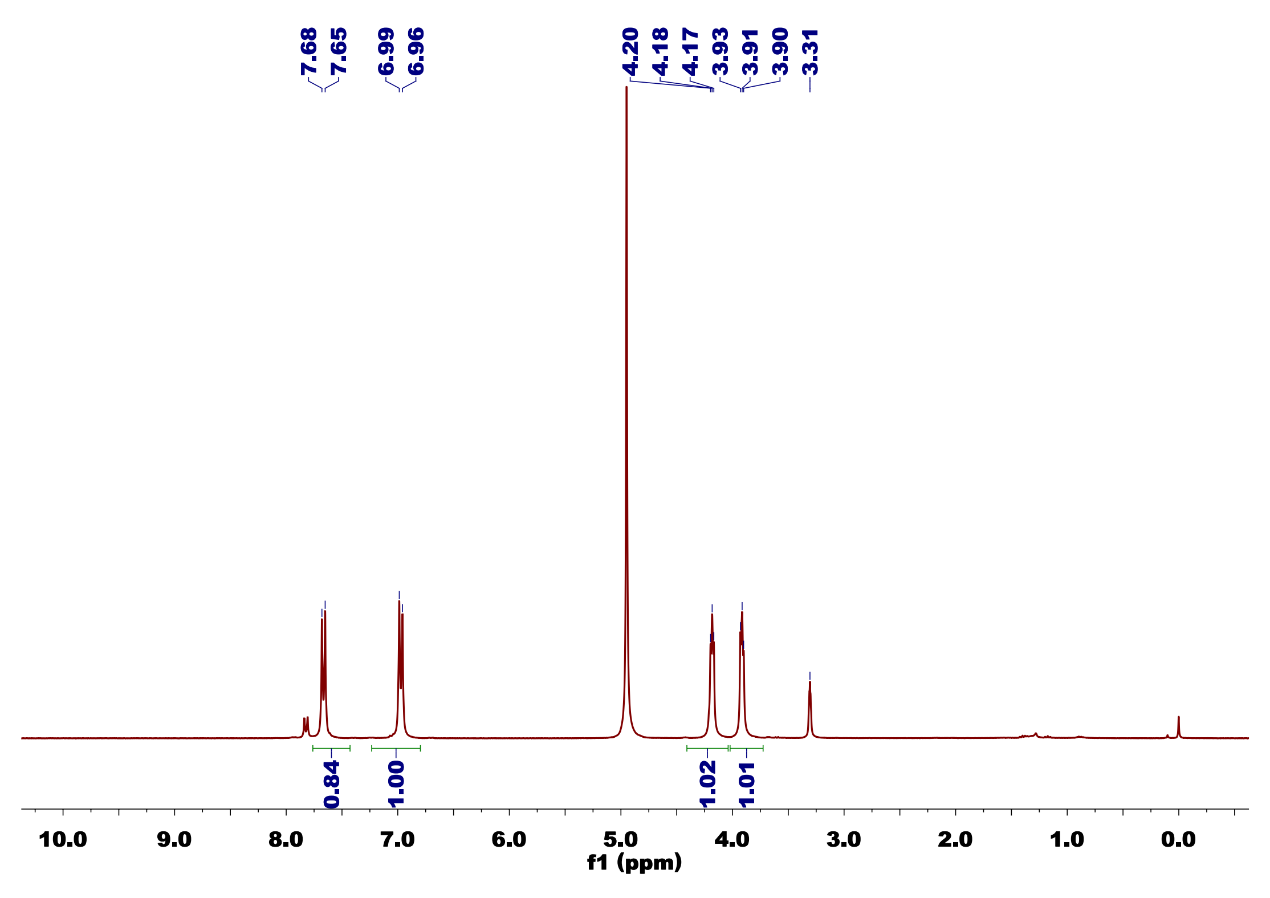


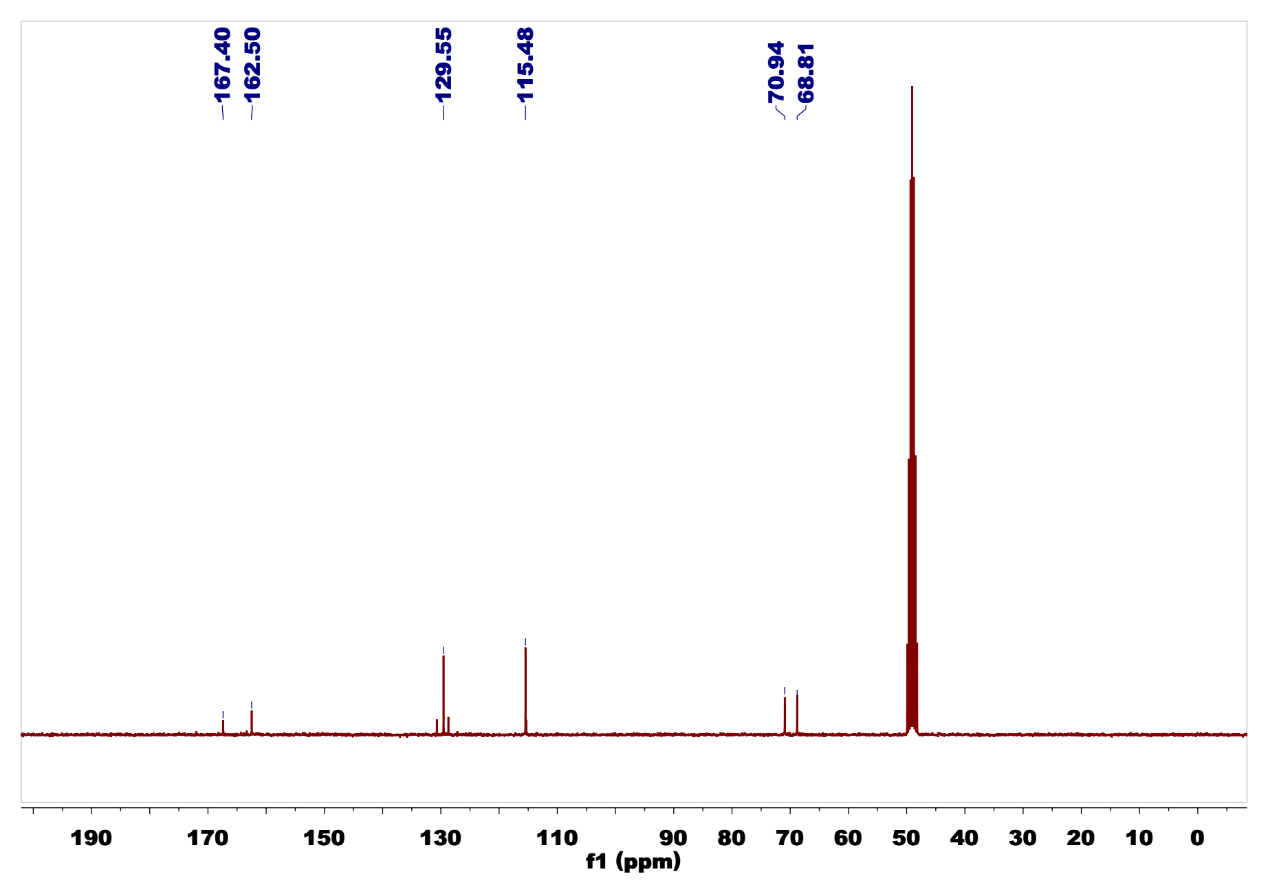


## Supplementary Figure 2. NMR spectra of penta-2.


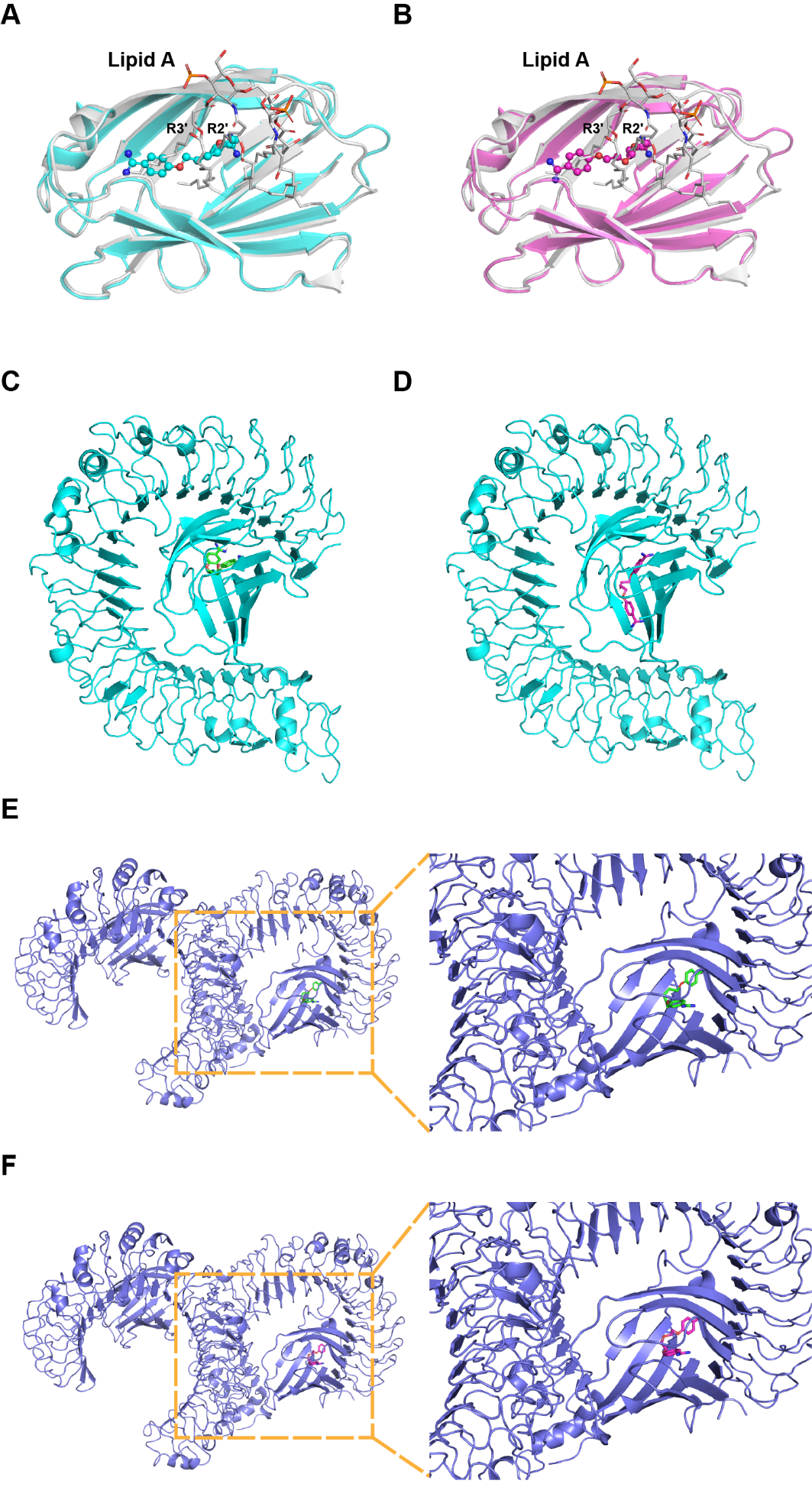


**Supplementary Figure 3. Docking poses of pentamidine or penta-2. (A-B)** Overlap of the best docking pose of pentamidine **(A)** or penta-2 **(B)** with lipid A. LPS binding location was occupied by pentamidine on its acyl chains R3' and R2', and penta-2 on its acyl chains R3', R2'. Lipid A-MD2 was extracted from the active state of TLR4/MD2/LPS complex (PDB ID: 3VQ2) after aligning with compound-docked MD2. MD2 is shown as cartoon, lipid A as sticks, pentamidine and penta-2 as balls-stick. Lipid A-MD2 was colored as gray, pentamidine-MD2 as green, and penta-2-MD2 as magentas. **(C-D)** Best docking pose of pentamidine (**C**) or penta-2 (**D**) in the monomeric mouse TLR4/MD2 complex (PDB ID: 2Z64). **(E-F)** Best docking pose of pentamidine (**E**) or penta-2 (**F**) in the dimeric mouse TLR4/MD2/LPS complex after extracting lipid A from the complex (PDB ID: 3VQ2). Pentamidine or penta-2 stayed in the deep cavity of MD2, far from the activation interface of TLR4/MD2.
